# Exploring Boswellia serrata Triterpenes: A New Frontier in Leukemia Inhibitory Factor Receptor Modulation

**DOI:** 10.1021/acsomega.5c03492

**Published:** 2025-05-21

**Authors:** Claudia Finamore, Carmen Festa, Mattia Cammarota, Lucio Spinelli, Elva Morretta, Chiara Cassiano, Maria Chiara Monti, Silvia Marchianò, Carmen Massa, Federica Moraca, Antonio Lupia, Bruno Catalanotti, Stefano Fiorucci, Angela Zampella, Simona De Marino

**Affiliations:** † Department of Pharmacy, 9307 University of Naples “Federico II”, Via D. Montesano, 49, Naples 80131, Italy; ‡ Department of Medicine and Surgery, 60250 University of Perugia, Piazza L. Severi, 1, Perugia 06132, Italy; § Department of Life and Enviromental Science, 3111 University of Cagliari, S.P. 8 Monserrato—Sestu Km 0,700—Monserrato, Cagliari 09042, Italy

## Abstract

Boswellia serrata, commonly known as Indian olibanum or Indian frankincense, is a medicinal plant recognized for its significant anti-inflammatory, analgesic, and anticancer activities. In our investigation, the activity of its *n*-hexane extract was evaluated on targets never explored for *Boswellia*, mainly involved in hepatic fibrosis and cancer development. Since this extract exhibited a significant antagonistic activity on the interaction between leukemia inhibitory factor (LIF) and its receptor (LIFR), it was subjected to an untargeted metabolomic analysis using a high-resolution mass spectrometry-based approach combined with molecular networking. An unambiguous assignment of several *Boswellia* triterpenoid metabolites was then achieved upon isolation and NMR spectroscopic investigation to accurately identify the bioactive *Boswellia* components responsible for the *n*-hexane extract activity on the LIF/LIFR system. Key active metabolites, including boswellic acids and their derivatives and a small library of semisynthetic analogues, demonstrated potential inhibitory activity toward LIF/LIFR interaction. In particular, α-boswellic acid (**1**) emerged as a LIFR antagonist, able to reduce the expression of col1α1 and α-SMA in LX-2 cells. Furthermore, computational studies highlighted the role of the carboxyl group in engaging a network of electrostatic and hydrogen bond interactions within residues of human LIFR (*h*LIFR) binding site. This finding suggests the potential use of *Boswellia* in hepatic fibrosis and sheds light on a relatively novel target for liver fibrosis therapy.

## Graphical abstract

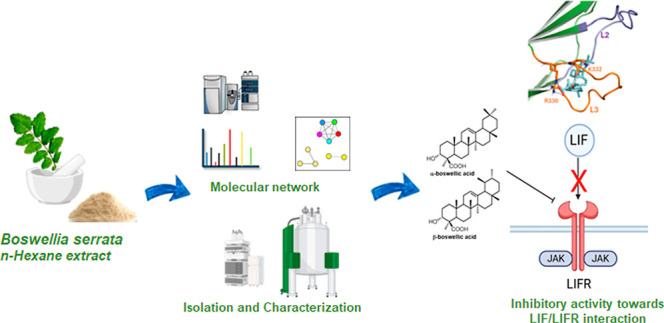

## Introduction

Plants are a primary source of molecules with immense diversity, complexity, and variety of pharmacological activities. Their potential continues to attract interest from the scientific community, making target identification a crucial step in elucidating the mechanisms by which plant components exert their functions.

Boswellia serrata, belonging to the Burseraceae family, is a tree native of India and the Arabian Peninsula.^1^ Commonly known as Indian frankincense, this medicinal plant is recognized for its numerous health benefits, particularly in inflammation and cancer treatment.

In literature, there is accumulating evidence for anticancer and anti-inflammatory effects providing mechanistic insights in the modes of action both *in vitro* and *in vivo* experiments. The anticancer effect targets multiple molecular pathways, including kinases, transcription factors, enzymes, receptors, growth factors, and other elements involved in cell survival and proliferation.^2^ This effect is attributed to boswellic acids, particularly to acetyl-11-keto-β-boswellic acid (AKBA), which plays a key role in mediating anticancer properties of Indian frankincense representing a promising drug in cancer therapy.

The extracts from *Boswellia* are being studied for the treatment of chronic inflammatory conditions such as arthritis, asthma, and inflammatory bowel diseases (IBD).

For centuries, in traditional Ayurvedic medicine, resin has been used for its anti-inflammatory properties, and the acidic fractions from gum resins of different *Boswellia* spp. were found to significantly inhibit the activity of enzymes such as 5-lipoxygenase (5-LOX) and microsomal prostaglandin E synthase-1 (mPGES-1).^3^

An impressive number of compounds were isolated from plants of the genus *Boswellia*, comprising 24 species and exhibiting fascinating structural diversity, unusual chemical structures, and a range of potential health benefits.

As part of our interest on plants for the identification of possible new bioactive molecules, we proceeded in the isolation of secondary metabolites from B. serrata. This resulted in the identification of two new compounds (**6** and **20**) together with known pentacyclic and tetracyclic triterpenes belonging to the ursane, oleanane, lupane, and tirucallane type. Among pentacyclic triterpenes of the oleanane and ursane groups, we have identified α- and β-boswellic acids (**1** and **2**)^4^ and their 3-*O*-acetyl analogues (**3** and **4**),^5,6^ urs-12-en-3α,24-diol (**5**),^7^ olean-12-en-3α,24-diol (**6**),^8,9^ 11-keto-β-boswellic acid (**7**),^4,10^ and 9,11-dehydro-β-boswellic acid (**8**)^10^ (Figure 1) along with the neutral metabolites α-amyrin (**9**),^11^ 3-*epi*-α-amyrin (**10**),^11^ β-amyrin (**11**),^12^ 3-*epi*-β-amyrin (**12**)^13^ (Figure 2), and tetracyclic triterpenes with a tirucal (**13–17**)^5,14–16^ or lupan (**18–20**)^17^ skeleton (Figure 3). Additionally, the main metabolites, α- and β-boswellic acid were subjected to conjugation with glycine and taurine, affording the semisynthetic derivatives **21–24** (Figure 4). This modification, as previously reported,^18^ improves the antagonism profile on LIFR.

**Figure 1:**
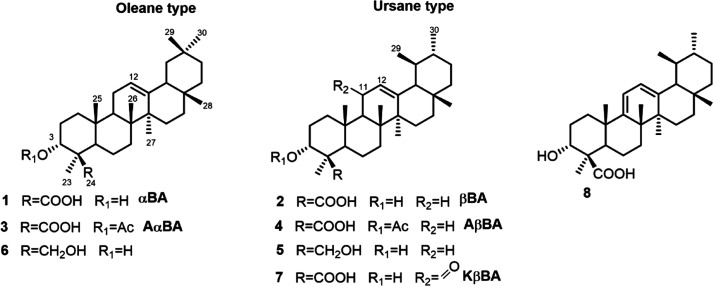
α- and β-boswellic acids and their analogues.

**Figure 2:**
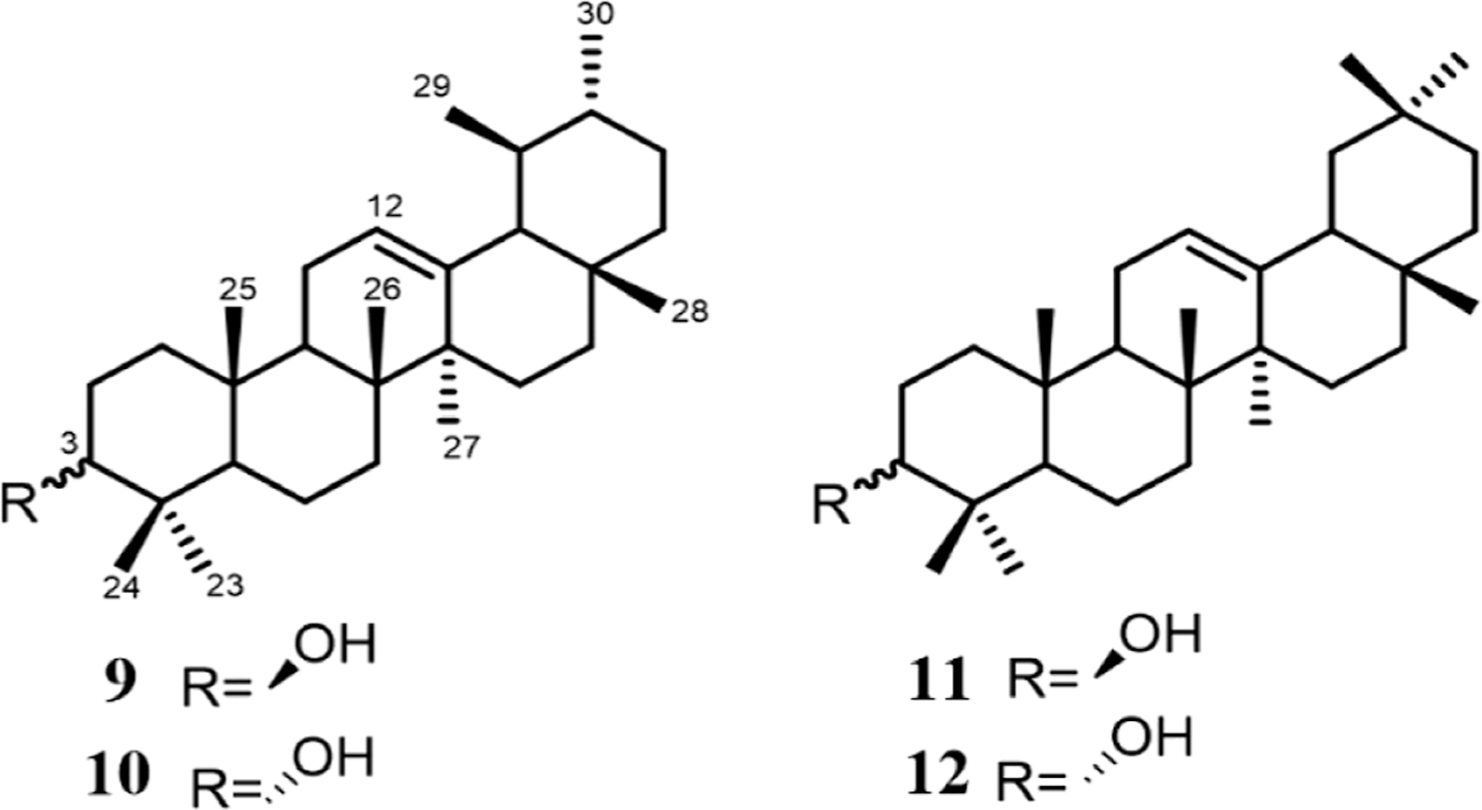
α- and β-amyrines and their 3-epi analogues.

**Figure 3:**
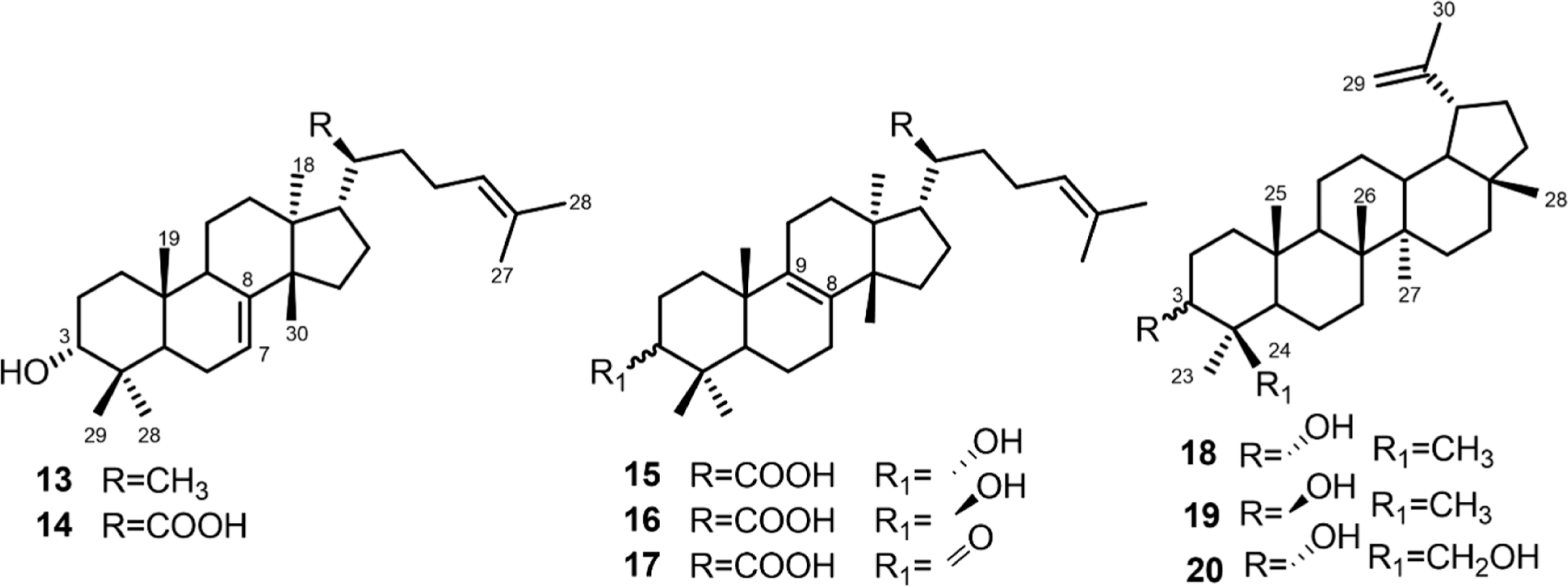
Compounds with a tirucal or lupan skeleton.

**Figure 4:**
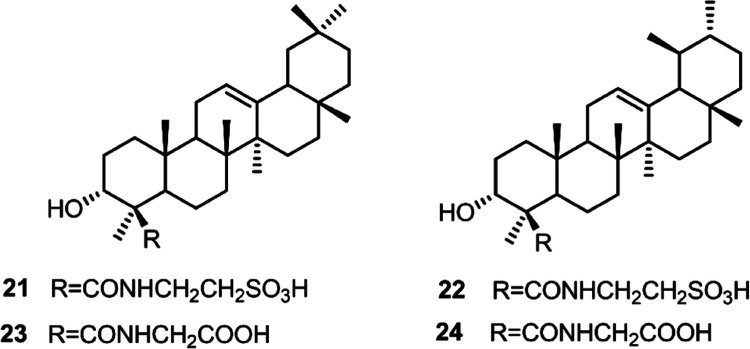
Semisynthetic compounds **21–24**.

The use of *Boswellia* extract in inflammatory bowel disease (IBD) prompted us to explore its potential activity toward two known targets involved in inflammation and immunomodulation in intestinal disorders, the farnesoid X receptor (FXR) and the G protein-coupled bile acid receptor 1 (GPBAR1), and toward the leukemia inhibitory factor (LIF) receptor (LIFR), a tyrosine kinase receptor highly expressed in entero-hepatic tissues and gastrointestinal cancers.^19^

With this background in mind, we have embarked in a campaign designed to identify novel potential targets for *Boswellia*. In the first set of experiments, we have investigated whether the *Boswellia* *n*-hexane extract (BhE) modulates GPBAR1, FXR, and/or LIFR. The results demonstrated that BhE exerts a robust inhibitory activity on LIFR, whereas it was inactive toward both FXR and GPBAR1.

LIFR and its endogenous ligand, LIF, are frequently overexpressed in many solid tumors, making the LIF/LIFR axis a promising target for cancer therapy.^19^ This signaling pathway activates oncogenic pathways such as JAK/STAT3, MAPK, AKT, and mTOR, contributing to tumor growth, progression, metastasis, stemness, and resistance to therapy. Fibrotic diseases, marked by excessive tissue scarring, represent another area where LIFR inhibitors could have a profound impact.^20^ Indeed, identifying potential therapeutic targets for liver fibrosis is important since liver fibrosis can advance to serious liver diseases like cirrhosis and hepatocellular carcinoma.^21^ Inhibiting LIFR could help to mitigate fibrosis and enhance tissue function. The LIFR inhibitors have demonstrated potential in various therapeutic areas, although much of the research remains in the preclinical or early clinical stages.

Following, a detailed characterization of the secondary metabolites responsible for the observed activity of the BhE was performed through a dereplication process using an LC–MS/MS and molecular networking (MN) combined approach together with classic isolation and chemical characterization procedures. Moreover, in this paper, we reported the structural characterization of two new compounds (**6** and **20**) and demonstrated for the first time that some boswellic and tirucallic acids act as antagonists of LIFR, elucidating also their binding mode in human leukemia inhibitory factor receptor (hLIFR) by docking calculations and molecular dynamics (MD).

The identification of plant-derived LIF/LIFR antagonists provides further evidence of the antitumor and antifibrotic potential of *Boswellia* extracts and reveals bioactive metabolites for cancer and liver fibrosis therapy.

## Results and Discussion

To explore new potential activities of *Boswellia* *n*-hexane extract (BhE), it was tested in our well-consolidated array of *in vitro* and *in cell* assays. As clearly reported in Figure 5A, BhE showed a first–rate profile in inhibiting the binding between LIF and LIFR, already at very low concentrations and with an IC_50_ of 1.18 μg/mL. Accordingly, BhE was also active in inhibiting LIFR transactivation in HepG2 cells, already at 1 μg/mL (Figure 5B). In parallel, BhE was also tested on other biologically relevant systems: it does not influence either the SRC-1 coactivator recruitment of FXR or the GPBAR1 transactivation in HEK293T cells (Figure S1).

**Figure 5:**
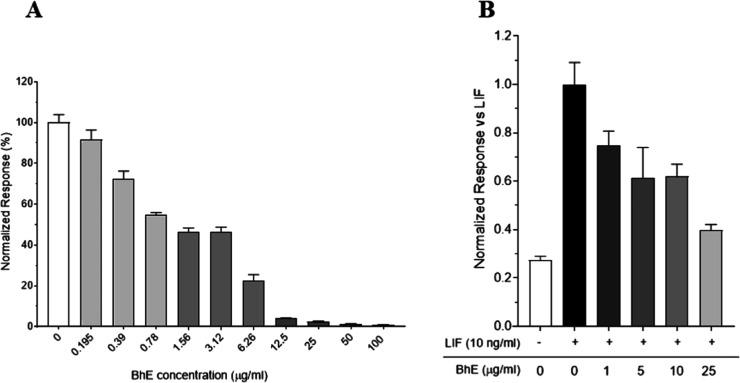
Investigation on *Boswellia* *n*-hexane extract (BhE) activities: (A) LIF-LIFR complex displacement induced by BhE at different concentrations as measured by Alpha Screen assay; (B) LIFR transactivation assay in HepG2 cells stimulated with LIF at 10 ng/mL and treated with increasing concentrations of BhE.

Therefore, to fully characterize the BhE and to identify which component(s) was mainly responsible for the different bioactivities reported above, the ultrahigh-performance liquid chromatography tandem mass spectrometry (UHPLC-MS/MS) was employed as a powerful tool to elucidate its metabolic fingerprint. Indeed, high-resolution mass spectra were acquired and, to achieve tentative identification based on the LC–MS/MS analysis, accurate mass fragmentation patterns and retention times were considered. The LC–MS/MS data were both manually inspected to categorize the fragmentation patterns for different classes of metabolites and elaborated using GNPS2 to create informational molecular clusters. Finally, the comparison of the retention time, high-resolution mass and fragmentation pattern with the pure isolated compounds characterized by NMR, was also used to confirm few identifications.

Around one hundred compounds were detected as reported in Table S1; thanks to the high-resolution *m*/*z* values, the chemical formula was easily and accurately defined, disclosing that almost all observed compounds belong to the triterpenoids family, bearing three, four, or five oxygen atoms. Among them, characteristic fragmentations allowed to pinpoint two main classes of triterpenoid acids, namely tirucallanes and α/β-amyrins. As shown in Table 1, ten compounds were undoubtedly identified comparing their retention times and fragmentations with the purified substances (Figure S2), and other thirteen structures (Table S1) were putatively assigned to tirucallanes thanks to their characteristic fragmentation pathway (Figure S3).

**Table 1:** Tirucallanes and α/β-Amyrins Identified by UHPLC-MSMS Analysis

rt	[M – H]^‑^	molecular formula	compound	fragments (relative intensity)
20.01	469.3319	C_30_H_45_O_4_	**7**	355.2643 (35.3) 373.2749 (25.1) 391.3005 (25.9) 407.3319 (100) 409.3112 (13.7) 423.3262 (9.5) 425.3430 (41.2) 451.3210 (14.9)
22.18	455.3527	C_30_H_47_O_3_	**16**	151.1494 (10.6) 339.2685 (100) 359.2916 (6.1) 373.2736 (64.0) 425.3075 (6.0) 437.3418 (50.2) 455.3529 (11.6)
22.85	455.3524	C_30_H_47_O_3_	**14**	121.2632 (9.8) 124.3439 (10.1) 140.1262 (9.8) 140.7101 (10.6) 141.6423 (12.2) 146.9923 (9.6) 151.1129 (16.5) 151.1320 (86.1) 151.1675 (16.9) 162.8083 (11.9) 186.4526 (12.8) 243.5144 (13.7) 359.2913 (13.3) 373.2743 (100) 425.3091 (15.1) 437.3409 (77.6) 455.3520 (17.7)
23.26	453.3371	C_30_H_45_O_3_	**17**	134.9516 (11.0) 151.0954 (9.1) 151.1166 (13.2) 151.1477 (66.4) 151.1629 (14.7) 207.1268 (12.5) 339.2683 (100) 371.2581 (96.1) 391.3335 (28.9) 397.3106 (53.0) 407.3178 (14.4) 435.3258 (77.7) 453.3378 (15.8)
23.34	455.3516	C_30_H_47_O_3_	**15**	129.6152 (29.6) 143.7053 (38.1) 147.5337 (36.3) 151.1510 (100) 341.2812 (56.7) 373.2701 (86.0) 437.3381 (68.6)
23.96	453.3374	C_30_H_45_O_3_	**8**	375.3047 (11.1) 391.3361 (25.7) 397.3103 (100.0) 407.3310 (8.0)
24.43	455.3528	C_30_H_47_O_3_	**1**	375.3051 (10.6) 377.3195 (5.4) 407.332 (9.0) 409.3465 (95.2) 409.906 (5.8) 437.3417 (100)
24.79	455.3528	C_30_H_47_O_3_	**2**	361.2878 (5.2) 377.3206 (27.7) 391.3005 (13.3) 407.3329 (10.1) 409.3470 (86.5) 427.3203 (5.4) 437.3423 (100)
28.56	497.3633	C_32_H_49_O_4_	**3**	447.8221 (23.74) 450.8129 (22.82) 450.8741 (5.38) 451.8687 (24.98) 453.8536 (12.50) 461.818 (7.10) 478.7694 (8.28) 479.8264 (100)
29.93	497.3636	C_32_H_49_O_4_	**4**	447.8209 (23.52) 450.7491 (5.01) 450.815 (20.41) 451.8683 (17.82) 453.8526 (7.90) 461.8191 (5.68) 478.7667 (7.44) 479.8252 (100)

Indeed, tirucallic acid derivatives displayed the diagnostic fragmentation of the side chain; their McLafferty rearrangement led to a characteristic 82 u.m.a loss and a less frequent 114 u.m.a loss, imputable to the subsequent loss of one molecule of oxygen (Figure S3). Moreover, the loss of 142 u.m.a was detected and was ascribed to the McLafferty rearrangement followed by decarboxylation by losing acetic acid.

Beyond tirucallic acid derivatives, also boswellic acids, the most abundant amyrin derivatives of this extract, showed a characteristic fragmentation, responsible for the loss of 78 u.m.a. Indeed, this loss is due to the carboxylic acid in position 4 that generates a rearrangement that finally led to the opening of ring A (Figure S4).

To have a snapshot of the triterpene composition of BhE, also feature-based molecular networking (MN) was performed. MZmine was used to translate the raw data into a feature list that associates fragments to MS1 spectra, and the results obtained were used to generate the feature-based MN to cluster together compounds with similar fragmentation patterns. A rather high cosine score, with a value of 0.7, was chosen to separately cluster different classes of triterpenes, and the network was further revised to eliminate redundant compounds that derived from the formation of ionic adducts. Furthermore, the in-source formation of dimers and trimers was considered, therefore the corresponding nodes were eliminated from the network.

As reported in Figure 6, MN suggests the presence of many tetracyclic and pentacyclic triterpenes such as poli-hydroxylated and unsaturated species. More in details, many tirucallic acid derivatives, such as hydroxy, dihydroxy, trihydroxy, hydroxy-dehydro, and didehydro derivatives, were disclosed as well as 3-hydroxy-tirucallic acid isomers and several boswellic acid derivatives, such as keto, hydroxy, dihydroxy, and dehydro ones.

**Figure 6:**
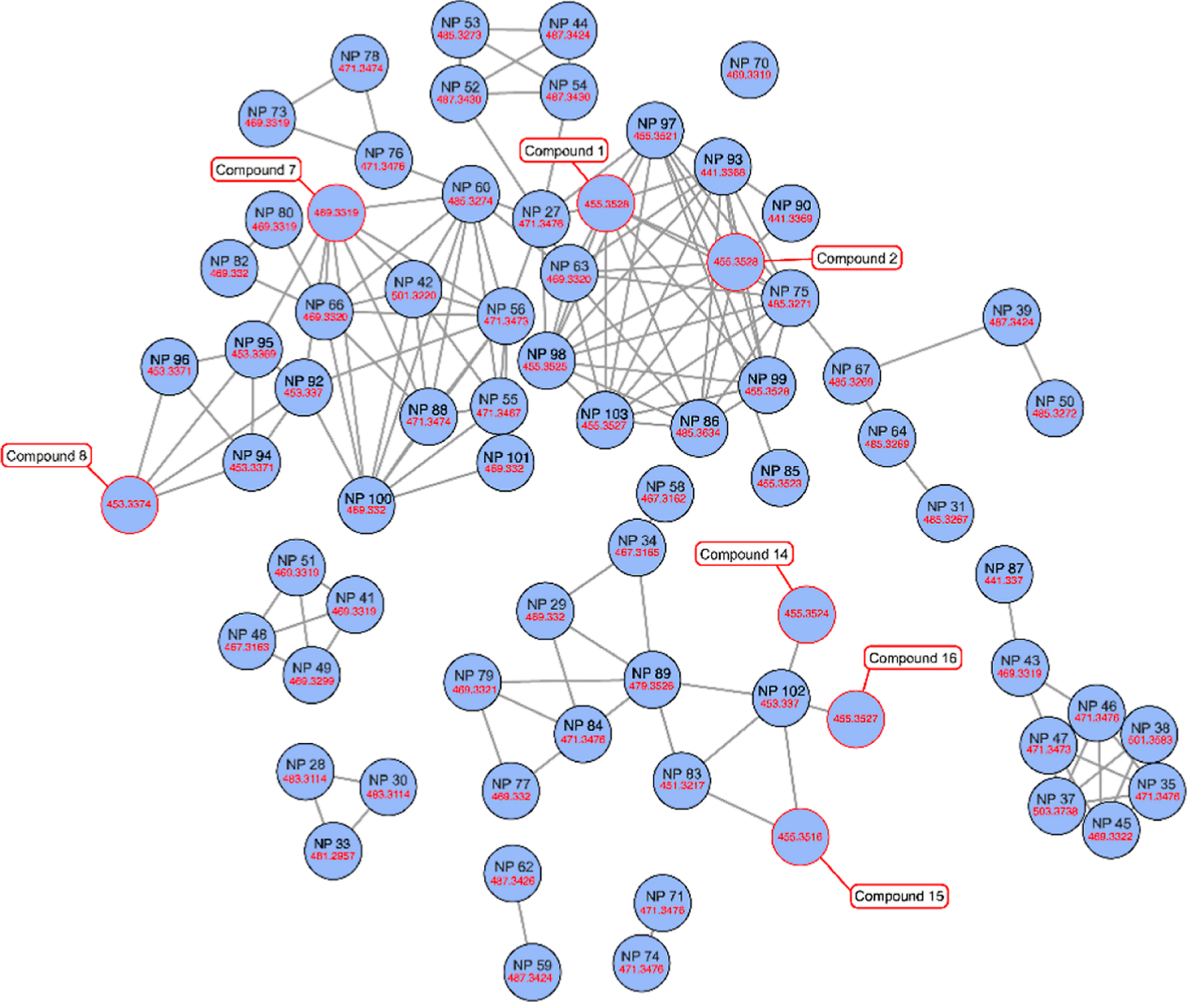
MN of *Boswellia* triterpenoids. Red boxes show some isolated compounds of Table 1; blue circles show compounds of Table S1 with their numbering.

To the best of our knowledge, this is the first report where the MN approach has been used to guide the phytochemical screening of *Boswellia* extract.

An unambiguous assignment of *Boswellia* metabolites was then achieved upon isolation and NMR spectroscopic characterization, using mono and bidimensional experiments (see Supporting Information) and comparison with published data of the obtained compounds. With this aim, BhE was subjected to medium-pressure liquid chromatography followed by HPLC purifications and spectroscopic analysis. Our studies led to the identification of 20 triterpenoids. The main metabolites isolated were known pentacyclic triterpene acids such as α-boswellic acid (**1**), β-boswellic acid (**2**), and their 3-*O*-acetyl derivatives (**3** and **4**) together with 11-keto-β-boswellic acid (**7**) and 9,11-dehydro-β-boswellic acid (**8**), already identified by MN. Besides the boswellic acids, the purification step afforded also four tetracyclic tirucallic acids (**14–17**), differing each other in terms of stereochemistry at the hydroxyl group or keto moiety at the C3 position and for the position of the cyclic double bond located at either position 7 or 8. In addition to the acidic compounds annotated by MN accounting for about 65% of the extract, the *n*-hexane extract of *Boswellia* contained additional neutral triterpenes: α- and β-amyrines (**9**, **11**) and their 3-α-OH epimers (**10** and **12**), 3-epilupeol (**18**), lupeol (**19**), and **13**, identified only after their isolation. The chromatographic purification afforded also the 24-hydroxy derivatives of lupeol, epi-α-amyrine, and epi-β-amyrine, compounds **20**, **5**, and **6**, respectively. Compound **20**, to the best of our knowledge, is described here for the first time as a novel lupane derivative. In 2005, lup-20(29)-en-3α,23-diol was reported as a new triterpene from *Glochidion macrophylhm* Benth.^22^ Compound **20** differs from this known compound for the stereochemistry of the hydroxymethyl group at C-4. The comparison of the ^1^H NMR spectra of **20** with the data reported in literature for lup-20(29)-en-3α,23-diol immediately highlighted some differences in the chemical shift of protons in rings A and B of the lupane nucleus (Table S2). We observed a downfield shift of CH_3_ at C-4 (δ_H_ 1.09 in **20** vs 0.69) and of H-3β (δ_H_ 3.87 in **20** vs 3.67) attributable to a different stereochemistry of the hydroxymethyl group at C-4. Rotating-frame Overhauser Enhancement Spectroscopy (ROESY) correlation peaks between H_2_-24/H_3_-25, H-3β/H_3_-25, and H_3_-23/H-5α unambiguously confirm the β-orientation of the hydroxymethyl group (Figure 7).

**Figure 7:**
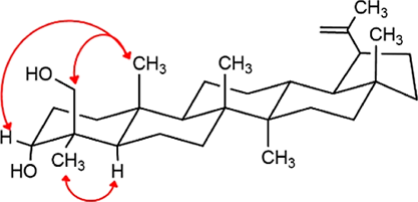
ROESY correlations of compound **20**.

As regards compound **6**, it has been reported in literature as a semisynthetic derivative of α-boswellic acid, and NMR data are not available. We assigned the chemical structure of this new metabolite by NMR experiments, as reported in Table S2.

In this paper, it was isolated as a natural metabolite, and it could be considered the alcohol intermediate of a series of enzymatic oxidations which convert the C-24 methyl group of 3-*epi*-β-amyrin into the carboxylic group of α-boswellic acid, as reported for compound **5**.^9,23^ To further confirm the β-orientation of the hydroxymethyl group at C-4 we performed a chemical modification of α-boswellic acid. In particular, this compound was subjected to esterification with iodomethane in DMF, followed by reduction with LiBH_4_, affording the synthetic alcohol **6a** whose NMR data were identical to those of natural compound **6** (Scheme 1).

**Scheme 1:**
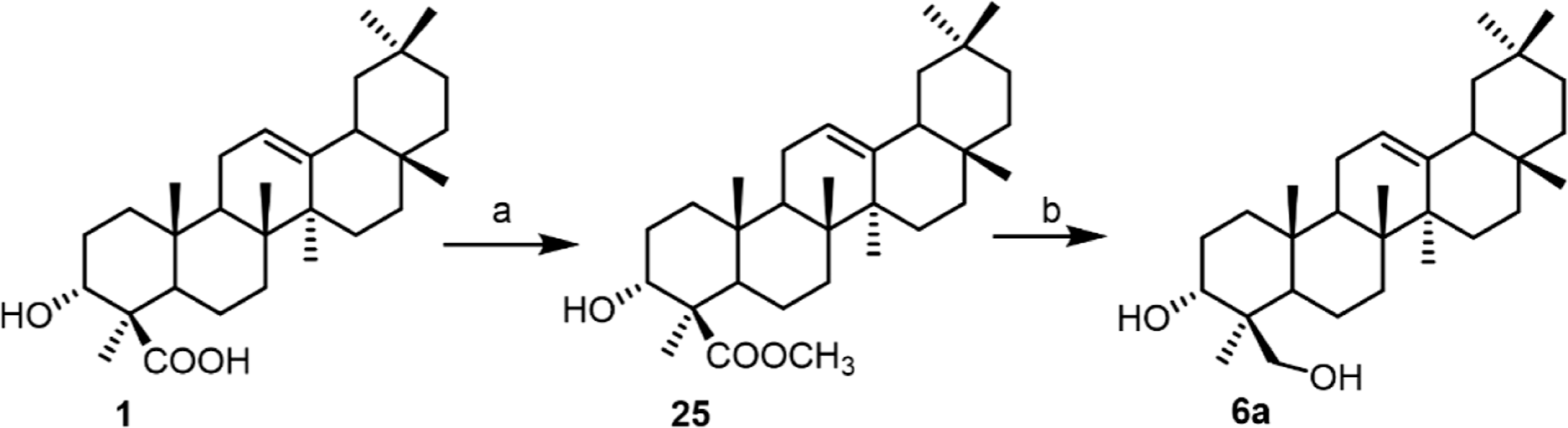
Reagents and Conditions: (a) CH_3_I, K_2_CO_3_, DMF, o.n.; (b) LiBH_4_, MeOH, THF, 0 °C, 2 h

To examine which of the isolated metabolites better modulate the binding between LIF and LIFR, we have settled up a cell free assay based on an Alpha Screen platform. First, the twenty isolated compounds were tested for the efficacy at 10 and 50 μM. Compounds **1–4** and **15–17** showed a very good inhibition percentage at both tested concentrations (Figure 8A). Then, the potency of the most active compounds in inhibiting LIF/LIFR interaction was measured, confirming that the IC_50_ of the above-mentioned compounds was less than 10 μM (Figure 8B); moreover, the IC_50_ values were compared to those of the two well-characterized LIFR antagonists, EC359 and mifepristone,^24^ indicating an even better inhibition profile. This preliminary assay showed that the most active compounds were the ones featuring a carboxylic acid moiety, such as **1–4**, **7**, **8**, **14–17.** Therefore, several of the most effective compounds were also evaluated in a transactivation test on LIFR. As reported in Figure 8C, different compounds confirmed their activity toward this receptor in a concentration-dependent manner.

**Figure 8:**
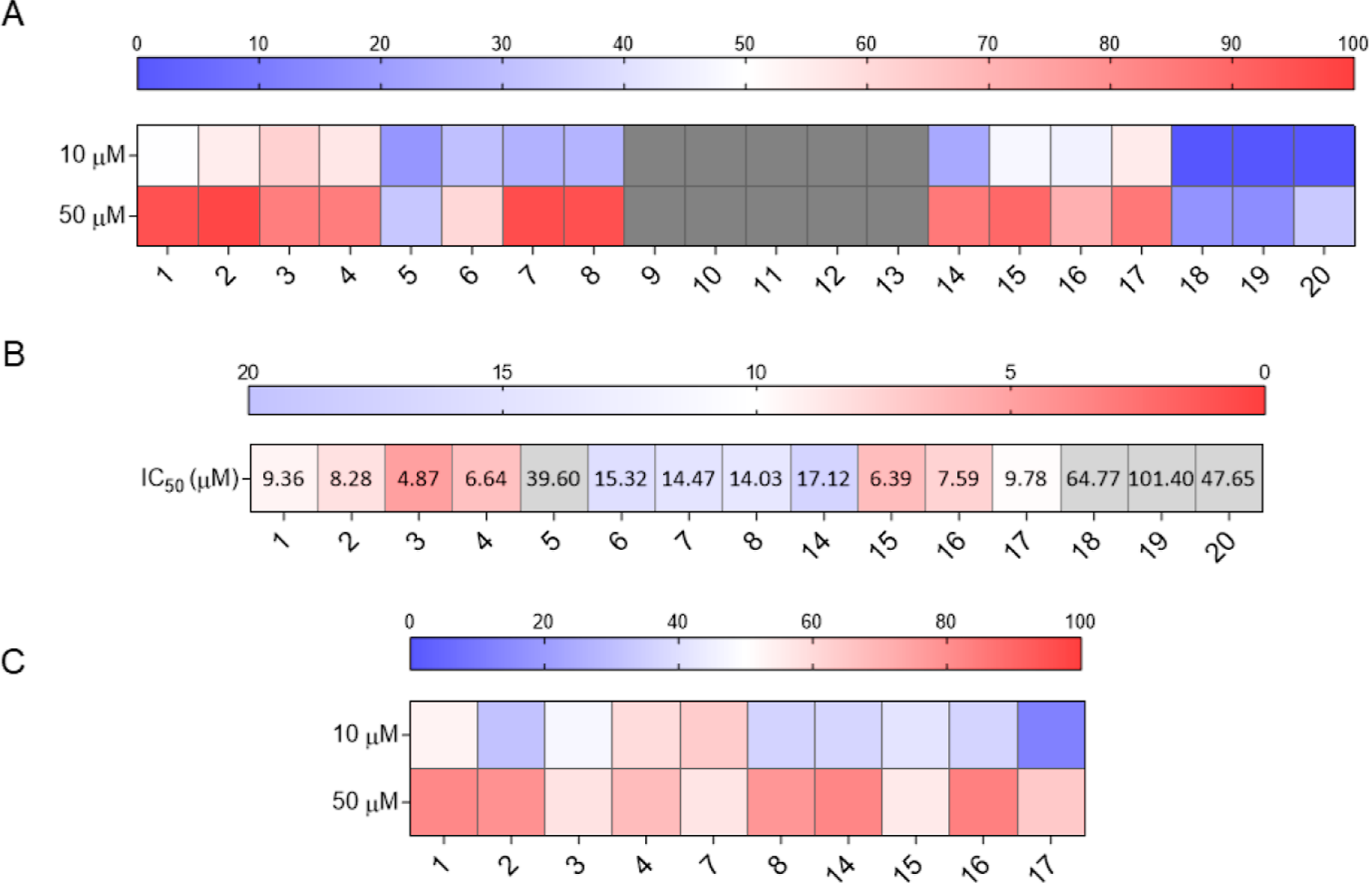
Investigation on isolated compounds activities. (A) LIF-LIFR complex displacement at different compounds’ concentrations (10 and 50 μM), as measured by Alpha Screen. Red squares represent higher % of inhibition, blue ones lower, and gray ones show inactive compounds. (B) Detailed Alpha Screen analysis of the compounds IC_50_. Red squares depict the lowest IC_50_ values, blue squares the highest, and gray ones are intended for compounds whose IC_50_ fell outside the considered range. (C) LIFR transactivation assay in HepG2 cells stimulated with LIF and treated with increasing concentrations (10 and 50 μM) of the analyzed compounds.

A previous work of our research group reported bile acids and their secondary metabolites, mainly glico- and tauro-conjugates, as endogenous antagonists of LIFR.^18^ Thus, combining this information, we decided to evaluate the effect on LIFR antagonism, conjugating the carboxylic moiety of the main compounds α- and β-boswellic acids (**1** and **2**) with taurine or glycine (Scheme 2). For this purpose, the two natural compounds were subjected to conjugation reaction as reported in Scheme 2. Activation of the carboxylic group of compounds **1** and **2** with HBTU gave the stable intermediates **26** and **27**, respectively, which were treated with taurine to obtain **21** and **22**. The glyco-conjugates were obtained following the same coupling reaction using glycine methyl ester. The resulting methyl esters were then hydrolyzed in basic conditions to afford the final metabolites **23** and **24**, respectively.

**Scheme 2:**
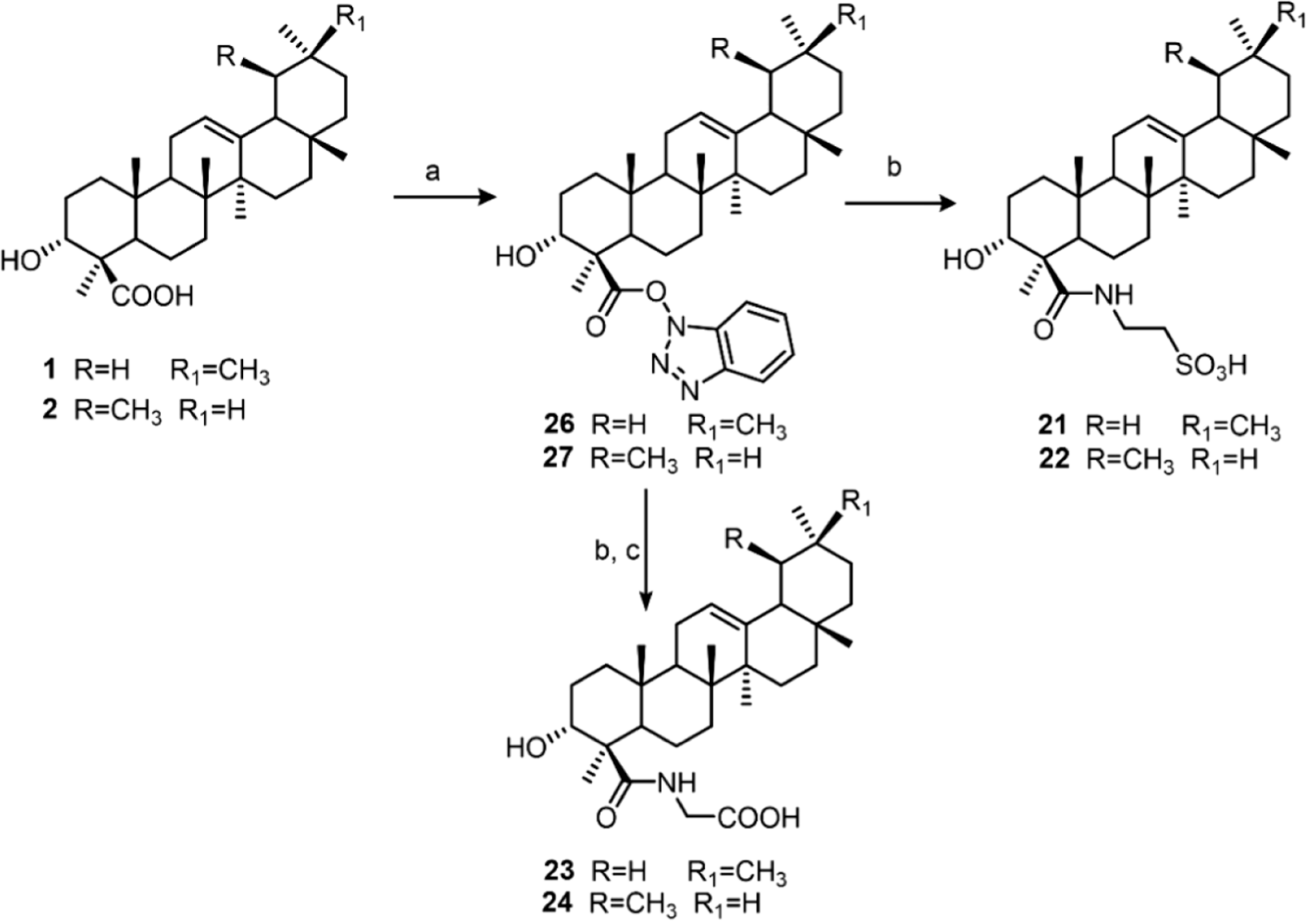
Reagents and Conditions: (a) HBTU, DIPEA in DMF Dry, o.n.; (b) Taurine or Glycine Methyl Ester, *N*,*N*-Diisopropylethylamine, DMF, 4 h; (c) LiOH, THF/H_2_O 1:1, o.n

The conjugate-molecules were tested on LIFR either in Alpha Screen and transactivation assay, showing a moderate activity (Table S4).

To elucidate the binding mode of the most active hLIFR triterpene inhibitors, α-boswellic acid (**1**) and β-boswellic acid (**2**), and disclose the molecular basis of LIF/LIFR inhibition, we performed docking calculations. The hLIFR is characterized by an extracellular region comprising domains D1-D5 including two cytokine-binding modules (CBM1 and CBM2) separated by one Ig-like domain at D3, which mediates the actions of the IL-6 type cytokine LIF (Figure 9).

**Figure 9:**
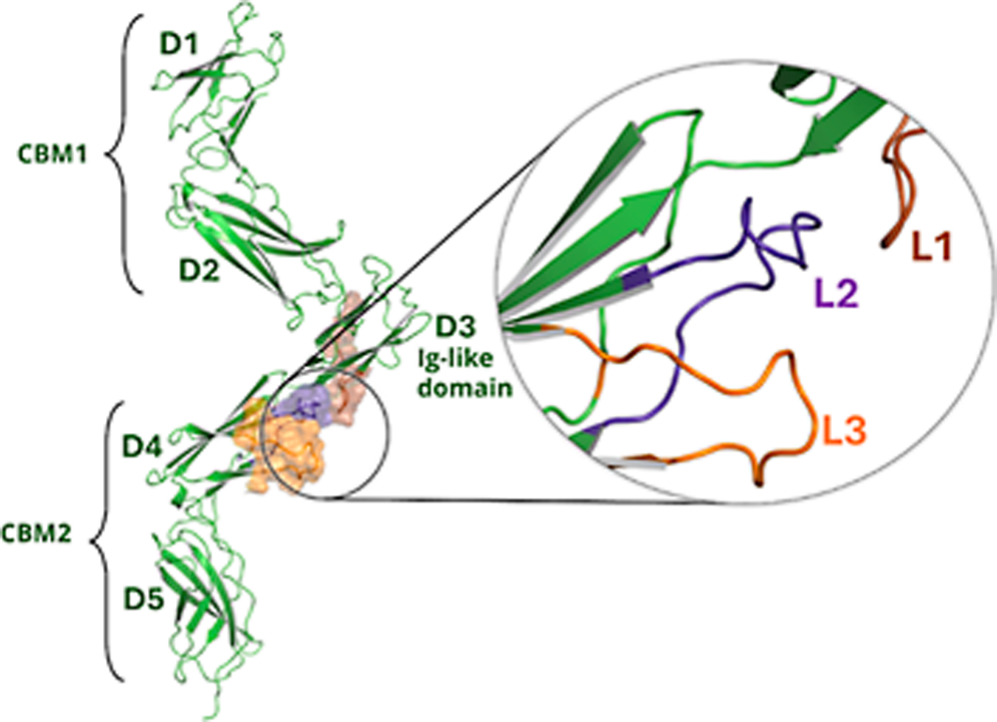
Extracellular segment of hLIFR D1–D5 domains (PDB ID: 3E0G) used for docking calculations with a definition of the cytokine binding (CBD) module and the ligand binding site defined by loop L1 (brown surface/cartoon) of the Ig-like domain D3, and loops L2 (purple surface/cartoon), and L3 (orange surface/cartoon) of the D4 domain.

Beyond the Ig-like domain at D3, LIF binding interfaces also partially with the N-terminal loops of D4 (L3-L2),^25^ which have been previously targeted as putative binding sites to successfully identify small-molecule inhibitors.^24,26,27^ Thus, the most promising compounds **1** and **2** were submitted to molecular modeling studies on L3-L2 loops with the aim of clarifying the role of the carboxyl group in binding to hLIFR.

The QM-Polarized Ligand Docking (QPLD) protocol was applied to compounds **1** and **2**, and the top five poses for each were selected for detailed analysis. For compound **1**, all top-ranked QPLD poses exhibited a conserved binding mode, characterized by the orientation of ring A deeply buried within the receptor binding pocket, engaging one hydrogen bond with Y342 (Figure S5A). Conversely, compound **2** displayed two distinct binding orientations. Indeed, the highest ranked pose positioned ring A toward the solvent-exposed region, with the ursane core stabilized by hydrophobic and van der Waals interactions involving residues V311, A336, V307, P304, and Y342 within loops L2–L3 (Figure S5B). The remaining top-four poses of compound **2** were, instead, similar to those of compound **1**, with the A ring facing inward, enabling the C3 hydroxyl group to form one hydrogen bond with Y342 (Figure S5C). To further evaluate binding energetics, the first-ranked pose of compound **1** and the two top-ranked poses of compound **2** were subjected to MM/GBSA binding free-energy calculations (Table S5), revealing that the Δ*G* of compound **2** was approximately 4 kcal/mol more favorable than that of compound **1**, thus indicating a potentially stronger interaction with the receptor.

In order to incorporate receptor conformational flexibility and allow more accurate accommodation of the triterpene scaffold, the best MM/GBSA poses of compounds **1** and **2** were refined using the Induced-Fit Docking (IFD) protocol. Notably, IFD analysis revealed two distinct and energetically preferred binding orientations, referred as poseA and poseB. In poseA (Figure S6A,B), compounds **1** and **2** exhibited a convergent binding mode, adopting nearly superimposable conformations (Figure S6C) and interacting with key residues within the binding site. Specifically, their hydroxyl group at the C-3 position and carboxyl group at C-4 position directed toward the inner part of the binding pocket engaging interactions with T338 for compound **1** and T388, R333, and E340 for compound **2**. Importantly, significant conformational differences in the triterpene skeleton were observed as a consequence of the distinct pentacyclic scaffolds—oleanane in compound **1** and ursane in compound **2**—highlighting how light variations in the core structure can modulate the molecular recognition within the binding pocket (Figure S6C).

In poseB (Figure S7A,B), we noted an opposite orientation with the triterpene moiety positioned deeper within the binding side, while the OH-3 and COOH-4 groups pointed toward L2 residues engaging in hydrogen bonds with A309, T308, T316, and G305 (Figure S7A,B). Even in such cases, the oleanane and ursane nature of **1** and **2** induced a different conformation of the triterpene moiety (Figure S7C). MM/GBSA rescoring of IFD-refined complexes, poseA and poseB, demonstrated a significant energetic improvement relative to QPLD results, as evidenced by lower binding free-energy (Δ*G*) values and more favorable Emodel scores (Table S5) particularly for the most active compound **2**, in line with experimental activity, underscoring the importance of receptor flexibility in accurately capturing the binding behavior of both ligands.

The stability of binding modes A and B was assessed through 500 ns MD simulations (MDs) and analyzing the protein backbone root mean square deviation (RMSD), ligand RMSD (L-RMSD), and Cα root mean square fluctuations (RMSF) along the MD trajectories. From their initial IFD configurations, L-RMSD analysis revealed that compounds **1** and **2** in poseA experienced significant initial deviations (averaging ∼8 Å for compound **1** and ∼6 Å for compound **2**; Figure S8A). Nonetheless, both remained stable over the course of the 500 ns of MD simulation, indicating convergence of the binding modes as confirmed by clustering analysis, which showed that the most populated cluster accounted for 79% and 71% of the trajectory for compounds **1** and **2**, respectively (Figures 10A,B and 11A,B).

**Figure 10:**
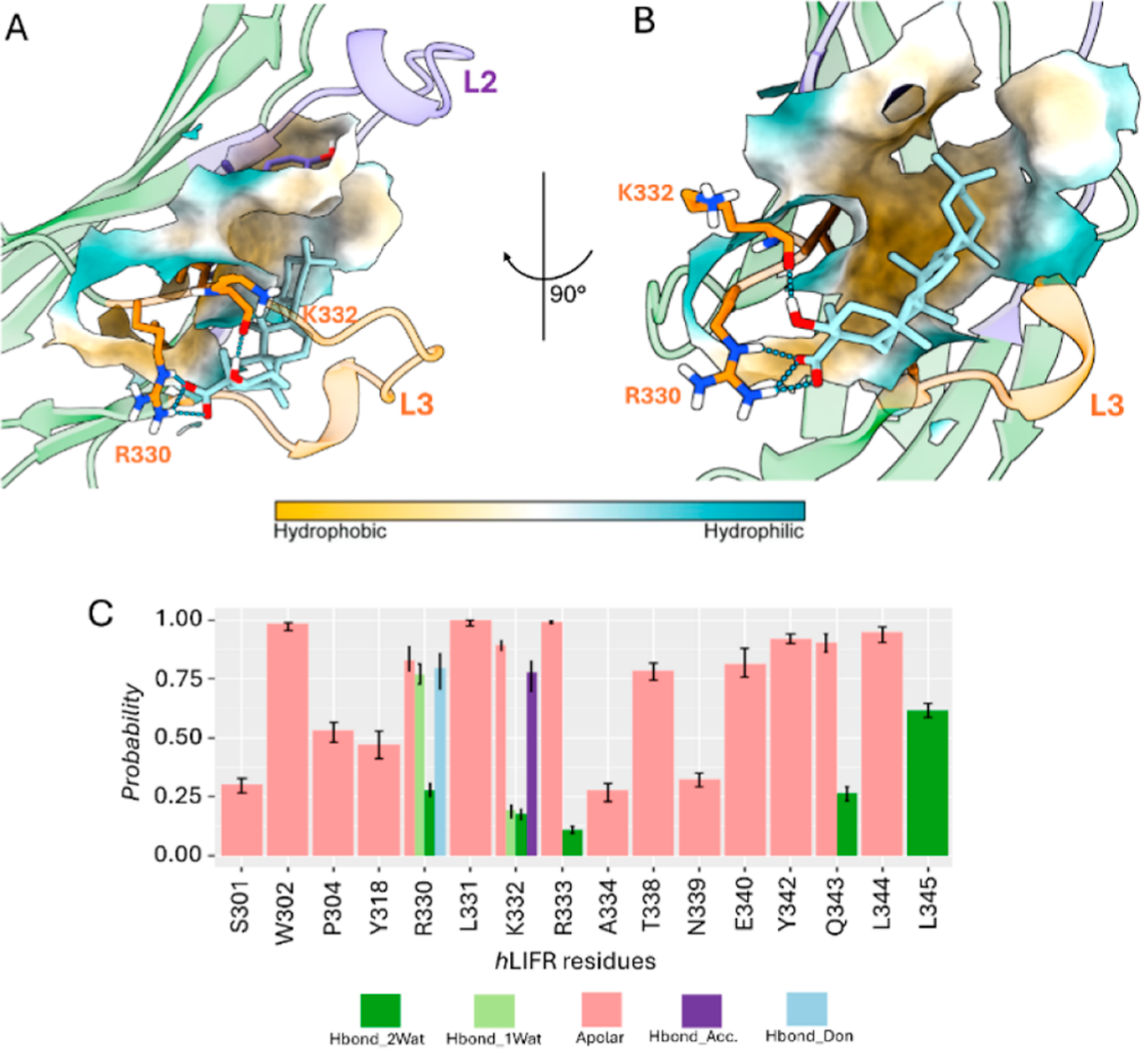
(A,B) Best clustered conformations (cl0) retrieved after 500 ns of MDs of hLIFR/**1** complex obtained from IFD poseA as starting conformation. (C) Ligand–protein Structural Interaction Fingerprint (SIFt) underling the structural interactions hLIFR/**1** complex. Hydrogen bond interactions are displayed as cyan dashed lines.

**Figure 11:**
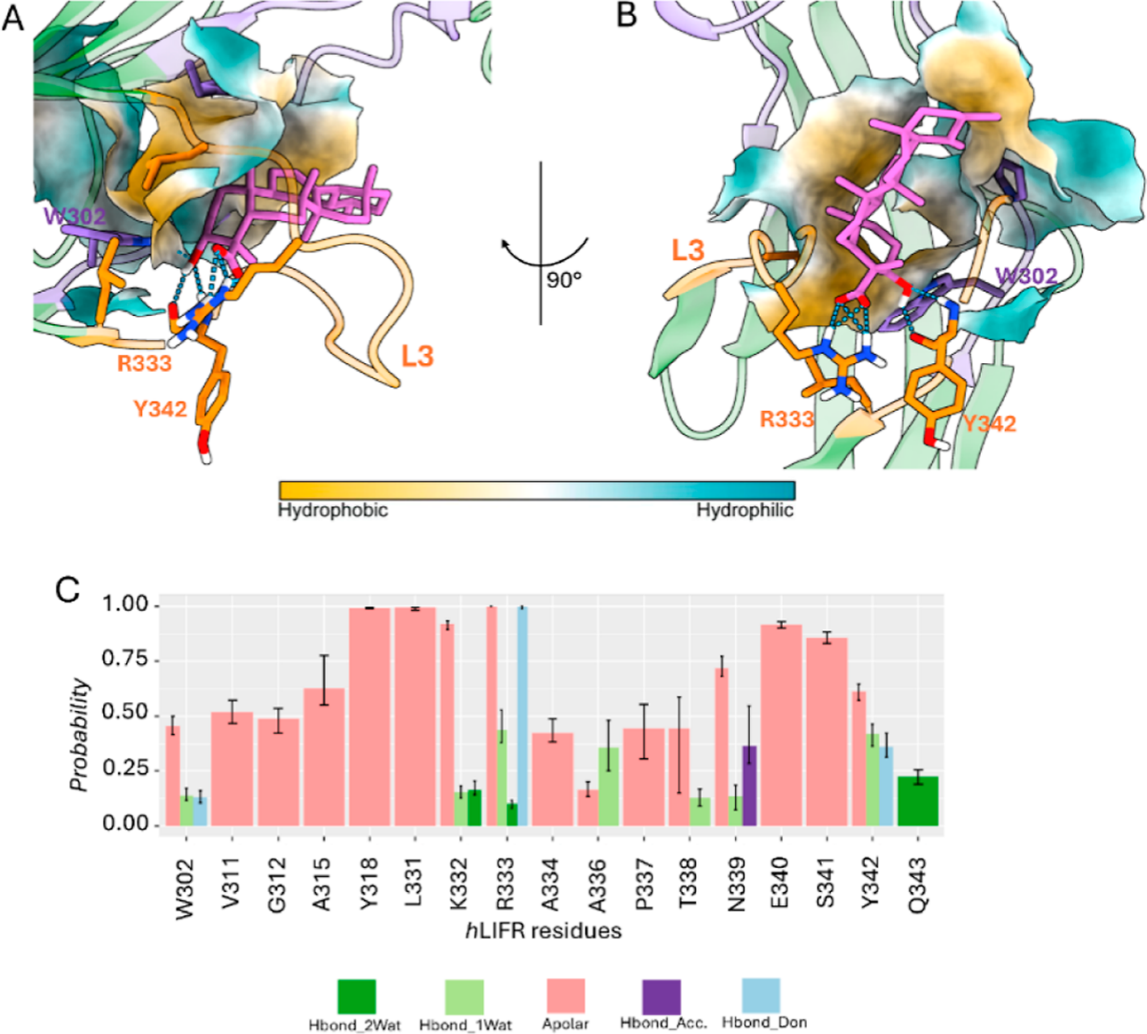
(A,B) Best clustered conformations (cl0) retrieved after 500 ns of MDs of hLIFR/**2** complex obtained from IFD poseA as starting conformation. (C) Ligand–protein Structural Interaction Fingerprint (SIFt) underling the structural interactions hLIFR/**2** complex. Hydrogen bond interactions are displayed as cyan dashed lines.

In contrast, L-RMSD of poseB displayed lower overall deviations but higher fluctuations, suggesting an unstable binding mode (Figure S8B). From hLIFR RMSD and RMSF is evident how the global dynamic behavior of the receptor is strongly influenced by the bound pose (Figure S8C–F). RMSF was used to perform a comparative analysis of the active site residue mobility across the two complexes. Notably, poseA increased fluctuations in key flexible regions, particularly the L1 and L3 loops (Figure S8E,F), potentially impairing LIF binding by destabilizing the receptor conformation.

The structural determinants of protein/ligand interactions were explored through Structural Interaction Fingerprint (SIFt) analysis completed with the hydrogen bond occupancy profiling throughout the simulations (Figures S9 and S10). Consistent with the largely hydrophobic nature of the inner binding pocket, comprising residues W302, P304, L331, and L344, the SIFt plot evidenced hydrophobic interactions as major contributors to ligand binding in both poses (Figures 10–13C), as displayed by protein surface representations (Figures 10–13A,B). However, both the hydrogen bond donor (−OH at C-3) and acceptor (−COO^–^ at C-4) groups are strongly involved. In particular, electrostatic interactions and persistent hydrogen bonds were more pronounced in poseA. Indeed, for both compounds, the –COO^–^ group at C-4 established long-lived salt bridges with the guanidinium side chains of R330 and R333 (Figure S9A–C), while the –OH group at C-3 formed stable hydrogen bonds with the backbone of K332 (hLIFR/**1**) and Y342 (hLIFR/**2**) (Figure S9B–D). In hLIFR/**2**, additional contacts with N339 and W302 were observed, though they were transient and were present during a limited portion of the MD trajectory.

**12:**
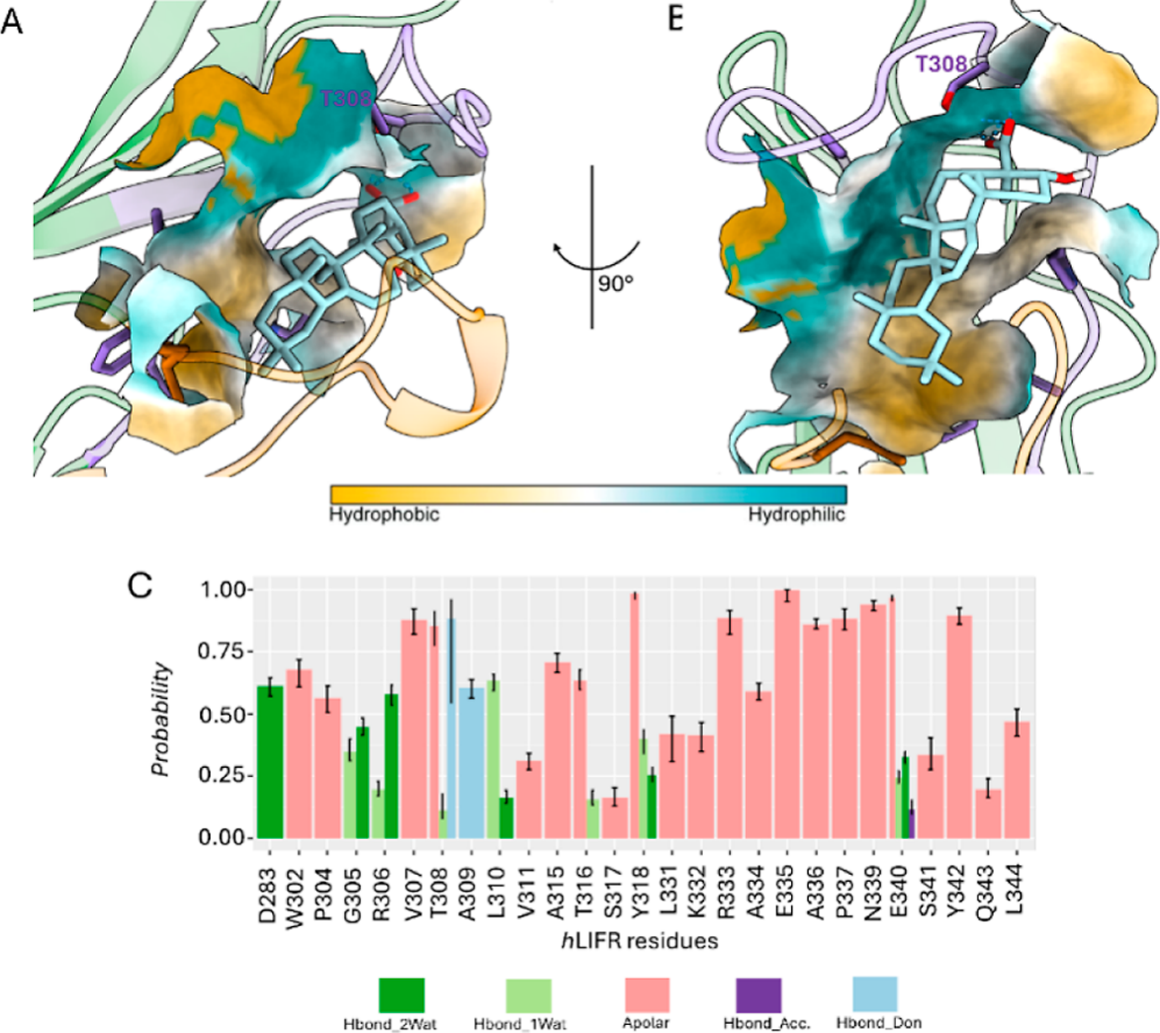
(A,B) Best clustered conformations (cl0) retrieved after 500 ns of MDs of hLIFR/**1** complex obtained from IFD poseB as starting conformation. (C) Ligand–protein Structural Interaction Fingerprint (SIFt) underlining the structural interactions hLIFR/**1** complex. Hydrogen bond interactions are displayed as cyan dashed lines.

**Figure 13:**
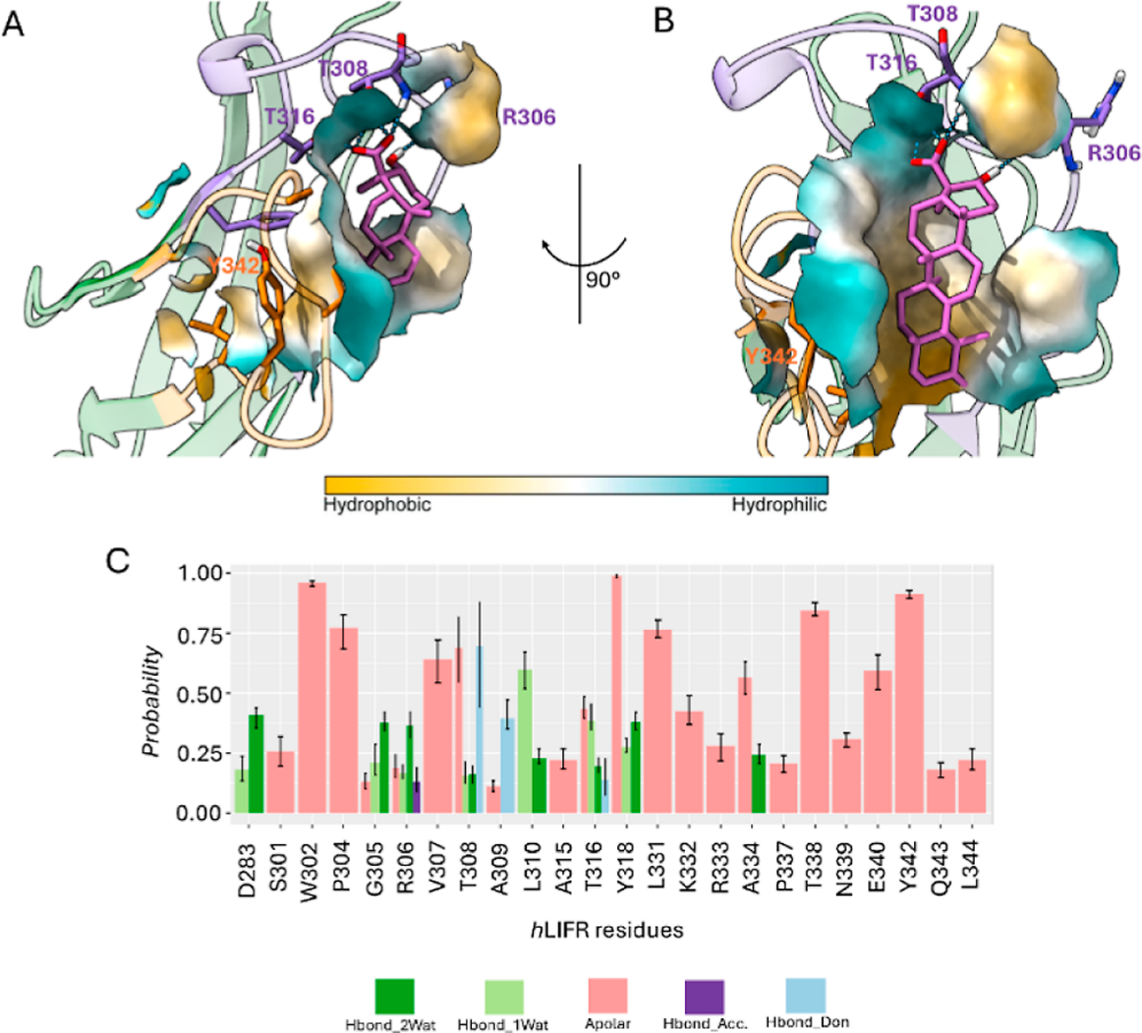
(A,B) Best clustered conformations (cl0) retrieved after 500 ns of MDs of hLIFR/**2** complex obtained from IFD poseB as starting conformation. (C) Ligand–protein Structural Interaction Fingerprint (SIFt) underlining the structural interactions hLIFR/**2** complex. Hydrogen bond interactions are displayed as cyan dashed lines.

In contrast, poseB was characterized by a lack of electrostatic interactions and a reduced hydrogen bonding stability (Figure S10), particularly with the side chain of T308 and the backbone of A309. In poseB, SIFt analysis revealed a greater occurrence of water-mediated hydrogen bonds, consistent with an increased ligand solvent-accessible surface area (L-SASA) (Figure S11B), that instead was significantly lower in poseA (Figure S11A), indicating a more deeply buried and stabilized binding mode. This difference aligns with the higher conformational mobility of the L3 loop observed in poseB complexes (Figure S12B). Overall, the MD results clearly support the structure–activity relationships, indicating that the carboxyl group at C-4 is essential for the activity of both **1** and **2** as engaged persistent electrostatic interactions and hydrogen bonds with a positively charged residues R330 and R333, respectively as well as the hydroxyl group at C-3 is essential to engage hydrogen bond with the backbone atoms mostly of K332 and Y342. Such findings are consistent with the hypothesis that poseA is the binding mode that better explains the molecular basis of the hLIFR inhibitory activity since it induced a higher destabilization of L1 and L3 loops, key regions responsible with the binding of LIF. However, despite the evidence depicting poseA as the most reliable binding mode, it is worth mentioning that the intrinsic flexibility of the L1–L3 loops in hLIFR may impact ligand accommodation and stability within the binding site, representing a potential limitation for accurately resolving the binding mode through dynamic analysis (Figure S13A,B).

To evaluate the activity of the most bioactive metabolites **1** and **2** on a hepatic antifibrotic cellular model, these compounds were tested on human hepatic stellate cell lines (LX-2) treated with TGF-β. The stellate cells play a key role in human liver fibrosis since they are activated into fibrogenic myofibroblast-like cells under several pathological conditions, such as fatty liver disease, obesity, and diabetes.^28^ As shown in Figure 14, the activation of LX-2 with TGF-β, resulted in a significant increase in the expression of Tgf-β, α-Sma, and Col1α1 genes that are indicative of stellate cell activation and transition into a myofibroblastic phenotype. The results revealed that compounds **1** and **2** at the concentration of 10 μM reduced the expression of the two biomarkers Col1α1 and α-Sma (Figure 14A–C), indicating decreased cell activation and collagen deposition. Moreover, they showed a good activity in terms of reduction of amounts of the cytokine TGF-β, also when compared to 6-ECDCA, a known antifibrotic agent (Figure S14).^29^

**Figure 14:**
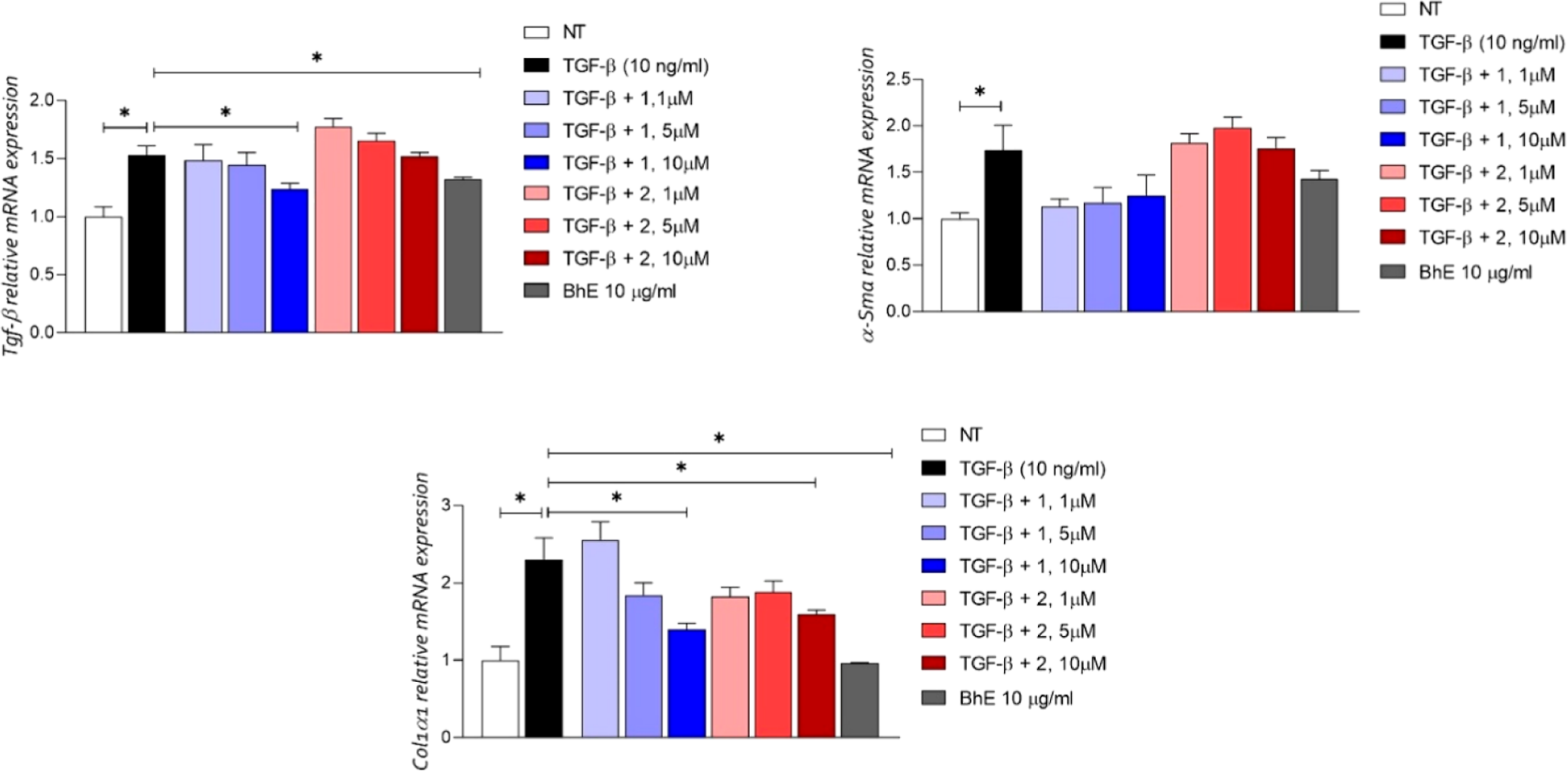
LX-2 cells were activated with TGF-β (10 ng/mL) and exposed to compound **1** or **2** at the concentrations of 1, 5, and 10 μM for 24 h. Quantitative real-time PCR analysis of expression of genes correlated to the activation of stellate cells Tgf-β, α-Sma, and Col1α1. Data are normalized to GAPDH. Results represent mean ± SEM, *p < 0.05 vs TGF-β group.

## Conclusions

In the present study, we have better deciphered the composition of B. serrata *n*-hexane extract through an untargeted metabolomic analysis using high-resolution mass spectrometry and molecular networking. The subsequent isolation of 20 secondary metabolites and pharmacological evaluation led to the identification of α- and β-boswellic acids (**1** and **2**) as new LIFR antagonists. Overall, molecular modeling studies identified the key motifs underlying the interactions of compounds **1** and **2** with the LIFR receptor. The polar region, consisting of the carboxyl group at the C-4 position and the hydroxyl group at the C-3 position, established several electrostatic and hydrogen bonding acceptor and donor interactions with the L3 or L2 loop residues in pose A or B, respectively. However, the hydrophobic interactions of the triterpene moiety were not negligible and occurred in over 75% of the cases, especially for compound **1**. In our study, the activity of compounds **1** and **2** as LIFR antagonists was confirmed by *in vitro* evaluation. The two compounds indeed showed to reduce hepatic stellate cells activation and collagen deposition, highlighting their antifibrotic action and the potential role of these bioactive metabolites of *Boswellia* extracts on liver fibrosis therapy.

In conclusion, through combined metabolomic, pharmacological, and molecular modeling approaches, we elucidated the key structural features, particularly polar and hydrophobic interactions, underpinning the binding of compounds **1** and **2** to the LIFR receptor. These findings not only enhance our understanding of the molecular basis of boswellic acids activity but also support their potential as lead compounds for the development of new therapies targeting liver fibrosis.

## Experimental Section

### General Experimental Procedures

NMR spectra were acquired on a Bruker Avance NEO 400 spectrometer with a RT-DR-BF/1H-5 mm-OZ SmartProbe (^1^H at 400 and ^13^C at 100 MHz). Coupling constants (*J* values) are reported in Hertz (Hz), and chemical shifts (δ) in ppm, referred to CDCl_3_ (δ_H_ 7.26 and δ_C_ 77.0). Spin multiplicities are given as s (singlet), br s (broad singlet), d (doublet), t (triplet), or m (multiplet).

High-resolution electrospray ionization mass spectrometry (ESI-MS) spectra were performed with an LTQ-XL equipped with an Ultimate 3000 HPLC system (Thermo Fisher Scientific) mass spectrometer. ^13^C–^1^H connectivities were evidenced using heteronuclear single quantum coherence (HSQC) and heteronuclear multiple bond correlation (HMBC) experiments, while ^1^H–^1^H connectivities were determined through correlated spectroscopy (COSY) experiment.

A Biotage Selekt System equipped with ready-packed Agela silica columns (40 g) was used for flash chromatography. Solvents used for extraction and purification were supplied from Exacta Optech. Purification was monitored via thin layer chromatography (TLC) on Alugram silica gel G/UV254 plates. HPLC was performed with a Waters model 510 pump equipped with Waters Rheodine injector and a differential refractometer, model 401. HPLC analysis was used also to determine the purity of compounds (>95%).

### Plant Material

B. serrata plant, provided from Farmaceutici Procemsa S.p.A. as hydroalcoholic dry extract, was directly dissolved in methanol. This variety is characterized by a very high content of boswellic acids (65%).

### Extraction and Isolation

Dry extract of B. serrata (50 g) was dissolved in methanol (500 mL), and the crude methanolic extract was subjected to a modified Kupchan’s partitioning procedure^30^ to obtain three extracts: *n*-hexane (15 g), CHCl_3_ (22 g), and *n*-BuOH (1.2 g) and an aqueous residue. Purification of 3 g of *n*-hexane and chloroform extract using a Biotage Selekt System, equipped with a ready-packed Agela silica column (40 g), using a gradient of hexane and ethyl acetate as eluent, starting from 100% hexane up to 8:2, 7:3, 4:6, 1:1 hexane/ethyl acetate, and 100% ethyl acetate, provided fourteen and fifteen fractions, respectively, which have shown to contain the same components at NMR.

HPLC purification was performed on a Synergi Hydro C18 column (4 μm, 4.6 mm i.d × 250 mm) using a different mixture of MeOH/H_2_O as eluent (flow rate 1.0 mL/min) and led to the isolation of twenty components, including eighteen known triterpenoids (**1–5** and **7–19**) identified as α-boswellic acid (**1**), β-boswellic acid (**2**), 3-*O*-acetyl-α-boswellic acid (**3**), 3-*O*-acetyl-β-boswellic acid (**4**), 3α,24-dihydroxy-urs-12-en (**5**), 11-keto-β-boswellic acid (**7**), 9,11-dehydro-β-boswellic acid (**8**), α-amyrine (**9**), epi-α-amyrine (**10**), β-amyrine (**11**), epi-β-amyrine (**12**), tirucalla-7,24-dien-3-α-ol (**13**), 3α-OH-tirucallan-7,24-dien-21-oic acid (**14**), 3α-hydroxy-tirucallan-8,24-dien-21-oic acid (**15**), 3β-OH-tirucallan-8,24-dien-21-oic acid (**16**), 3-ketotirucallan-8,24-dien-21-oic acid (**17**), epilupeol (**18**), and lupeol (**19**) and two undescribed compounds olean-12-en-3α,24-diol (**6**) and lup-20(29)-en-3α,24-diol (**20**).

In particular, fraction 6 of the *n*-hexane extract (67 mg) was purified with MeOH/H_2_O (98:2) as eluent to furnish 1 mg of lupeol (**19**) (*t*_R_ = 24.9 min), 3.5 mg of epilupeol (**18**) (*t*_*R*_ = 29.3 min), 2.6 mg of epi-α-amyrine (**10**) (*t*_*R*_ = 33.5 min), and 12.5 mg of tirucalla-7,24-dien-3-α-ol (**13**) (*t*_*R*_ = 36.8 min).

In the same conditions, fraction 8 (26.4 mg) afforded 17.2 mg of α-amyrine (**9**) (*t*_R_ = 33.5 min) and 0.8 mg of β-amyrine (**11**) (*t*_R_ = 30.6 min), and fractions 11–14 (141.6 mg) were purified using MeOH/H_2_O (92:8) as eluent to furnish 1.2 mg of 11-keto-β-boswellic acid (**7**) (*t*_R_ = 3.9 min), 1.3 mg of 3β-hyroxytirucalla-8,24-dien-21-oic acid (**16**) (t_R_ = 4.9 min), 1.1 mg of 3α-hydroxytirucalla-7,24-dien-21-oic acid (**14**) (*t*_R_ = 5.7 min), 2.7 mg of 3α-hydroxy-tirucalla-8,24-dien-21-oic acid (**15**) (*t*_R_ = 13.9 min), 4.1 mg of 3-oxotirucalla-8,24-dien-21-oic acid (**17**) (*t*_R_ = 19.0 min), 1.8 mg of 9,11-dehydro-β boswellic acid (**8**) (*t*_R_ = 19.7 min), 3.4 mg of α-boswellic acid (**1**) (*t*_R_ = 20.8 min), 12 mg of β-boswellic acid (**2**) (*t*_R_ = 22.4 min), 0.4 mg of lup-20(29)-en-3α,24-diol (**20**) (*t*_R_ = 34.2 min), 1.2 mg of olean-12-en-3α,24-diol (**6**) (*t*_R_ = 43.8 min), and 0.8 mg of urs-12-en-3α,24-diol (**5**) (*t*_R_ = 48.5 min).

Purification of another aliquot of the *n*-hexane fraction (3 g) through Biotage system, in the same conditions described above, furnished twelve fractions and among them HPLC purification of fraction 4, using a Synergi Hydro C18 column (4 μm, 4.6 mm i.d × 250 mm) (flow rate 1.0 mL/min) with MeOH/H_2_O (99:1) as eluent, gave pure compounds 3-*O*-acetyl-α-boswellic acid (**3**) (*t*_R_ = 12.1 min) and 3-*O*-acetyl-β-boswellic acid (**4**) (*t*_R_ = 13.3 min).

Structural elucidations of each compound have been performed by comparing the physical features, NMR spectroscopic data (1D and 2D), and spectrometric analysis data (ESI-MS) with the results previously reported in the literature.

A full NMR assignment led to a complete stereo structural characterization of undescribed compounds **6** and **20** (Figures S15–S21). Notably, compound **20** had been previously mentioned in literature but no NMR data was available.

### Preparation of Compounds **21–24**

Step (a–b): compounds **1** (α-boswellic acid) and **2** (β-boswellic acid) were dissolved in *N*,*N*-dimethylformamide anhydrous (3 mL), and then *N*,*N*-diisopropylethylamine (8 eq) and the coupling reagent HBTU (4 eq) were added. After confirming the formation of the ester intermediate through TLC monitoring, taurine or glycine methyl ester (1.2 eq) and *N*,*N*-diisopropylethylamine (8 eq) were directly added to the reaction mixtures, and the reaction was refluxed overnight. Upon completion, the solvent was removed under vacuum, and the crude residues were extracted with H_2_O/EtOAc (50 mL) three times. The organic layers were dried over Na_2_SO_4_, filtered, and concentrated under reduced pressure to furnish the glycine and taurine methyl ester-conjugated derivatives 21 and 22. Step (c): glycine methyl ester-conjugated intermediates were subjected to alkaline hydrolysis with lithium hydroxide (2 eq) in THF/H_2_O (1:1) at room temperature. Upon completion of the reactions, the mixtures were poured into water and extracted with ethyl acetate (3 × 50 mL). The organic layers were dried over Na_2_SO4, filtered, and concentrated under reduced pressure to yield the glycine-conjugated intermediates **23** and **24**.

### Compounds **21–24**

HPLC purification on Luna C18 column (10 μm, 10 mm i.d. X 250 mm) with MeOH/H_2_O (95:5) + 0.1% trifluoroacetic acid as eluent (flow rate = 3 mL/min) furnished a pure aliquot of derivatives **21** (3 mg, *t*_R_ = 10.0 min); **22** (5 mg, *t*_R_ = 4.5 min); **23** (5 mg, *t*_R_ = 9.41 min); and **24** (4 mg, *t*_R_ = 6.0 min).

#### α-Boswellic Acid or (4R)-3α-Hydroxyolean-12-en-24-oic Acid (**1**)

(CAS no. 471-66-9). Selected ^1^H NMR (CDCl_3_, 400 MHz): δ_H_ 5.19 (t, *J* = 3.4 Hz, 1H, H-12), 4.08 (t, *J* = 2.6 Hz, 1H, H-3β), 1.36 (s, 3H, H_3_-23), 1.15 (s, 3H, H_3_-27), 1.01 (s, 3H, H_3_-26), 0.88 (s, 3H, H_3_-25), 0.87 (s, 6H, H_3_-29 and H_3_-30), 0.83 (s, 3H, H_3_-28); ESI-MS *m*/*z* 455.3 [M – H]^−^.

#### β-Boswellic Acid or (4R)-3α-Hydroxyurs-12-en-24-oic Acid (**2**)

(CAS no. 631-69-6). Selected ^1^H NMR (CDCl_3_, 400 MHz): δ_H_ 5.16 (br t, *J* = 3.3 Hz, 1H, H-12), 4.10 (br t, *J* = 2.5 Hz, 1H, H-3β), 1.35 (s, 3H, H_3_-23), 1.10 (s, 3H, H_3_-27), 1.05 (s, 3H, H_3_-26), 0.92 (ovl, 6H, H3–25 and H_3_-30), 0.81 (s, 3H, H_3_-28), 0.80 (d, *J* = 6.0 Hz, 3H, H_3_-29); ESI-MS *m*/*z* 455.3 [M – H]^−^.

#### 3-*O*-Acetyl-α-boswellic Acid or (4R)-3α-Acetylolean-12-en-24-oic Acid (**3**)

(CAS no. 89913-60-0). Selected ^1^H NMR (CDCl_3_, 400 MHz): δ_H_ 5.20 (br t, *J* = 3.4 Hz, 1H, H-12), 5.32 (br t, *J* = 2.7 Hz, 1H, H-3β), 2.09 (s, 3H, COCH_3_), 1.24 (s, 3H, H_3_-23), 1.19 (s, 3H, H_3_-27), 1.01 (s, 3H, H_3_-26), 0.90 (s, 3H, H_3_-25), 0.87 (ovl, 6H, H_3_-29 and H_3_-30) 0.84 (s, 3H, H_3_-28); ESI-MS *m*/*z* 497.4 [M – H]^−^.

#### 3-*O*-Acetyl-β–boswellic Acid or (4R)-3α-Acetylurs-12-en-24-oic Acid (**4**)

(CAS no. 5968-70-7). Selected ^1^H NMR (CDCl_3_, 400 MHz): δ_H_ 5.15 (br t, *J* = 3.4 Hz, 1H, H-12), 5.31 (br t, *J* = 2.7 Hz, 1H, H-3β), 2.09 (s, 3H, COCH_3_), 1.24 (s, 3H, H_3_-23), 1.12 (s, 3H, H_3_-27), 1.04 (s, 3H, H_3_-26), 0.92 (ovl, 6H, H_3_-25 and H_3_-30), 0.81 (s, 3H, H_3_-28) 0.80 (d, 3H, *J* = 6.2 Hz, H_3_-29); ESI-MS *m*/*z* 497.4 [M −H]^−^.

#### Urs-12-en-3α,24-diol (**5**)

(CAS no. 20475-84-7). Selected ^1^H NMR (CDCl_3_, 400 MHz): δ_H_ 5.15 (br t, *J* = 3.4 Hz, 1H, H-12), 3.90 (br t, *J* = 2.5 Hz, 1H, H-3β), 3.76 (d, *J* = 11.0 Hz, 1H, H-24), 3.57 (d, *J* = 11.0 Hz, 1H, H-24), 1.12 (s, 3H, H_3_-23), 1.11 (s, 3H, H_3_-27), 1.01 (s, 3H, H_3_-26), 0.97 (s, 3H, H_3_-25), 0.95 (d, *J* = 6.7 Hz, 3H, H_3_-30) 0.81 (s, 3H, H_3_-28), 0.80 (d, *J* = 6.0 Hz, 3H, H_3_-29); ESI-MS *m*/*z* 443.4 [M + H]^+^.

#### Olean-12-en-3α,24-diol (**6**)

White amorphous solid; [α]_D_^25^ + 24.9 (*c* 0.14, CH_3_OH); ^1^H and ^13^C NMR data (CDCl_3_, 400 and 100 MHz) reported in Table S2; ESI-MS *m*/*z* 443.4 [M + H]^+^.

#### 11-Keto-β-boswellic Acid or 3-Hydroxy-11-oxo-(3α,4β)-urs-12-en-23-oic Acid (**7**)

(CAS no. 17019-92-0). Selected ^1^H NMR (CDCl_3_, 400 MHz): δ_H_ 5.56 (s, 1H, H-12), 4.10 (br t, *J* = 2.5 Hz, 1H, H-3β), 1.36 (s, 3H, H_3_-23), 1.32 (s, 3H, H_3_-26), 1.20 (s, 3H, H_3_-27), 1.16 (s, 3H, H_3_-25), 0.95 (d, *J* = 6.6 Hz, 3H, H_3_-30), 0.83 (s, 3H, H_3_-28), 0.81 (d, *J* = 6.0 Hz, 3H, H_3_-29); ESI-MS *m*/*z* 469.3 [M – H]^−^.

#### 9,11-Dehydro-β-boswellic Acid or (4R)-3α-Hydroxyurs-9(11),12-dien-24-oic Acid (**8**)

(CAS no. 471-65-8). Selected ^1^H NMR (CDCl_3_, 400 MHz): δ_H_ 5.68 (d, *J* = 5.7 Hz, 1H, H-11), 5.48 (d, *J* = 5.7 Hz, 1H, H-12), 4.11 (br t, *J* = 2.5 Hz, 1H, H-3β), 1.40 (s, 3H, H_3_-23), 1.20 (s, 3H, H_3_-26), 1.14­(s, 3H, H_3_-25), 0.94 (ovl, 6H, H_3_-27 and H_3_-30), 0.87 (s, 3H, H_3_-28), 0.81 (d, *J* = 6.0 Hz, 3H, H_3_-29); ESI-MS *m*/*z* 453.3 [M – H]^−^.

#### α-Amyrine or Urs-12-en-3β-ol (**9**)

(CAS no. 638-95-9). Selected ^1^H NMR (CDCl_3_, 400 MHz): δ_H_ 5.14 (br t, *J* = 3.5 Hz, 1H, H-12), 3.24 (dd, *J* = 10.9, 5.2 Hz, 1H, H-3α), 1.07 (s, 3H, H_3_-27), 1.00 (s, 3H, H_3_-26), 0.99 (s, 3H, H_3_-23), 0.95 (s, 3H, H_3_-25), 0.91 (d, *J* = 6.0 Hz, 3H, H_3_-30), 0.80 (s, 3H, H_3_-28), 0.79 (ovl, 6H, H_3_-24 and H_3_-29); ESI-MS *m*/*z* 427.4 [M + H]^+^.

#### Epi-α-amyrine or Urs-12-en-3α-ol (**10**)

(CAS no. 5937-48-4). Selected ^1^H NMR (CDCl_3_, 400 MHz): δ_H_ 5.19 (br t, *J* = 3.5 Hz, 1H, H-12), 3.23 (dd, *J* = 10.9, 5.2 Hz, 1H, H-3α), 1.15 (s, 3H, H_3_-27), 1.00 (s, 3H, H_3_-23), 0.98 (s, 3H, H_3_-25), 0.95 (s, 3H, H_3_-26), 0.88 (s, 6H, H_3_-29 and H_3_-30), 0.80 (s, 3H, H_3_-24), 0.84 (s, 3H, H_3_-28); ESI-MS *m*/*z* 427.4 [M + H]^+^.

#### β-Amyrine or Olean-12-en-3β-ol (**11**)

(CAS no. 559-70-6). Selected ^1^H NMR (CDCl_3_, 400 MHz): δ_H_ 5.14 (br t, *J* = 3.5 Hz, 1H, H-12), 3.42 (br t, *J* = 2.4 Hz, 1H, H-3β), 1.10 (s, 3H, H_3_-27), 1.02 (s, 3H, H_3_-26), 0.99 (s, 3H, H_3_-25), 0.98 (s, 3H, H_3_-24), 0.93 (d, *J* = 6.0 Hz, 3H, H_3_-30), 0.87 (s, 3H, H_3_-23), 0.81 (ovl, 6H, H_3_-28 and H_3_-29); ESI-MS *m*/*z* 427.4 [M + H]^+^.

#### Epi-β-amyrine or Olean-12-en-3α-ol (**12**)

(CAS no. 6811-63-8). Selected ^1^H NMR (CDCl_3_, 400 MHz): δ_H_ 5.19 (br t, *J* = 3.5 Hz, 1H, H-12), 3.42 (br t, *J* = 2.4 Hz, 1H, H-3β), 1.16 (s, 3H, H_3_-27), 0.98 (s, 3H, H_3_-25), 0.97 (s, 3H, H_3_-24), 0.96 (s, 3H, H_3_-26), 0.88 (s, 6H, H_3_-29 and H_3_-30), 0.86 (s, 3H, H_3_-23), 0.84 (s, 3H, H_3_-28); ESI-MS *m*/*z* 427.4 [M + H]^+^.

#### Tirucalla-7,24-dien-3-α-ol (**13**)

(CAS no. 82570-28-3). Selected ^1^H NMR (CDCl_3_, 400 MHz): δ_H_ 5.26 (br s, 1H, H-7), 5.11 (br t, *J* = 6.7 Hz, 1H, H-24), 3.48 (br t, *J* = 2.6 Hz, 1H, H-3β), 1.69 (s, 3H, H_3_-26), 1.62 (s, 3H, H_3_-27), 0.98 (s, 3H, H_3_-30), 0.95 (s, 3H, H_3_-28), 0.93 (s, 3H, H_3_-29), 0.89 (d, *J* = 7.0 Hz, 3H, H_3_-21), 0.83 (s, 3H, H_3_-18), 0.78 (s, 3H, H_3_-19); ESI-MS *m*/*z* 427.4 [M + H]^+^.

#### 3α-Hydroxytirucalla-7,24-dien-21-oic Acid (**14**)

(CAS no. 82509-40-8). Selected ^1^H NMR (CDCl_3_, 400 MHz): δ_H_ 5.27 (br s, 1H, H-7), 5.10 (br t, *J* = 6.6 Hz, 1H, H-24), 3.48 (br t, *J* = 2.8 Hz, 1H, H-3β), 1.69 (s, 3H, H_3_-26), 1.60 (s, 3H, H_3_-27), 0.98 (s, 3H, H_3_-30), 0.95 (s, 3H, H_3_-28), 0.94 (s, 3H, H_3_-29), 0.91 (s, 3H, H_3_-18), 0.77 (s, 3H, H_3_-19); ESI-MS *m*/*z* 455.3 [M – H]^−^.

#### 3α-Hydroxytirucalla-8,24-dien-21-oic Acid (**15**)

(CAS no. 28282-27-1). Selected ^1^H NMR (CDCl_3_, 400 MHz): δ_H_ 5.10 (br t, *J* = 6.9 Hz, 1H, H-24), 3.45 (br t, *J* = 2.8 Hz, 1H, H-3β), 1.68 (s, 3H, H_3_-26), 1.59 (s, 3H, H_3_-27), 0.97 (s, 3H, H_3_-29), 0.95 (s, 3H, H_3_-18), 0.89 (s, 3H, H_3_-30), 0.87 (s, 3H, H_3_-28), 0.84 (s, 3H, H_3_-19); ESI-MS *m*/*z* 455.3 [M – H]^−^.

#### 3β-Hydroxytirucalla-8,24-dien-21-oic Acid (**16**)

(CAS no. 28282-54-4). Selected ^1^H NMR (CDCl_3_, 400 MHz): δ_H_ 5.10 (br t, *J* = 6.9 Hz, 1H, H-24), 3.24 (dd, *J* = 11.6, 4.5 Hz, 1H, H-3α), 1.68 (s, 3H, H_3_-26), 1.59 (s, 3H, H_3_-27), 1.01 (s, 3H, H_3_-29), 0.95 (s, 3H, H_3_-18), 0.88 (s, 3H, H_3_-30), 0.82 (s, 3H, H_3_-28), 0.80 (s, 3H, H_3_-19); ESI-MS *m*/*z* 455.3 [M – H]^−^.

#### 3-Oxotirucalla-8,24-dien-21-oic Acid (**17**)

(CAS no. 28282-25-9). Selected ^1^H NMR (CDCl_3_, 400 MHz): δ_H_ 5.11 (br t, *J* = 6.9 Hz, 1H, H-24), 1.69 (s, 3H, H_3_-26), 1.60 (s, 3H, H_3_-27), 1.12 (s, 3H, H_3_-29), 1.06 (s, 3H, H_3_-28), 1.05 (s, 3H, H_3_-19), 0.91 (s, 3H, H_3_-30), 0.83 (s, 3H, H_3_-18); ESI-MS *m*/*z* 453.3 [M – H]^−^.

#### Epilupeol or Lup-20(29)-en-3α-ol (**18**)

(CAS No. 4439-99-0). Selected ^1^H NMR (CDCl_3_, 400 MHz): δ_H_ 4.70 (s, 1H, H-29), 4.58 (s, 1H, H-29), 3.39 (br t, *J* = 2.5 Hz, 1H, H-3β), 1.70 (s, 3H, H_3_-30), 1.04 (s, 3H, H_3_-26), 0.98 (s, 3H, H_3_-27), 0.94 (s, 3H, H_3_-24), 0.86 (s, 3H, H_3_-25), 0.85 (s, 3H, H_3_-23), 0.80 (s, 3H, H_3_-28); ESI-MS *m*/*z* 427.4 [M + H]^+^.

#### Lupeol or Lup-20(29)-en-3β-ol (**19**)

(CAS no. 545-47-1). Selected ^1^H NMR (CDCl_3_, 400 MHz): δ_H_ 4.69 (s, 1H, H-29), 4.57 (s, 1H, H-29), 3.19 (dd, *J* = 11.2, 5.0 Hz, 1H, H-3α), 1.69 (s, 3H, H_3_-30), 1.04 (s, 3H, H3–26), 0.97 (s, 3H, H_3_-23), 0.95 (s, 3H, H_3_-27), 0.84 (s, 3H, H_3_-25), 0.80 (s, 3H, H_3_-23), 0.77 (s, 3H, H_3_-28); ESI-MS *m*/*z* 427.4 [M + H]^+^.

#### Lup-20­(29)-en-3α,24-diol (**20**)

White amorphous solid; [α]_D_^25^ + 17.6 (*c* 0.09, CH_3_OH); ^1^H and ^13^C NMR data (CDCl_3_, 400 and 100 MHz) reported in Table S2; ESI-MS *m*/*z* 443.4 [M + H]^+^.

#### Sodium (4*R*)-3α-Hydroxyolean-12-en-24-oyl Taurine (**21**)

White amorphous solid; [α]_D_^25^ + 35.1 (*c* 0.42, CH_3_OH); ^1^H and ^13^C NMR data (CD_3_OD, 400 and 100 MHz) reported in Table S3; ESI-MS *m*/*z* 562.4 [M – H]^−^.

#### (4*R*)-3α-Hydroxyolean-12-en-24-oyl Glycine (**22**)

White amorphous solid; [α]_D_^25^ + 44.1 (*c* 0.07, CH_3_OH); ^1^H and ^13^C NMR data (CD_3_OD, 400 and 100 MHz) reported in Table S3. ESI-MS *m*/*z* 562.4 [M – H]^−^.

#### Sodium (4R)-3α-Hydroxyurs-12-en-24-oyl Taurine (**23**)

White amorphous solid; [α]_D_^25^ + 85.9 (*c* 0.47, CH_3_OH); ^1^H and ^13^C NMR data (CD_3_OD, 400 and 100 MHz) reported in Table S3; ESI-MS *m*/*z* 512.4 [M – H]^−^.

#### (4R)-3α-Hydroxyurs-12-en-24-oyl Glycine (**24**)

White amorphous solid; [α]_D_^25^ + 284.3 (*c* 0.45, CH_3_OH); ^1^H and ^13^C NMR data (CD_3_OD, 400 and 100 MHz) reported in Table S3. ESI-MS *m*/*z* 512.4 [M – H]^−^.

### UPLC-MS/MS Analysis for Chemical Characterization

The *n*-hexane *Boswellia* extract and the isolated pure compounds were analyzed using a high-resolution Tribrid mass spectrometer from ThermoScientific, equipped with an ESI source and a Vanquish Flex UHPLC. One mg/mL solutions of *Boswellia* extract or the isolated pure compounds used as standards at 0.01 mg/mL were injected, and chromatographic separation was achieved using a Phenomenex Luna Omega 3 μm Polar C18 100 Å, 100 × 2.1 mm, LC Column, with H_2_O 0.1% formic acid as eluent A and ACN 0.1% formic acid as eluent B. The gradient, with a 0.4 mL/min flow rate, was as follows: 30% B for 3 min, 30–95% B from 3 to 35 min, 95% B from 35 to 40 min. The column was then re-equilibrated at 30% B for 5 min, with a total chromatographic run of 45 min. Tandem MS analysis was conducted in negative ion mode. Full scan spectra were acquired between 200 and 1500 *m*/*z*, with a resolution of 60000. Data-dependent MS^2^ acquisitions were carried out based on a 0.6 s cycle time, fragmenting the highest number of ions in the given cycle time, with a collision energy % of 35. MS^2^ scans were obtained with a resolution of 15000.

### Global Natural Products Social Molecular Networking Analysis

MS data were elaborated using MZmine, version 3.9.0. MS signals were detected in negative polarity and only signals above a threshold noise level of 5 × 10^6^ for MS^1^ and 2 × 10^4^ for MS^2^ were selected. Only MS^1^ peaks with at least 8 consecutive scans were used to build the feature list. To discard falsely selected features, this list was filtered using the following MZmine modules: Local minimum feature resolver, ^13^C isotope filter, and Duplicate feature list rows filter. Results were then exported as a quantification table (.csv) and a .mgf file, that contains the MS features.

Feature-based molecular networking was ended using GNPS2 (https://gnps2.org). Precursor ion and fragment ion tolerance were both set to 0.02 Da. The network was built using a maximum of 100 nodes, with a cosine score of at least 0.7 and a minimum of 6 matching peaks. The network was visualized using Cytoscape.

### Amplified Luminescent Proximity Homogeneous Assay on FXR-SRC1

A coactivator recruitment assay of the isolated compounds, as well as the full hexane *Boswellia* extract, was performed using an Alpha Screen GST Detection Kit (Revvity, Italy). The assay was performed in 50 mM Tris–HCl (pH 7.4), 20 mM KCl, 1 mM DTT, and 0.1% BSA buffer, using FXR-LBD GST-fused (Thermo Scientific) at a concentration of 10 nM and biotinylated SRC1 peptide (CPSSHSSLTERHKILHRLL-QEGSPS) at a concentration of 30 nM. Both acceptor and donor beads from the detection kit were used at a concentration of 20 μg/mL. Incubations were performed in a 384-well OptiPlate (Revvity, Italy), with a final volume of 25 μL. Alpha signal was measured using a VICTOR Nivo (Revvity, Italy) multimode microplate reader. Each compound was tested at 10 μM and 50 μM, while the hexane extract was assayed at concentration ranging from 12.5 μg/mL to 200 μg/mL.

### Amplified Luminescent Proximity Homogeneous Assay on LIF-LIFR

Recombinant human LIFR (His-Tag) and LIF (biotinylated) were purchased from Sino Biologicals (Sino Biological Europe GmbH, Dusseldorf, Germany) and R&D Systems (Abingdon, UK), respectively, and both were reconstituted as required by the manufacturer. Inhibition of LIFR/LIF binding was measured by Alpha Screen, in white, low-volume, 384-well AlphaPlates (Revvity, Italy) using a final volume of 15 μL and an assay buffer containing 25 mM Hepes (pH 7.4), 100 mM NaCl, and 0.005% Kathon. The concentration of DMSO in each well was maintained at 5% vol/vol. LIFR (His-Tag, final concentration 4.5 nM) was incubated with either the compounds or DMSO for 45 min under continuous shaking. Then, LIF was added (biotinylated, final concentration 9 nM), and the samples were incubated for 15 min prior to adding nickel chelate acceptor beads (final concentration 20 ng/μL) for 30 min. Then, streptavidin donor beads were added (final concentration 20 ng/μL), and the plate was incubated in the dark for 2 h and then read in a Victor Nivo (Revvity, Italy) Multimode Microplate Reader.

### Luciferase Reporter Gene Assay

To evaluate the LIFR/STAT3 signaling, HepG2 (HB, 8065 from ATCC), were seeded at 7.5 × 10^4^ cells/well in a 24-well plate and maintained at 37 °C and 5% CO_2_ in E-MEM added with 10% FBS, 1% glutamine, and 1% penicillin/streptomycin. On day-1, cells were transiently transfected with the reporter plasmid pGL4.47­[luc2P/SIE/Hygro] (CAT no. E4041 Promega, Madison, WI, USA), a vector encoding the hLIFR (CAT no. RC226327) and CD130 (IL6ST) (CAT no. RC215123, OriGene Technologies, Inc. Rockville, MD USA), and finally a vector encoding the human Renilla luciferase gene (pGL4.70) (Promega, Madison, WI, USA). On day-2, cells were primed with the cytokine LIF (10 ng/mL) alone or in combination with different compounds at the concentrations of 10 μM and 50 μM. Then, after 24 h, cellular lysate was assayed for luciferase and Renilla activities using the Firefly & Renilla Luciferase Single TubeAssay Kit (Biotium, Fremont, CA). Luminescence was measured using Glomax 20/20 luminometer (Promega, Madison, WI, USA). Luciferase activities were normalized with Renilla activities.

### Cell Culture

The LX-2 human hepatic stellate cells were maintained in D-MEM enriched with fetal bovine serum (2%), 1% glutamine, and 1% penicillin/streptomycin in a 5% CO_2_ humidified atmosphere at 37 °C. For experimental setting, cells were seeded in 6-well plates; they were activated with TGF-β (10 ng/mL) and then treated with BhE (10 μg/mL), 6-ECDCA (10 μM), and with compounds **1** and **2** at different doses (1, 5, and 10 μM) for 24 h.

### Quantitative Real-Time PCR Analysis

Total RNA was extracted from LX-2 cells using the Direct-zol RNA MiniPrep kit with Zymo-Spin IIC columns (Zymo Research, Irvine, CA). Reverse transcription was carried out with the FastGene Scriptase Basic Kit (Nippon Genetics Europe) in a 20 μL reaction volume. For quantitative real-time PCR (qRT-PCR), 50 ng of cDNA was amplified in a 20 μL reaction mixture containing 200 nM of each primer and 10 μL of PowerUp Master Mix (Thermo Fisher Scientific, Waltham, MA, USA). All reactions were performed in triplicate using the QuantStudio 3 Real-Time PCR System (Applied Biosystems) under the following thermal cycling conditions: an initial denaturation at 95 °C for 10 min, followed by 40 cycles of 95 °C for 10 s, and 60 °C for 45 s. Relative mRNA expression levels were determined using the 2–(ΔΔCt) method, with gene expression normalized to Gapdh mRNA as an internal control. The primers used for real-time PCR were as follows: human-Gapdh: GAAGGTGAAGGTCGGAGT and CATGGGTGGAATCATATTGGAA; human-Tgf-β: CGTCTGCTGAGGCTCAAGTT and AAGATAACCACTCTGGCGAGTC; human-Col1a1: CCCAAGGCTTCCAAGGTC and GACCAGGTTTTCCAGCTTCC; human- α-sma: GTGTTCCCGTCCATCGTG and CTCTTGCTCTGAGCCTCGTC.

### Molecular Docking

The 3D structure of compounds **1** and **2** was generated using the graphical user interface of Maestro ver. 11.8,^31^ and their protonation state at pH 7.4 in water was calculated using the Epik module.^32^ The X-ray structure of hLIFR (PDB ID: 3E0G)^33^ was prepared with the Protein Preparation Wizard to apply structural corrections such as adding missing hydrogen atoms, determine the most likely protonation states of amino acids, and correct residues with missing atoms. The obtained prepared structure was then used for docking studies. Molecular docking on hLIFR was carried out with the QM-polarized ligands docking (QPLD) protocol,^34^ followed by an IFD procedure.^35^ Specifically, the grid box with the default coordinate dimension of 10 Å was centered on the centroid of the hLIFR binding site defined by the L2–L3 loops. Ten docking poses were saved during the QPLD step, and the top-scored of compounds **1** and **2** were submitted to the IFD procedure to investigate also the protein flexibility in the active site. The IFD extended sampling protocol was adopted by generating 80 poses with an energy window of the ligand conformational sampling of 2.5 kcal/mol, as successfully adopted in our previous protocol.^18,20^

### Molecular Dynamics Simulations (MDs)

MDs were performed with the CUDA version of AMBER22 suite^36^ using the ff14SB^37^ and the General Amber Force Field (GAFF2)^38^ parameters for the protein and compounds **1** and **2**, respectively. In particular, ligand charges were calculated by using the restrained electrostatic potential (RESP) fitting procedure. First, the Gaussian16 package was used to calculate the ligand ESP using the 6-31G* basis set at the Hartree–Fock level of theory. The Antechamber and the Leap modules implemented in the AmberTools23 package^39^ allowed generation of ligand topology with the RESP charges coupled with the GAFF2 parameters. Both hLIFR/**1** and hLIFR/**2** complexes were immersed in a pre-equilibrated octahedral box of 10 Å of TIP3P water molecules and then neutralized by adding Na^+^ and Cl^–^ ions. Subsequently, each system underwent a multistep minimization process with an energy gradient convergence criterion set to 0.01 kcal/mol Å^2^. This process involved (i) 5000 minimization steps (2500 using the steepest descent method and 2500 using the conjugate gradient method) involving only hydrogen atoms; (ii) 20,000 minimization steps (10,000 using the steepest descent method and 10,000 using the conjugate gradient method) involving water and hydrogen atoms, while restraining the solute; (iii) 50,000 minimization steps (25,000 using the steepest descent method and 25,000 using the conjugate gradient method) involving only the side chains of the protein, water, and hydrogen atoms; and (iv) 100,000 minimization steps (50,000 using the steepest descent method and 50,000 using the conjugate gradient method) involving a complete minimization. An equilibration protocol of water molecules, ions, and protein side chains occurred in the following steps: (i) 5 ns of *NVT* equilibration with the Langevin thermostat, gradually heating from 0 to 300 K while scaling down solute restraints from a force constant of 10 to 1 kcal/mol Å^2^; (ii) 5 ns of *NPT* equilibration at 1 atm with the Berendsen thermostat, gradually scaling down restraints from 1.0 to 0.1 kcal/mol Å^2^; and (iii) 5 ns of *NPT* equilibration with no restraints. Finally, a MD production run of 500 ns in *NPT* ensemble was conducted for each system using a time step of 2 fs. The SHAKE algorithm was employed for bonds containing hydrogen atoms, in conjunction with periodic boundary conditions. Long-range electrostatic interactions were treated using the Particle Mesh Ewald method with a cutoff of 10 Å for nonbonded interactions. MD trajectories were visualized using VMD ver. 1.9.3 software,^40^ while clustering and analysis procedures were performed through the CPPTRAJ module.^41^ For the most representative cluster population, intermolecular interaction energies were analyzed via the molecular mechanics/generalized born surface area (MM/GBSA) equation using the MMPBSA.py script of the AMBER22 package. Ligand–protein Structural Interaction Fingerprint (SIFt) was calculated using the algorithm described by Deng and co-workers.^42^

All figures were rendered using PyMOL ver. 2.5.0 (www.pymol.org/pymol) and UCSF Chimera.

## Supplementary Material



## Notes

UHPLC-MSMS analysis, NMR data of compounds **6**, **20**–**24**, assays on LIFR of synthetic compounds **21**–**24**, docking calculations, LC–MS traces of BhE, MS fragmentations of boswellic and tirucallic acid derivatives, MD simulations, PCR analysis, and NMR spectra of compounds **6**, **20–24** (PDF)

## Abbreviations

AKBA: acetyl-11-keto-β-boswellic acid
Akt: protein kinase B
BhE: *Boswellia* *n*-hexane extract
CBM: cytokine-binding modules
DIPEA: *N*,*N*-diisopropylethylamine
FXR: farnesoid X receptor
GPBAR1: G protein-coupled bile acid receptor 1
HBTU: hexafluorophosphate benzotriazole tetramethyl uranium
IBD: inflammatory bowel diseases
IFD: induced fit docking
JAK/STAT: Janus kinase/signal transducer and activator of transcription
LIF: leukemia inhibitory factor
LIFR: leukemia inhibitory factor receptor
LC–MS/MS: liquid chromatography–mass spectrometry/mass spectrometry
L-RMSD: root-mean-square deviation of the ligands
LX-2: human hepatic stellate cell line
5-LOX: 5-lipoxygenase
MAPK: mitogen-activated protein kinase
MD: molecular dynamics
MM/GBSA: molecular mechanics/generalized born surface area
MN: molecular networking
NMR: nuclear magnetic resonance
mPGES-1: microsomal prostaglandin E synthase-1
RMSF: root mean square fluctuations
L-SASA: solvent accesible surface area of the ligands
SIFt: structural interaction fingerprint
α-SMA: alpha-smooth muscle actin
mTOR: mechanistic target of rapamycin
TGF-β: transforming growth factor-β
UHPLC–MS/MS: ultrahigh-performance liquid chromatography–mass spectrometry/mass spectrometry
