# Supplementary material for: Exploring Boswellia serrata Triterpenes: A New Frontier in Leukemia Inhibitory Factor Receptor Modulation
Source: ACS Omega. 2025 May 21;10(21):22269–84. doi: 10.1021/acsomega.5c03492 (PMC12138715; doi:10.1021/acsomega.5c03492)

Supporting Information

Exploring *Boswellia serrata* Triterpenes: A New Frontier in Leukemia Inhibitory Factor Receptor Modulation

Claudia Finamore,[1] Carmen Festa,[1] Mattia Cammarota,[1] Lucio Spinelli,[1] Elva Morretta,[1] Chiara Cassiano,[1] Maria Chiara Monti,[1] Silvia Marchianò,[2] Carmen Massa,[2] Federica Moraca,[1] Antonio Lupia,[3] Bruno Catalanotti,[1] Stefano Fiorucci,[2] Angela Zampella[1] and Simona De Marino[1,*]

[1]Department of Pharmacy, University of Naples "Federico II", Via D. Montesano, 49, Naples, 80131, Italy
[2]Department of Medicine and Surgery, University of Perugia, Piazza L. Severi, 1, Perugia, 06132, Italy
[3]Department of Life and Enviromental Science, University of Cagliari, - S.P. 8 Monserrato - Sestu Km 0,700 - Monserrato (CA), 09042, Italy

Table S1. Tirucallanes and α/β-amyrins putatively identified by UHPLC-MSMS analysis
Table S2. ^{1}H and ^{13}C NMR data (400 and 100 MHz, $CDCl_3$) of compounds **6** and **20**
Table S3. ^{1}H and ^{13}C NMR data (400 and 100 MHz, CD_3OD) of compounds **21-24**
Table S4. Alpha Screen and transactivation assays on LIFR of compounds **21-24**
Table S5. Docking QPLD and IFD scores with MM/GBSA (ΔG) values (kcal/mol)
Figure S1. Transactivation assay of BhE on FXR and GPBAR1
Figure S2. Chromatographic LC-MS profile of BhE with characterized compounds
Figure S3. Tirucallic acid derivatives diagnostic fragmentations
Figure S4. Boswellic acid derivatives characteristic fragmentations
Figure S5. Top-ranked docking QPLD docking pose against hLIFR of (A) Compound **1** and (B) Compound **2**
Figure S6. IFD poseA against hLIFR of (A) compound **1,** (B) compound **2**, (C) superimposition
Figure S7. IFD poseB against hLIFR of (A) compound **1** and (B) compound **2**, (C) superimposition
Figure S8. Root Mean Square Deviation (RMSD) and RMSF of hLIFR and ligands (L-RMSD) of poseA and poseB.
Figure S9. Frequencies of intermolecular hydrogen bonds over 500 ns of MDs in poseA.
Figure S10. Frequencies of intermolecular hydrogen bonds over 500 ns of MDs in poseB.
Figure S11. Ligands Solvent-accessible surface area (L-SASA) over 500 ns of MDs.

Figure S12. Conformational sampling of hLIFR complexed with (A) compound **1** posaA and (B) compound **2** poseA every 100 frames colored by timetep.
Figure S13. Conformational sampling of hLIFR complexed with (A) compound **1** posaBand (B) compound **2** poseB every 100 frames colored by timetep.
Figure S14. Hepatic antifibrotic cellular model and PCR analysis of expression of genes correlated to the activation of stellate cells
Figure S15. ^{1}H NMR ($CDCl_3$, 400 MHz) spectrum of new Compound **6**
Figure S16. HSQC ($CDCl_3$, 400 MHz) spectrum of new Compound **6**
Figure S17. ROESY ($CDCl_3$, 400 MHz) spectrum of new Compound **6**
Figure S18. ^{1}H NMR ($CDCl_3$, 400 MHz) spectrum of new Compound **20**
Figure S19. HSQC ($CDCl_3$, 400 MHz) spectrum of new Compound **20**
Figure S20. HMBC ($CDCl_3$, 400 MHz) spectrum of new Compound **20**
Figure S21. ROESY ($CDCl_3$, 400 MHz) spectrum of new Compound **20**
Figure S22. ^{1}H NMR (CD_3OD, 400 MHz) spectrum of Compound **21**
Figure S23. ^{13}C NMR (CD_3OD, 100 MHz) spectrum of Compound **21**
Figure S24. ^{1}H NMR (CD_3OD, 400 MHz) spectrum of Compound **22**
Figure S25. HSQC (CD_3OD, 400 MHz) spectrum of Compound **22**
Figure S26. HMBC (CD_3OD, 400 MHz) spectrum of Compound **22**
Figure S27. COSY (CD_3OD, 400 MHz) spectrum of Compound **22**
Figure S28. ^{1}H NMR (CD_3OD, 400 MHz) spectrum of Compound **23**
Figure S29. HSQC (CD_3OD, 400 MHz) spectrum of Compound **23**
Figure S30. HMBC (CD_3OD, 400 MHz) spectrum of Compound **23**
Figure S31. COSY (CD_3OD, 400 MHz) spectrum of Compound **23**
Figure S32. ^{1}H NMR (CD_3OD, 400 MHz) spectrum of Compound **24**
Figure S33. HSQC (CD_3OD, 400 MHz) spectrum of Compound **24**
Figure S34. HMBC (CD_3OD, 400 MHz) spectrum of Compound **24**
Figure S35. COSY (CD_3OD, 400 MHz) spectrum of Compound **24**

Table S1: Tirucallanes (TA) and α/β-amyrins putatively identified by UHPLC-MSMS analysis

ID	R_T	$[M-H]^-$	Molecular formula	Compound	Δppm	Fragments (Relative intensity)
NP 25	12.83	487.3430	$C_{30}H_{47}O_5$		2.460	355.3005 (5.8) 371.2570 (6.0) 373.2731 (5.7) 383.2941 (18.2) 397.3093 (25.0) 425.3404(6.8) 427.3205 (45.8) 441.3362 (17.7) 469.3312 (100)
NP 26	13.27	487.3428	$C_{30}H_{47}O_5$		2.050	367.2997 (7.3) 371.2587 (6.7) 383.2946 (11.7) 397.3116 (12.7) 401.2690 (7.2) 423.3264 (13.7) 427.3210 (33.8) 441.3372 (13.1) 442.3450 (20.5) 453.3362 (6.6) 455.3165 (29.7) 469.3318 (100) 470.3392 (12.5)
NP 27	13.93	471.3476	$C_{30}H_{47}O_4$		1.514	407.3335 (11.7) 409.3470 (72.3) 423.3239 (5.8) 425.3422 (95.3) 425.8844 (12.3) 427.3575 (39.3) 453.3372 (100)
NP 28	14.02	483.3114	$C_{30}H_{43}O_5$		1.860	316.2034 (6.2) 370.2503 (100) 439.3209 (35.5)
NP 29	14.40	469.332	$C_{30}H_{45}O_4$	Di-Hydroxy. dehydro TA derivative	1.627	255.1746 (18.7) 353.2484 (19.2) 355.2637 (100) 369.3157 (36.7) 387.2534 (34.8) 407.3309 (10.8) 425.3423 (26.8) 450.8440 (5.7) 451.32093 (5.3) 469.3335 (11.7)
NP 30	14.52	483.3114	$C_{30}H_{43}O_5$		1.860	370.2504 (100) 387.2540 (7.9) 411.2908 (7.6) 415.9234 (9.7) 421.3105 (22.2) 437.8623 (7.9) 439.3211 (47.2) 453.3000 (9.7)
NP 31	14.59	485.3267	$C_{30}H_{45}O_5$		1.131	287.2376 (5.7) 323.2373 (12.8) 329.247 (12.8) 367.2993 (22.2) 399.2525 (27.2) 413.3044 (6.2) 421.3116 (16.6) 423.3261 (19.1) 425.3033 (8.1) 427.2838 (11.5) 435.2898 (6.5) 439.3184 (5.3) 440.3284 (46.0) 450.3126 (8.3) 451.3217 (23.6) 453.3001 (70.6) 455.315 (20.6) 467.3159 (100) 468.3238 (39.2)
NP 32	15.09	485.3269	$C_{30}H_{45}O_5$		1.543	423.3257 (8.27) 453.3000 (100) 467.3159 (21.09)
NP 33	15.30	481.2957	$C_{30}H_{41}O_5$		1.764	314.1880 (5.7) 368.2347 (100) 437.3056 (35.2)
NP 34	15.34	467.3165	$C_{30}H_{43}O_4$	Di-Hydroxy, Di-dehydro TA derivative	1.955	255.1753 (22.4) 325.2528 (5.3) 353.2482 (100) 385.2390 (12.8) 407.2938 (5.6) 421.2384 (7.6) 423.3267 (67.4) 449.3056 (12.2)
NP 35	15.49	471.3476	$C_{30}H_{47}O_4$		1.514	373.2751 (20.8) 383.2960 (28.8) 385.3116 (100) 411.3266 (7.5) 425.3433 (29.2) 427.3587 (67.6) 453.3380 (48.0)
NP 36	15.62	485.3270	$C_{30}H_{45}O_5$		1.749	405.3151 (7.7) 423.3259 (100) 427.2842 (21.5) 437.3048 (69.8)

						438.3116 (5.2) 439.3197 (5.3) 441.3010 (14.2) 453.3004 (5.2) 457.3306 (5.6) 467.3157 (18.8) 468.3217 (8.0)
NP 37	15.82	503.3738	$C_{31}H_{51}O_5$		1.388	373.2761 (8.2) 385.3111 (100) 425.3410 (13.1) 427.3577 (65.0) 471.3488 (17.1) 485.3621 (21.4)
NP 38	16.18	501.3583	$C_{27}H_{51}O_8$		0.904	383.2945 (34.5) 423.2903 (41.38) 425.3416 (41.19) 439.3214 (100) 457.3317 (28.39) 469.3272 (5.44) 483.3116 (11.44)
NP 39	16.13	487.3424	$C_{30}H_{47}O_5$		1.229	425.3423 (9.5) 457.3326 (9.8) 469.3325 (100)
NP 40	16.23	485.3271	$C_{30}H_{45}O_5$		1.955	423.3254 (11.5) 441.3366 (100) 467.3155 (5.9)
NP 41	16.24	469.3319	$C_{30}H_{45}O_4$		1.414	301.2164 (5.2) 373.2740 (100)
NP 42	16.31	501.3220	$C_{30}H_{45}O_8$		1.864	423.2903 (45.6) 439.3218 (100) 457.3333 (27.9) 483.3120 (6.6)
NP 43	16.51	469.3319	$C_{30}H_{45}O_4$		1.414	339.2321 (5.3) 355.2614 (6.8) 373.2746 (28.4) 377.2480 (6.8) 383.2953 (10.9) 391.2993 (5.2) 393.2799 (12.1) 407.3317 (17.5) 409.3108 (100) 423.3283 (6.2) 425.3421 (82.1) 451.3210 (37.6)
NP 44	16.61	487.3424	$C_{30}H_{47}O_5$	Tri-hydroxy TA derivative	1.229	365.2834 (5.3) 373.2741 (7.6) 405.3155 (11.6) 407.3309 (23.6) 409.3469 (16.0) 411.2895 (7.8) 425.3415 (100) 427.3572 (18.8) 429.3003 (14.3) 469.3313 (10.3)
NP 45	16.75	469.3322	$C_{30}H_{45}O_4$		2.053	371.2580 (18.0) 383.2946 (100.0) 407.3312 (7.8) 423.3262 (17.1) 425.3415 (64.9) 451.3212 (27.1)
NP 46	16.89	471.3476	$C_{30}H_{47}O_4$		1.514	373.2747 (19.8) 385.3113 (100) 403.3218 (12.6) 411.3271 (17.1) 425.3426 (19.2) 427.3584 (77.7) 453.3380 (28.9)
NP 47	17.06	471.3473	$C_{30}H_{47}O_4$		0.877	373.2751 (38.0) 385.3114 (100) 385.3660 (5.5) 403.3209 (14.7) 407.3308 (6.7) 409.3071 (7.5) 409.3536 (6.7) 411.3270 (11.7) 425.3435 (23.2) 425.8823 (8.1) 427.3586 (80.8) 453.3381 (39.3)
NP 48	17.33	467.3163	$C_{30}H_{43}O_4$		1.527	371.2581 (100)
NP 49	17.40	469.3299	$C_{30}H_{45}O_4$		-2.847	373.2733 (100)
NP 50	17.40	485.3272	$C_{30}H_{45}O_5$		2.162	371.2576 (6.4) 423.3260 (14.9) 441.3359 (5.1) 455.3152 (8.7) 467.3157 (100)
NP 51	17.59	469.3319	$C_{30}H_{45}O_5$		1.414	373.2733 (100)
NP 52	17.66	487.343	$C_{30}H_{47}O_5$		2.460	373.2738 (15.3) 407.3293 (28.3) 409.3438 (20.8) 411.2891 (7.5) 425.3403 (100) 427.3575 (12.7) 429.2992 (15.4) 443.3515 (5.1) 457.3313 (7.6) 469.3309 (97.3)

NP 53	17.77	485.3273	$C_{30}H_{45}O_5$		2.368	151.1490 (11.9) 173.2738 (5.6) 192.3722 (6.6) 365.2823 (10.9) 371.2596 (12.7) 405.3168 (38.6) 407.3308 (34.4) 409.2716 (8.2) 423.3267 (100) 427.2269 (7.6) 427.2887 (19.7) 441.3397 (8.9)453.2989 (10.5) 467.3181 (46.3)
NP 54	17.97	487.3430	$C_{30}H_{47}O_5$		2.460	373.2737 (9.5) 385.3103 (5.7) 407.3311 (33.4) 409.3467 (16.8) 411.2900 (8.6) 425.3416 (100) 427.3574 (19.8) 429.3007 (15.0) 469.3317 (14.9)
NP 55	18.29	471.3467	$C_{30}H_{47}O_4$		-0.395	425.3422 (62.2) 427.3584 (100) 453.3358 (91.3)
NP 56	18.55	471.3473	$C_{30}H_{47}O_5$		0.877	423.3268 (9.6) 424.9326 (12.8) 425.3394 (91.2) 425.8814 (16.0) 427.3558 (19.0) 453.3328 (100)
NP 57	18.40	469.3324	$C_{30}H_{45}O_4$		1.414	371.2583 (35.2) 373.2740 (100) 407.3309 (5.6) 425.3418 (7.9)
NP 58	18.41	467.3162	$C_{30}H_{43}O_4$		1.420	287.2011 (5.9) 299.2012 (18.2) 353.2478 (61.5) 371.2581 (100) 395.2950 (7.2) 407.2949 (11.7) 423.3262 (48.1)
NP 59	18.68	487.3424	$C_{30}H_{47}O_5$		1.229	236.1407 (6.2) 237.1492 (100) 238.1571 (6.3) 407.3318 (10.8) 409.3108 (6.6) 423.3275 (14.1) 425.3412 (31.5) 426.3508 (7.4) 455.3161 (10.3) 469.3327 (71.2) 470.341 (11.6)
NP 60	18.72	485.3274	$C_{30}H_{45}O_5$		2.574	207.1383 (8.02) 423.3267 (100) 439.3212 (9.57) 441.3374 (42.7) 467.3169 (32.44)
NP 61	18.72	469.3323	$C_{30}H_{45}O_4$		2.266	373.2740 (100) 407.3311 (8.24)
NP 62	18.99	487.3426	$C_{30}H_{47}O_5$	Tri-hydroxy TA	1.639	151.1321 (7.4) 169.0871 (5.8) 237.1492 (80.8) 238.1569 (34.2) 405.3157 (6.7) 407.3325 (19.5) 423.3275 (22.5) 425.3423 (40.6) 455.3175 (18.4) 469.3330 (100) 470.3403 (12.0)
NP 63	19.31	469.3320	$C_{30}H_{45}O_4$		1.627	151.1319 (5.1) 289.2155 (6.3) 373.2762 (6.7) 389.3213 (6.4) 391.2983 (10.6) 405.3152 (6.9) 407.3310 (37.2) 421.3099 (13.2) 423.3260 (100) 425.3412 (50.0) 451.3211 (91.7)
NP 64	19.41	485.3269	$C_{30}H_{45}O_5$		1.543	371.2589 (5.8) 389.2836 (6.3) 399.2531 (24.9) 405.3156 (12.1) 407.2949 (16.4) 421.3109 (23.5) 423.3265 (37.8) 439.3203 (6.1) 453.3008 (14.3) 467.3164 (100) 468.3244 (64.0)
NP 65	19.48	487.3416	$C_{30}H_{47}O_5$		-0.412	181.4618 (8.1) 216.6073 (5.4) 407.3304 (7.5) 409.8928 (5.4) 425.3424 (100) 441.3349 (9.9) 442.3463 (27.5) 469.3305 (31.7) 470.3333 (7.5)

NP 66	19.83	469.3320	$C_{30}H_{45}O_4$		1.627	377.3211 (7.64) 389.3216 (6.35) 391.3003 (37.12) 407.3316 (100) 423.3265 (11.55) 425.3402 (5.66) 451.3219 (20.37)
**7**	20.01	469.3319	$C_{30}H_{45}O_4$	**Compound 7** 11-Keto boswellic acid	1.414	355.2643 (35.3) 373.2749 (25.1) 391.3005 (25.9) 407.3319 (100) 409.3112 (13.7) 423.3262 (9.5) 425.3430 (41.2) 451.3210 (14.9)
NP 67	20.09	485.3269	$C_{30}H_{45}O_5$		1.543	353.2476 (5.36) 371.2579 (6.58) 389.2837 (9.7) 399.2533 (6.85) 405.3156 (10.9) 407.2951 (7.21) 423.2803 (12.12) 423.3260 42.45) 439.3213 (52.21) 51.3214(32.58) 452.3289 (12.6) 467.3162 (100) 468.3230 (10.84)
NP 68	20.10	487.3420	$C_{30}H_{47}O_5$		0.408	407.3308 (19.71) 425.338 (8.6) 469.3311 (100)
NP 69	20.66	487.3426	$C_{30}H_{47}O_5$		1.639	221.1182 (12.2) 236.1416 (37.4) 237.1494 (100) 238.1571 (51.8) 265.1444 (5.8) 277.1442 (14.0) 278.1518 (7.9) 386.2449 (5.1) 407.3315 (7.5) 425.3414 (19.2) 441.3368 (16.1) 442.3450 (9.5) 455.3165 (32.9) 469.3317 (59.5) 470.3395 (57.6)
NP 70	21.04	469.3319	$C_{30}H_{45}O_4$		1.414	373.2887 (84.4) 389.3205 (100) 397.3107 (7.7) 407.3310 (18.0) 421.3099 (29.0) 423.3265 (9.9) 425.3417 (6.9) 451.3210 (20.5)
NP 71	21.47	471.3476	$C_{30}H_{47}O_4$		1.514	257.97 (8.12) 279.01 (7.28) 377.2850 (9.93) 469.3319 (7.26)
NP 72	21.47	487.3424	$C_{30}H_{47}O_5$		1.229	397.3097 (6.81) 425.3414 (18.82) 441.3369 (100) 469.3317 (21.26)
NP 73	21.48	469.3319	$C_{30}H_{45}O_4$		1.414	407.3306 (8.5) 421.3104 (100) 425.3405 (6.7)
NP 74	21.60	471.3476	$C_{30}H_{47}O_4$	Di-hydroxy TA derivative	1.514	373.2895 (100) 375.3051 (44.4) 389.2836 (17.7) 405.3162 (8.4) 409.3459 (6.7) 423.3251 (9.7) 425.3418 (21.2) 427.3579 (5.5) 453.3368 (53.1)
NP 75	21.78	485.3271	$C_{30}H_{45}O_5$		1.955	151.1315 (5.9) 397.3106 (44.4) 405.3148 (21.4) 407.3312 (9.6) 423.3263 (23.9) 437.3430 (7.2) 439.3566 (86.4) 441.3333 (7.3) 467.3516 (100)
NP 76	21.90	471.3476	$C_{30}H_{47}O_4$		1.514	423.3260 (100)
NP 77	21.98	469.332	$C_{30}H_{45}O_4$	Di-Hydroxy. de-hydro TA derivative	1.627	355.2634(46.89) 381.2790 (30.29) 387.2525 (7.69) 395.2950 (36.45) 396.3026(16.73) 397.3106 (25.04) 407.3311 (70.55) 423.3256 (5.75) 425.3416 (100) 451.3214 (38.61)
**16**	22.18	455.3527	$C_{30}H_{47}O_3$	Compound **16** 3-hydroxy tirucallic acid	1.599	151.1494 (10.6) 339.2685 (100) 359.2916 (6.1) 373.2736 (64.0) 425.3075 (6.0) 437.3418 (50.2) 455.3529 (11.6)
NP 78	22.25	471.3474	$C_{30}H_{47}O_4$		1.090	409.3465 (9.42) 423.3261 (100)

NP 79	22.32	469.3321	$C_{30}H_{45}O_4$	Di-Hydroxy. dehydro TA derivative	1.840	151.1532 (10.5) 218.7768 (9.6) 355.2634 (100) 381.2785 (32.4) 387.2527 (13.5) 395.2935 (30.6) 396.3014 (12.5) 397.3097 (27.6) 423.3310 (12.2) 423.5360 (11.8) 425.3415 (20.3) 451.3204 (77.3)
NP 80	22.50	469.3319	$C_{30}H_{45}O_4$		1.414	351.3051 (100) 407.3312 (54.5) 451.3216 (8.2)
NP 81	22.63	451.3215	$C_{30}H_{43}O_3$	Hydroxy. Di-dehydro TA derivative	1.835	151.1469 (6.2) 239.1437 (11.3) 241.1594 (56.1) 269.1906 (100) 283.1700 (5.4) 337.2531 (8.6) 369.2434 (5.2) 391.3004 (14.9) 421.2745 (39.5)
**14**	22.85	455.3524	$C_{30}H_{47}O3$	Compound **14** Hydroxy TA derivative	0.940	121.2632 (9.8) 124.3439 (10.1) 140.1262 (9.8) 140.7101 (10.6) 141.6423 (12.2) 146.9923 (9.6) 151.1129 (16.5) 151.1320 (86.1) 151.1675 (16.9) 162.8083 (11.9) 186.4526 (12.8) 243.5144 (13.7) 359.2913 (13.3) 373.2743 (100) 425.3091 (15.1) 437.3409 (77.6) 455.3520 (17.7)
NP 82	22.92	469.332	$C_{30}H_{45}O_4$	Di-Hydroxy. dehydro TA derivative	1.627	351.3052 (53.7) 373.2893 (35.8) 387.3054 (5.8) 389.3208 (81.1) 407.3314 (100) 421.3107 (11.1) 425.3417 (22.6) 451.3215 (9.7)
NP 83	23.11	451.3217	$C_{30}H_{43}O_3$	Hydroxy. Di-dehydro TA	2.278	125.4215 (17.6) 128.0259 (15.7) 151.1292 (49.3) 151.1552 (29.3) 181.4650 (44.8) 337.2536 (100) 347.5337 (29.3) 354.3246 (21.5) 369.2436 (76.5) 391.3007 (68.5) 407.3322 (32.9) 433.3086 (79.6)
**17**	23.26	453.3371	$C_{30}H_{45}O_3$	Compound **17** 3-oxo TA	1.717	134.9516 (11.0) 151.0954 (9.1) 151.1166 (13.2) 151.1477 (66.4) 151.1629 (14.7) 207.1268 (12.5) 339.2683 (100) 371.2581 (96.1) 391.3335 (28.9) 397.3106 (53.0) 407.3178 (14.4) 435.3258 (77.7) 453.3378 (15.8)
**15**	23.34	455.3516	$C_{30}H_{47}O_3$	Compound **15** Hydroxy TA Isomer	- 0.817	129.6152 (29.6) 143.7053 (38.1) 147.5337 (36.3) 151.1510 (100) 341.2812 (56.7) 373.2701 (86.0) 437.3381 (68.6)
NP 84	23.56	471.3476	$C_{30}H_{47}O_4$	Di-Hydroxy TA derivative	1.514	355.2629 (18.9) 357.2790 (100) 383.2944 (13.0) 389.2679 (20.8) 397.3130 (7.4) 399.3260 (14.9) 409.3474 (18.6) 425.3435 (11.8) 427.3578 (30.3) 453.3373(58.2)
NP 85	23.70	455.3523	$C_{30}H_{47}O_3$		0.721	437.3428 (100) 445.6020 (7.8)
**8**	23.96	453.3374	$C_{30}H_{45}O_3$	Compound **8** 9.11-Dehydro boswellic acid	2.378	375.3047 (11.1) 391.3361 (25.7) 397.3103 (100) 407.3310 (8.0)
**1**	24.43	455.3528	$C_{30}H_{47}O_3$	Compound **1** α-boswellic acid	1.819	375.3051 (10.6) 377.3195 (5.4) 407.332 (9.0) 409.3465 (95.2) 409.9066 (5.8) 437.3417 (100)

NP 86	24.57	485.3634	$C_{31}H_{49}O_4$		1.779	151.1326 (8.14) 256.9232 (5.52) 375.3041 (6.51) 405.3158 (9.75) 407.3315 (11.66) 423.3257 (5.81) 437.3398 (5.85) 438.846 (6.71) 438.9085 (5.19) 439.3577 (80.73) 439.8431 (16.21) 467.3529 (100)
NP 87	24.68	441.337	$C_{29}H_{45}O_3$		1.537	304.9130 (5.1) 363.3043 (5.1) 365.2838 (17.5) 367.2997 (8.9) 381.3149 (95.4) 397.3463 (100) 423.3259 (34.6)
NP 88	24.75	471.3474	$C_{30}H_{47}O_4$		1.090	393.3150 (6.1) 409.3464 (100) 427.3573 (25.0)
**2**	24.79	455.3528	$C_{30}H_{47}O_3$	Compound **2** β-boswellic acid	1.819	361.2878 (5.2) 377.3206 (27.7) 391.3005 (13.3) 407.3329 (10.1) 409.3470 (86.5) 427.3203 (5.4) 437.3423 (100)
NP 89	25.12	479.3526	$C_{32}H_{47}O_3$		1.310	390.2889 (8.9) 397.2738 (100) 409.2765 (18.0) 410.9106 (21.7) 411.9075 (11.1) 432.9563 (9.0) 433.3136 (9.2) 433.8533 (16.2) 435.3643 (25.0) 449.312 (8.2) 461.3424 (72.8)
NP 90	25.47	441.3369	$C_{29}H_{45}O_3$		1.129	394.8327 (13.6) 394.8978 (6.2) 395.3313(100) 397.3467 (35.8) 412.8421 (7.7) 423.3264 (91.5)
NP 91	25.58	497.3629	$C_{32}H_{49}O_4$	Acetyl hydroxy TA derivative	1.535	415.2845 (17.8) 430.0079 (13.8) 437.3415 (100) 451.9897 (12.2) 479.3520 (10.4)
NP 92	25.73	453.337	$C_{30}H_{45}O_3$		1.496	391.3362 (100) 397.3102 (65.9) 407.3317 (56.1) 435.3257 (81.8)
NP 93	25.83	441.3368	$C_{29}H_{45}O_3$		1.084	395.3321 (100) 397.3483 (8.84) 412.8427 (6.91) 423.3271 (79.76)
NP 94	26.18	453.3371	$C_{30}H_{45}O_3$		1.717	391.3364 (18.15) 397.3105 (100) 407.3304 (7.73) 435.3275 (7.71)
NP 95	26.39	453.3369	$C_{30}H_{45}O_3$		1.276	391.3360 (100) 397.3100 (96.6) 405.3166 (8.5) 407.3307 (22.8) 435.3265 (33.7)
NP 96	26.61	453.3371	$C_{30}H_{45}O_3$		1.717	375.3047 (17.0) 391.3356 (39.2) 397.3106 (100) 407.3306 (12.3) 435.3263 (13.5)
NP 97	26.82	455.3521	$C_{30}H_{47}O_3$		0.282	409.3469 (78.1) 409.9074 (28.1) 437.3423 (100) 437.4109 (5.0)
NP 98	27.19	455.3525	$C_{30}H_{47}O_3$		1.160	375.3051 (10.3) 377.3208 (8.3) 391.3001 (5.5) 407.3327 (9.0) 409.3472 (90.4) 409.9081 (15.3) 437.3423 (100)
NP 99	27.44	455.3528	$C_{30}H_{47}O_3$		1.819	361.2885 (5.1) 377.3204 (35.3) 391.2989 (15.8) 393.2773 (5.3) 407.3306 (8.2) 409.3464 (83.5) 409.9080 (8.5) 427.3204 (7.6) 437.3415 (100)
**3**	28.56	497.3633	$C_{32}H_{49}O_4$	Compound **3** 3-Acetyl α-boswellic acid	1.535	447.8221 (23.74) 450.8129 (22.82) 450.8741 (5.38) 451.8687 (24.98) 453.8536 (12.50) 461.818 (7.10) 478.7694 (8.28) 479.8264 (100)

**4**	29.93	497.3636	$C_{32}H_{49}O_4$	Compound **4** 3-Acetyl β - boswellic acid	2.138	447.8209 (23.52) 450.7491 (5.01) 450.815 (20.41) 451.8683 (17.82) 453.8526 (7.90) 461.8191 (5.68) 478.7667 (7.44) 479.8252 (100)
NP 100	30.92	469.332	$C_{30}H_{45}O_4$		1.499	391.2998 (29.1) 407.3309 (100) 423.3261 (7.9) 451.3213 (15.4)
NP 101	34.44	469.332	$C_{30}H_{45}O_4$		1.627	318.9483 (7.0) 391.2999 (26.5) 407.3311 (100) 422.9171 (5.9) 423.3262 (8.5) 451.3215 (15.4)
NP 102	37.72	453.337	$C_{30}H_{45}O3$		1.496	406.9131 (20.3) 435.3272 (100)
NP 103	39.57	455.3527	$C_{30}H_{47}O3$		1.599	407.3311 (7.5) 409.3472 (100) 409.9075 (10.2) 437.3423 (98.1) 437.4105 (5.1)

Table S2. ^{1}H and ^{13}C (400 and 100 MHz, $CDCl_3$) NMR data of new compounds **6** and **20**

	**20**		**6**	
	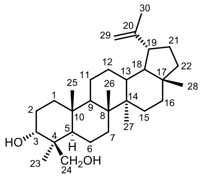		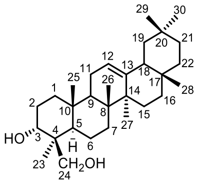	
**Position**	δ_H	δ_C	δ_H	δ_C
**1**	1.41 (m)<br>1.26 (m)	33.5	1.36 (m)<br>1.29 (m)	33.1
**2**	1.88 (m)<br>1.59 (m)	25.4	1.90 (m)<br>1.60 (m)	24.5
**3**	3.87 (br t, *J*= 3.3 Hz)	70.6	3.87 (br t, *J*=2.5 Hz)	70.2
**4**	-	42.9	-	42.1
**5**	1.33 (m)	49.6	1.38 (m)	49.0
**6**	1.48 (m)<br>1.33 (m)	18.4	1.53 (m)<br>1.38 (m)	17.8
**7**	1.46 (m)<br>1.40 (m)	33.7	1.53 (m)<br>1.36 (m)	32.6
**8**	-	40.9	-	40.4
**9**	1.41 (m)	50.3	1.70 (m)	47.0
**10**	-	37.1	-	36.6
**11**	1.45 (m)<br>1.20 (m)	20.9	1.90 (m)	22.8
**12**	1.66 (m)<br>1.08 (m)	25.0	5.18 (br t, *J*=3.4 Hz)	120.9
**13**	1.67 (m)	38.1	-	145.3
**14**	-	42.8	-	42.0
**15**	1.67 (m)<br>1.01 (m)	27.3	1.90 (m)<br>0.95 (m)	25.3
**16**	1.48 (m)<br>1.38 (m)	35.3	2.00 (m)<br>0.80 (m)	26.1
**17**	-	42.9	-	31.9
**18**	1.38 (m)	48.2	1.94 (m)	46.5
**19**	2.39 (m)	47.9	1.65 (m)<br>1.01 (m)	46.1
**20**	-	150.9	-	30.2
**21**	1.93 (m)<br>1.28 (m)	29.7	1.32 (m)<br>1.10 (m)	34.2
**22**	1.38 (m)<br>1.21 (m)	39.9	1.42 (m)<br>1.23 (m)	36.4
**23**	1.09 (s)	21.3	1.10 (s)	21.0
**24**	3.73 (d, *J*=11.0 Hz)<br>3.54 (d, *J*= 11.0 Hz)	66.6	3.74 (d, *J*=11.0 Hz)<br>3.56 (d, *J*=11.0 Hz)	65.9
**25**	0.84 (s)	16.5	0.94 (s)	15.8
**26**	1.03 (s)	15.7	0.96 (s)	16.0
**27**	0.97 (s)	14.5	1.15 (s)	25.2
**28**	0.79 (s)	17.8	0.84 (s)	27.8
**29**	4.70 (br d, *J*=2.4 Hz, H29-a)<br>4.58 (dd, *J* = 2.5, 1.4 Hz, H29-b)	109.4	0.88 (s)	33.0
**30**	1.69 (s)	19.2	0.88 (s)	22.9

Table S3. ^{1}H and ^{13}C (400 and 100 MHz, CD_3OD) NMR data of compounds **21-24**

	21		22		23		24	
	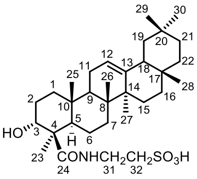		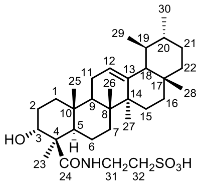		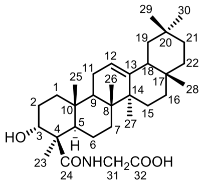		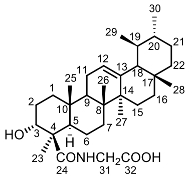	
**Position**	δ_H	δ_C	δ_H	δ_C	δ_H	δ_C	δ_H	δ_C
**1**	1.31 (m)<br>1.41 (m)	35.3	1.39 (m)<br>1.46 (m)	35.5	1.15 (m)<br>1.41 (m)	35.6	1.37 (m)<br>1.42 (m)	35.3
**2**	1.53 (m)<br>2.27 (m)	27.1	1.56 (m)<br>2.29 (m)	27.3	1.53 (m)<br>2.25 (m)	27.2	1.58 (m)<br>2.31 (m)	27.2
**3**	3.98 (t, *J* = 2.5 Hz)	71.5	4.00 (t, *J* = 2.7 Hz)	71.5	3.98 (br t, *J* = 2.3 Hz)	71.3	4.00 (t, *J*= 2.5 Hz)	71.4
**4**	-	48.1	-	48.6	-	48.4	-	48.9
**5**	1.55 (m)	50.2	1.53 (m)	50.2	1.54 (m)	50.1	1.55 (m)	50.4
**6**	1.82 (m)<br>1.88 (m)	21.2	1.78 (m)<br>1.85 (m)	21.2	1.81 (m)<br>1.87 (m)	20.9	1.78 (m)<br>1.85 (m)	21.2
**7**	1.48 (m)<br>1.68 (m)	34.3	1.48 (m)<br>1.62 (m)	34.5	1.45 (m)<br>1.61 (m)	34.1	1.48 (m)<br>1.65 (m)	34.7
**8**	-	41.1	-	41.3	-	41.1	-	41.3
**9**	1.70 (m)	48.0	1.70 (m)	48.2	1.70 (m)	47.9	1.68 (m)	48.2
**10**	-	38.7	-	38.6	-	38.4	-	38.6
**11**	1.91 (m)	24.6	1.93 (m)	24.4	1.89 (m)	24.4	1.95 (m)	24.3
**12**	5.21 (t, *J* = 3.5 Hz)	123.5	5.17 (br t, *J* = 3.6 Hz)	126.2	5.23 (br t, *J* = 3.6 Hz)	123.3	5.17 (t, *J* = 3.4 Hz)	126.2
**13**	-	146.2	-	140.8	-	145.9	-	140.8
**14**	-	43.1	-	43.4	-	42.8	-	43.3
**15**	1.02 (m)<br>1.83 (m)	27.4	1.04 (m)<br>1.57 (m)	27.6	1.03 (m)<br>1.83 (m)	26.9	1.06 (m)<br>1.90 (m)	27.6
**16**	0.84 (m)<br>2.05(m)	28.0	0.90 (m)<br>2.08 (m)	29.2	0.83 (m)<br>2.05(m)	27.8	0.90 (m)<br>2.08 (m)	29.1
**17**	-	33.6	-	34.8	-	33.2	-	34.8
**18**	1.99 (m)	49.6	1.35 (m)	60.5	1.98 (m)	48.6	1.35 (m)	60.5
**19**	1.03 (m)<br>1.77 (m)	48.0	1.38 (m)	41.0	1.03 (m)<br>1.76 (m)	47.8	1.38 (m)	41.0
**20**	-	31.9	0.88 (m)	40.8	-	31.7	0.88 (m)	40.9
**21**	1.10 (m)<br>1.38 (m)	35.8	1.32 (m)<br>1.41 (m)	32.3	1.11 (m)<br>1.38 (m)	35.2	1.31 (m)<br>1.41 (m)	32.3
**22**	1.24 (m)<br>1.47 (m)	38.3	1.32 (m)<br>1.45 (m)	42.6	1.24 (m)<br>1.47 (m)	38.0	1.32 (m)<br>1.46 (m)	42.6
**23**	1.23 (s)	25.4	1.23 (s)	25.4	1.26 (s)	25.2	1.27 (s)	25.2
**24**	-	179.5	-	179.6	-	179.2	-	179.8
**25**	0.89 (s)	13.9	0.90 (s)	14.1	0.89 (s)	13.5	0.89 (s)	14.1
**26**	1.04 (s)	17.4	1.09 (s)	17.4	1.04 (s)	17.2	1.10 (s)	17.3
**27**	1.19 (s)	26.4	1.15 (s)	23.6	1.20 (s)	26.2	1.15 (s)	23.5
**28**	0.86 (s)	28.9	0.83 (ovl)	29.0	0.85 (s)	28.8	0.83 (ovl)	29.2
**29**	0.88 (ovl)	24.1	0.83 (ovl)	17.7	0.88 (ovl)	23.8	0.83 (ovl)	17.9
**30**	0.88 (ovl)	33.8	0.94 (s)	21.5	0.88 (ovl)	33.6	0.94 (ovl)	21.6
**31**	3.59 (m)	43.1	3.61 (m)	36.4	3.81 (br s)	42.8	3.88 (d, *J* = 8.3 Hz)	41.8
**32**	2.95 (t, *J* = 6.2 Hz)	51.1	2.93 (t, *J* = 6.2 Hz)	51.1	-	182.4	-	173.3

Table S4. Alpha Screen and transactivation assays on LIFR of compounds **21-24**

	**Alpha Screen**			**Transactivation**
Compound	Inhibition (%), 10 μM	Inhibition (%), 50 μM	IC_{50}	Inhibition (%), 50 μM
**21**	45.43	67.69	7.86	68.8
**22**	51.28	83.66	2.54	58.3
**23**	52.86	88.53	3.13	70.2
**24**	48.69	86.89	4.45	74.8

Table S5. Docking QPLD and IFD scores with MM/GBSA (ΔG) values calculated respectively, by Glide and Prime modules of Schrödinger 2022. MM/GBSA cl0 is the ΔG value calculated with the MMPBSA.py script of the AMBER22 package on the MDs frames belonging to the most populated cluster (cl0) after 500 ns. All the values are expressed as kcal/mol.

	**QPLD**				**IFD**				**MDs (cl0)**
Ligand	*QPLD rank*	*G-Score*	*Emodel*	*ΔG (MMGBSA)*	*IFD rank*	*Emodel*	*IFD-Score*	*ΔG (MMGBSA)*	*ΔG (MM/GBSA)*
**1**	Pose 1	-4.019	-40.408	-51.46	poseA	-81.981	-10597.21	-114.08	-38.49
					poseB	-78,962	-10590.96	-106.38	-41.29
**2**	Pose 1	-3.719	-33.176	-55.56	poseA	-116.553	-12082.40	-132.95	-38.70
					poseB	-117.386	-12081.51	-132.39	-30.81
	Pose 2	-3.649	-36.836	-51.31	-	-	-	-	-

Figure S1. Transactivation assay of BhE on FXR and GPBAR1

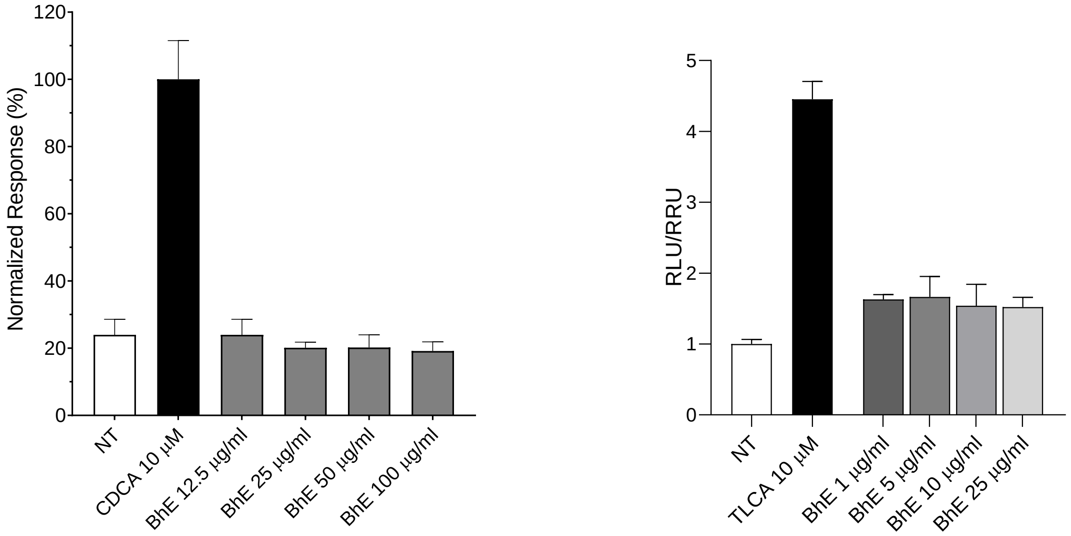

Figure S2. Chromatographic LC-MS profile of BhE with annotated chromatographic peaks of the ten fully characterized compounds

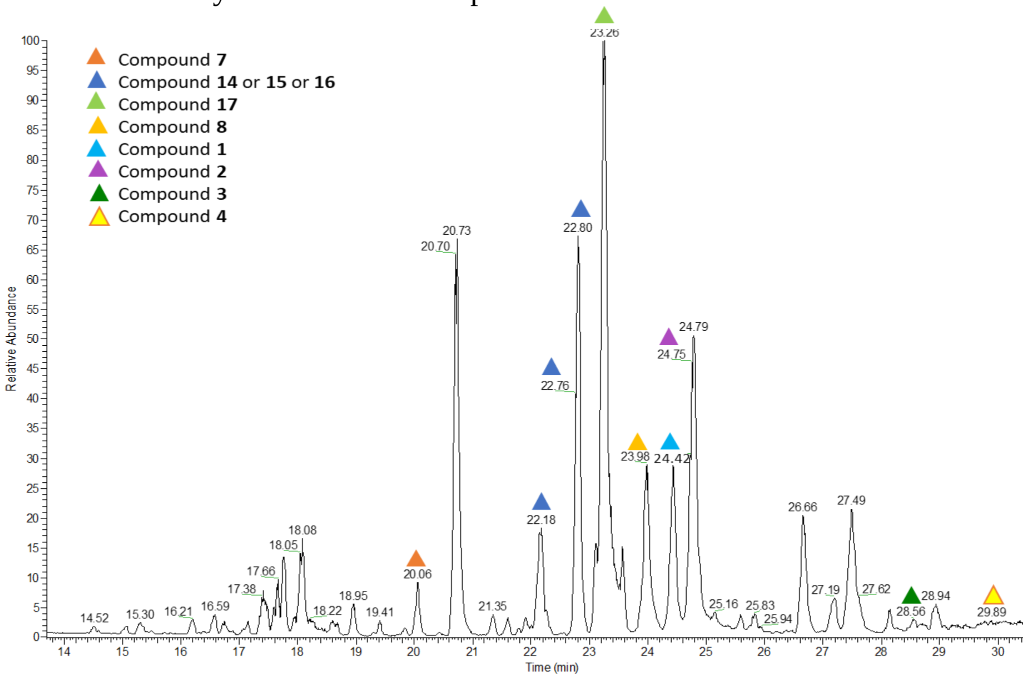

Figure S3: Tirucallic acid derivatives (m/z of 455.35) diagnostic fragmentation. The first daughter ion is due to the side chain loss thought a McLafferty rearrangement (m/z of 373.27). This specie is capable of losing one molecule of oxygen giving rise to the ion at m/z of 341.28 and/or losing acetic acid (m/z of 313.25).

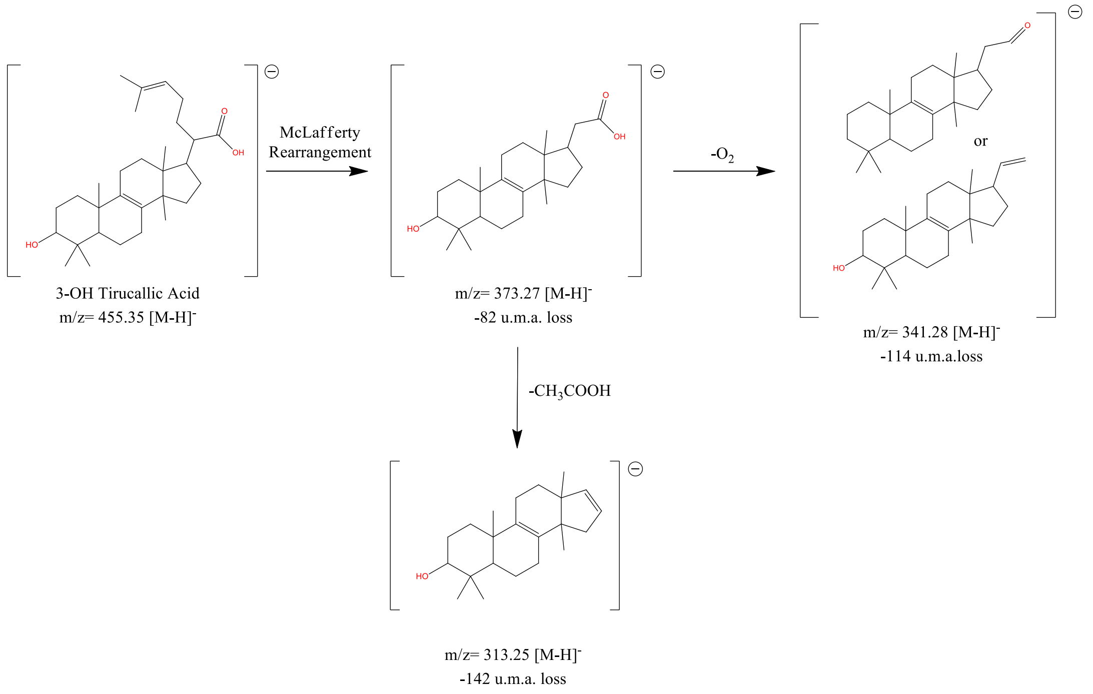

Figure S4: Boswellic acids characteristic fragmentation. The carboxylic acid in position 4 induces bonds reorganization favoring the subsequent McLafferty rearrangement followed by the opening of ring A and the loss of two hydrogens molecules.

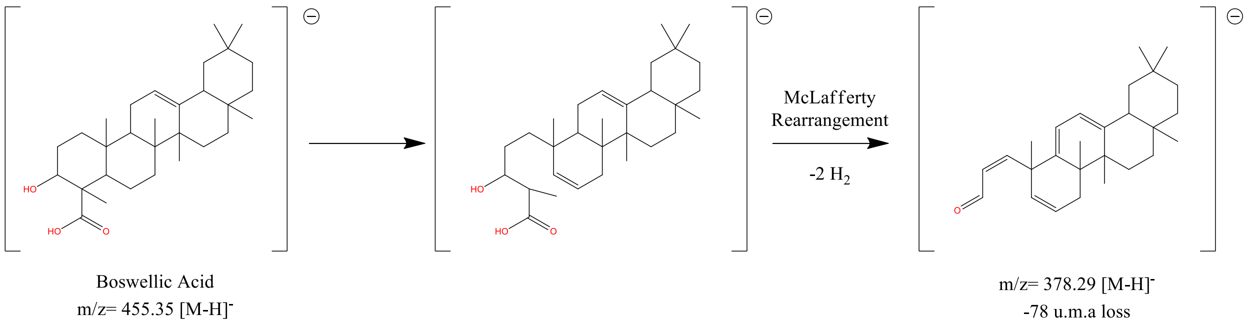

Figure S5. Top-ranked docking QPLD docking pose of (A) Compound **1** (cyan stick) and (B) Top-ranked QPLD pose of compound **2** (magenta stick); (C) Second-ranked QPLD pose of compound **2**. *h*LIFR is displayed as green cartoon, while the loops L1, L2 and L3 are colored in brown, purple and orange cartoon, respectively. Hydrogen bond interactions are depicted as dashed black lines.

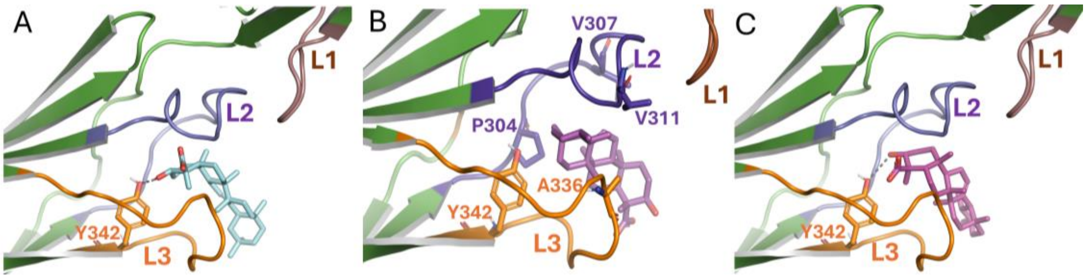

Figure S6. IFD poseA of (A) Compound **1** (cyan stick) and (B) Compound **2** (magenta stick); (C) Superimposition of the IFD poseA between **1** and **2** highlighting the different position in the plane of the triterpene moiety. *h*LIFR is displayed as green cartoon, while the loops L1, L2 and L3 are colored in brown, purple and orange cartoon, respectively. Hydrogen bond interactions are depicted as dashed black lines.

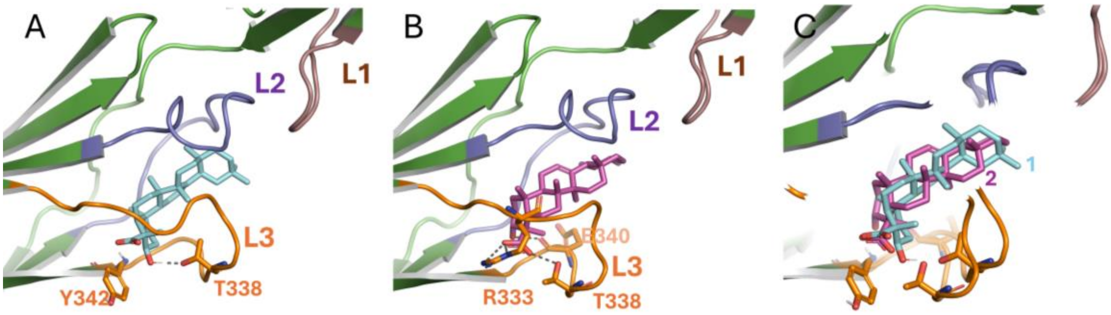

Figure S7. IFD poseB of (A) Compound **1** (cyan stick) (B) Compound **2** (magenta stick); (C) Superimposition of the IFD poseB between **1** and **2** highlighting the different position in the plane of the triterpene moiety *h*LIFR is displayed as green cartoon, while the loops L1, L2 and L3 are colored in brown, purple and orange cartoon, respectively. Hydrogen bond interactions are depicted as dashed black lines.

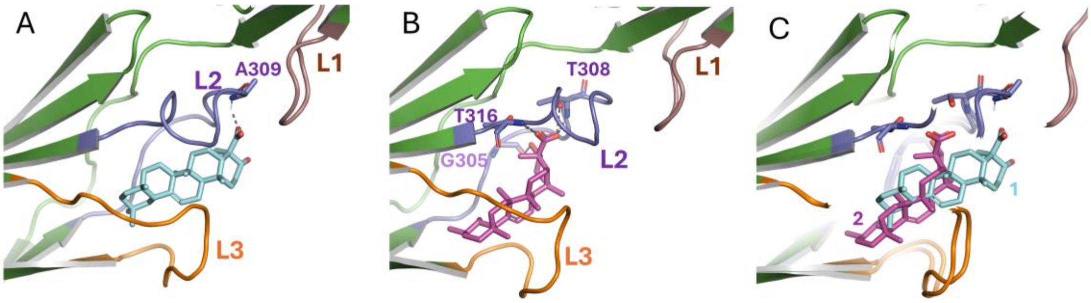

Figure S8. (A-B) Root Mean Square Deviation (RMSD) trend along 500 ns of MDs of compound **1** and **2** in poseA poseB, respectively. **(C-D)** RMSD of D3-D4 domains of *h*LIFR in its apo state (blue lines) and *h*LIFR/**1** (black lines) and *h*LIFR/**2** (red lines) complexes in poseA and poseB, respectively. **(E-F)** Root Mean Square Fluctuations (RMSF) of D3-D4 domains of *h*LIFR in its apo state (blue lines) and *h*LIFR/**1** (black lines) and *h*LIFR/**2** (red lines) complexes in poseA and poseB, respectively.

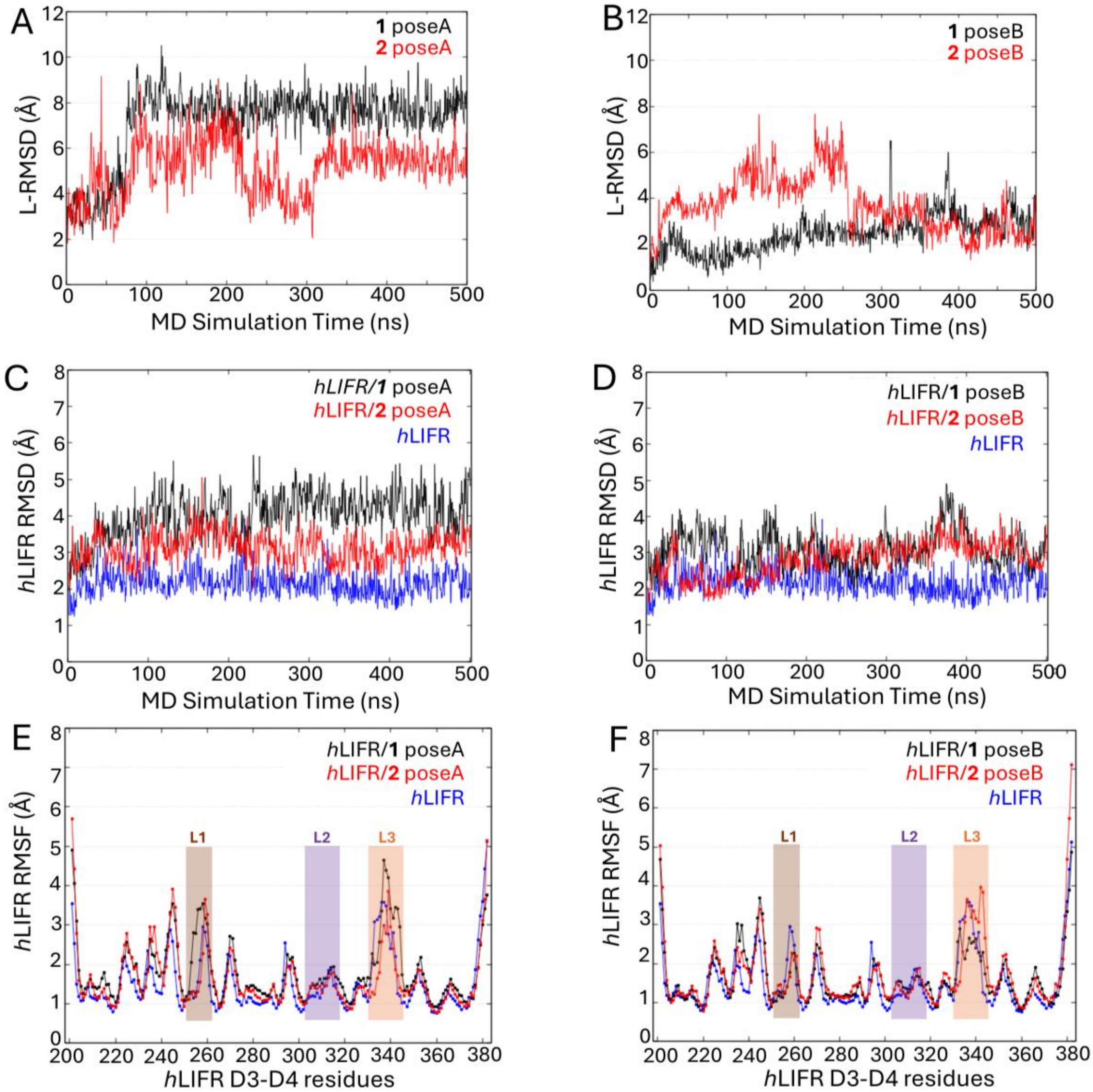

Figure S9: Frequencies of intermolecular hydrogen bonds over 500 ns of MDs between the L3-L2 residues of (A-B) *h*LIFR/**1** and (C-F) *h*LIFR/**2** complexes, respectively. Hydrogen bonds were detected only if the distance between donor and acceptor was not higher than 3.5 Å and the angle was not lower than 120°. Water-mediated hydrogen bonds are excluded.

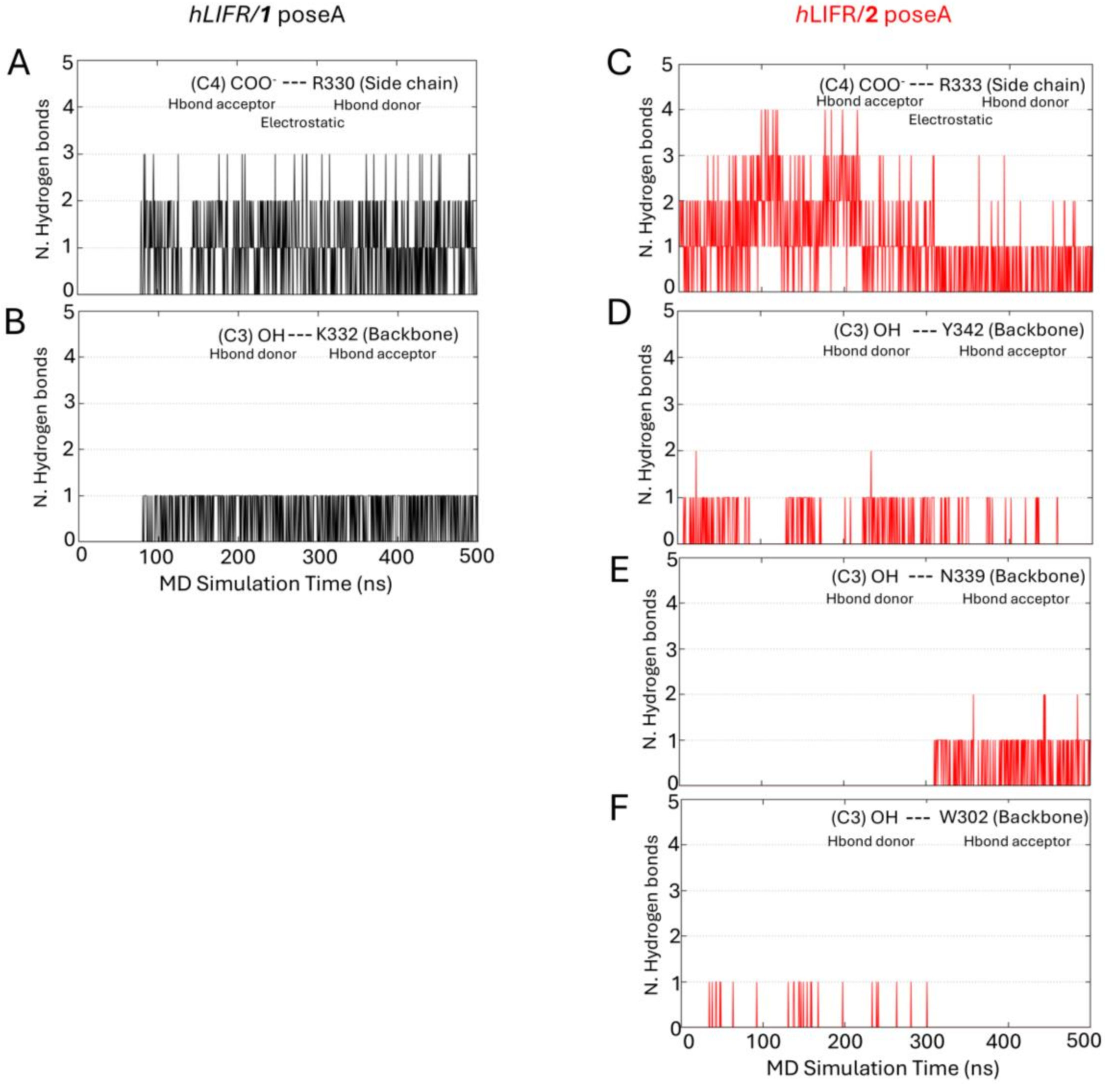

Figure S10: Frequencies of intermolecular hydrogen bonds over 500 ns of MDs between the L3-L2 residues of (A-B) *h*LIFR/**1** and (C-F) *h*LIFR/**2** complexes, respectively. Hydrogen bonds were detected only if the distance between donor and acceptor was not higher than 3.5 Å and the angle was not lower than 120°. Water-mediated hydrogen bonds are excluded.

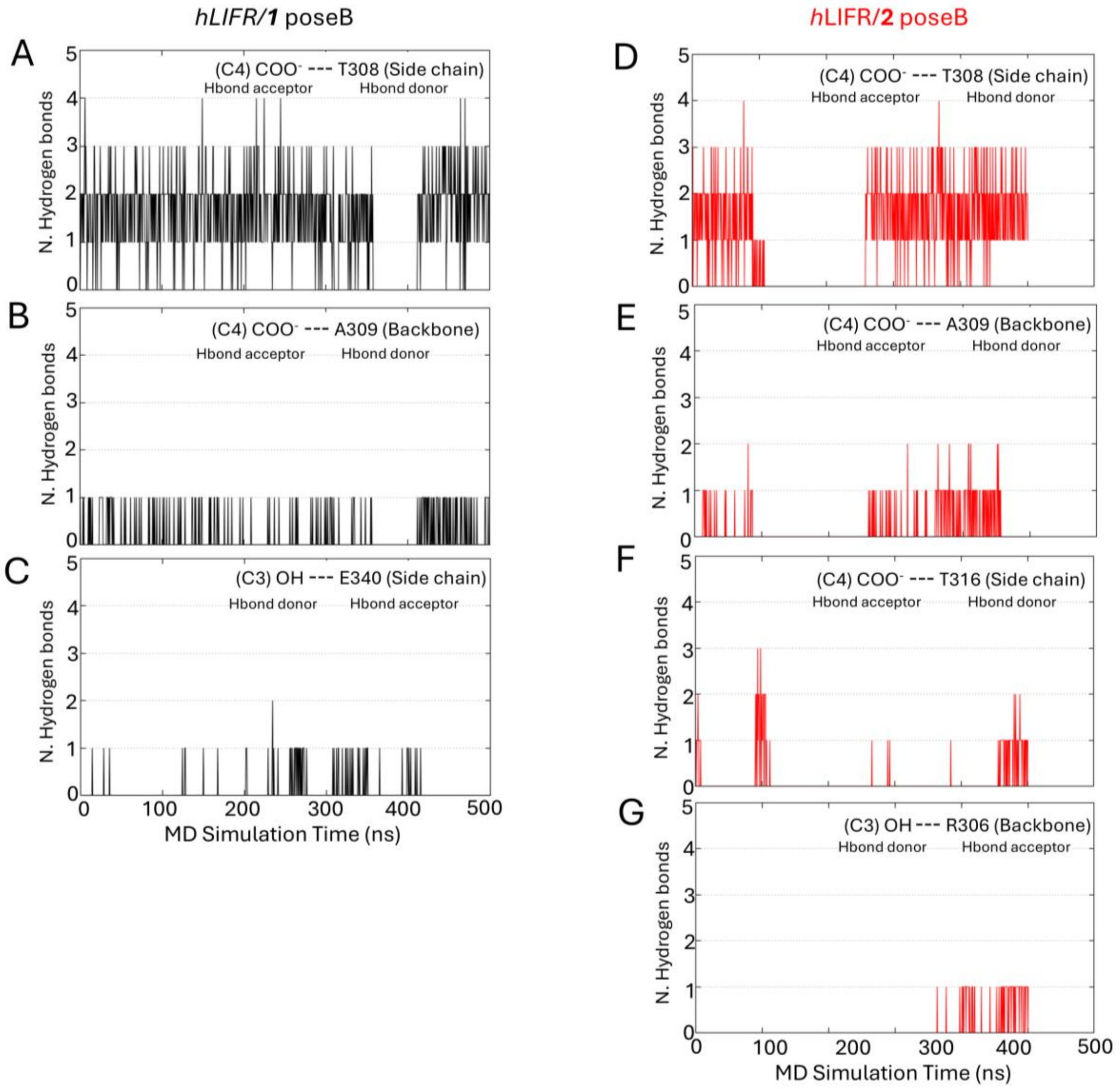

Figure S11: Ligands Solvent-accessible surface area (L-SASA) over 500 ns of MDs in (A) **1** and **2** in poseA and (B) **1** and **2** poseB

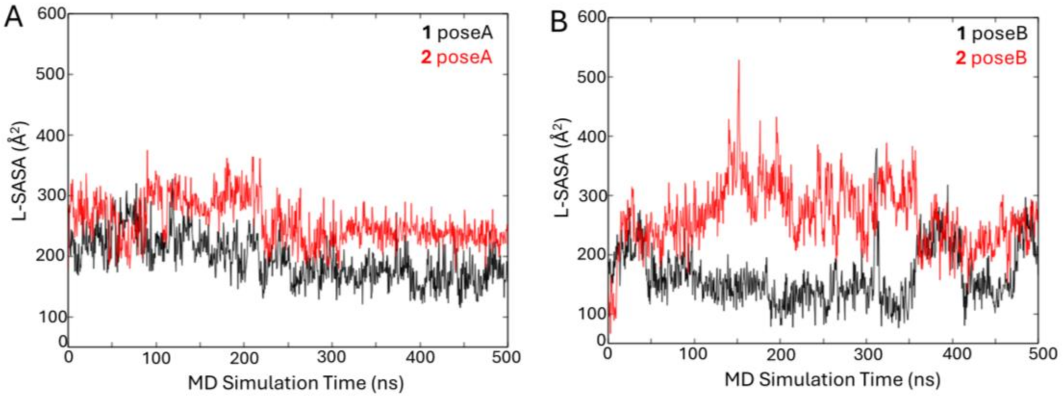

Figure S12: Image of every hundredth frame of *h*LIFR D3-D4 domains in: **(A)** hLIFR/**1** and **(B)** hLIFR/**2** in poseA, smoothed with a 2-frame window and colored by timestep so that the beginning of the trajectory (t_0) is colored in red, the middle (t_{250}) in white and the end (t_{500}) in blue gradient.

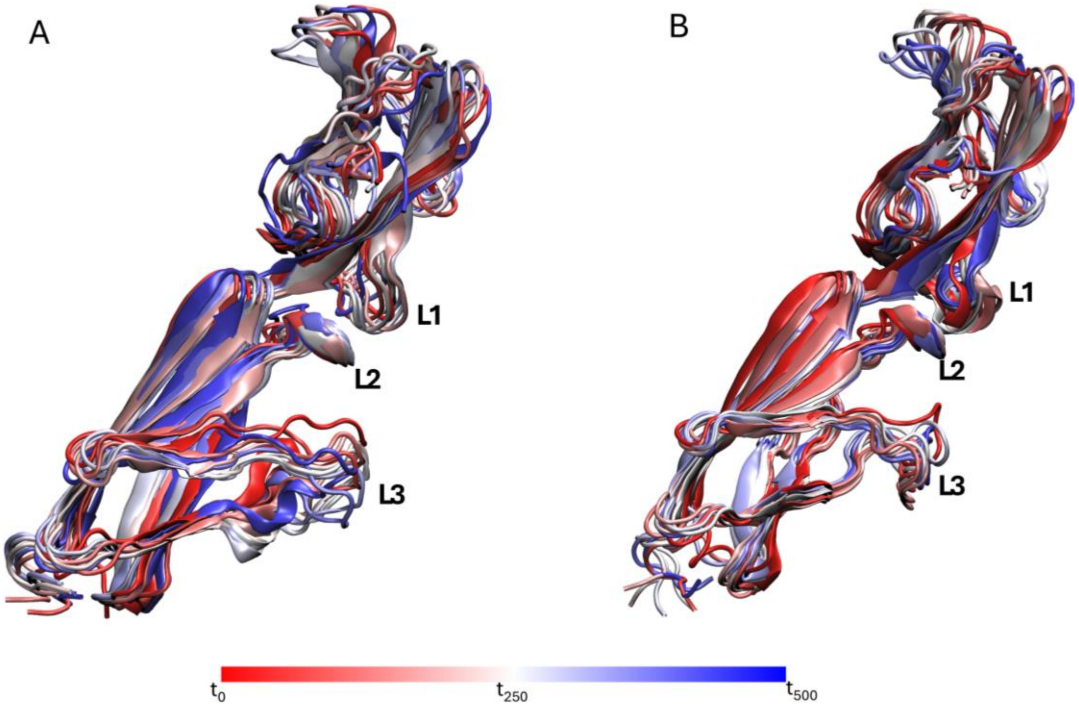

Figure S13: Image of every hundredth frame of *h*LIFR D3-D4 domains in **(A)** *h*LIFR/**1** and **(B)** *h*LIFR/**2** in poseB smoothed with a 2-frame window and colored by timestep so that the beginning of the trajectory (t_0) is colored in red, the middle (t_{250}) in white and the end (t_{500}) in blue gradient.

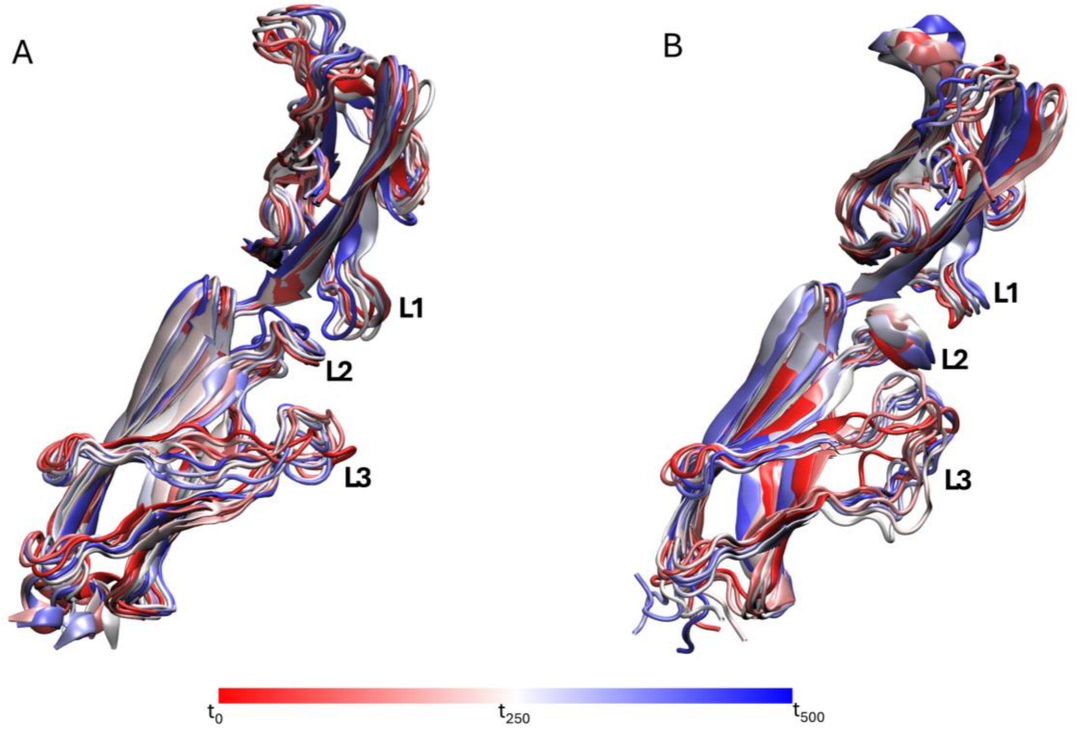

Figure S14. LX-2 cells were activated with TGF-β (10 ng/ml) and exposed to compound **1** or **2** at the concentration of 10 μM for 24 h. Quantitative real-time PCR analysis of expression of genes correlated to the activation of stellate cells Tgf-β, α-Sma, and Col1α1. Data are normalized to GAPDH. Results represent mean ± SEM, $^{*}p < 0.05$ vs TGF-β group.

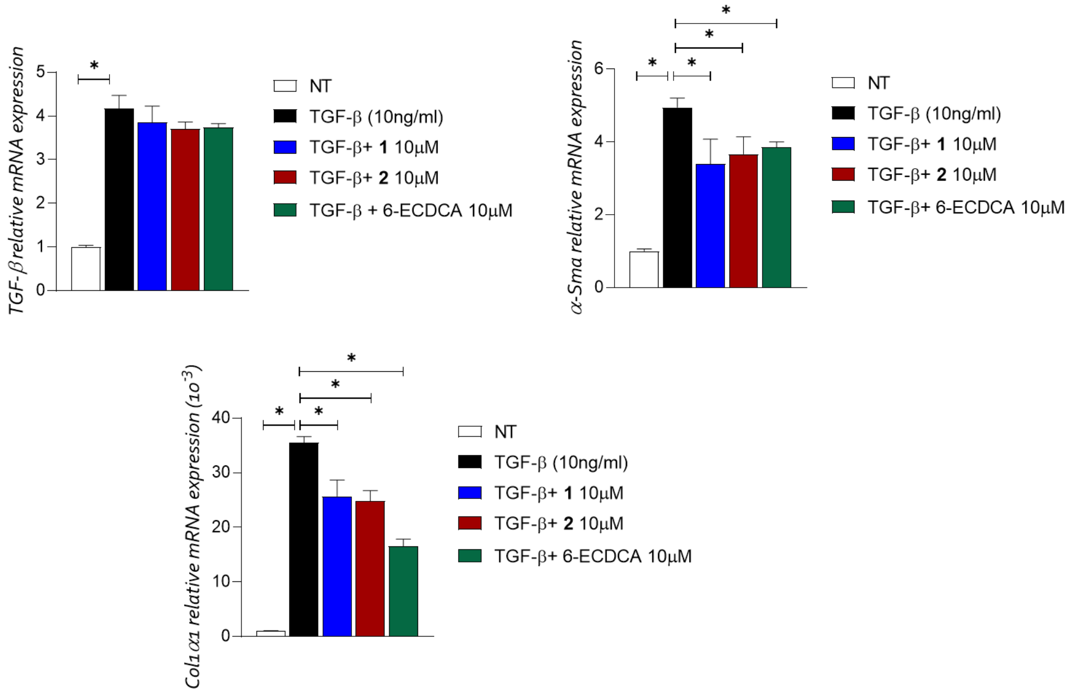

Figure S15. ^{1}H NMR ($CDCl_3$, 400 MHz) spectrum of new compound **6**

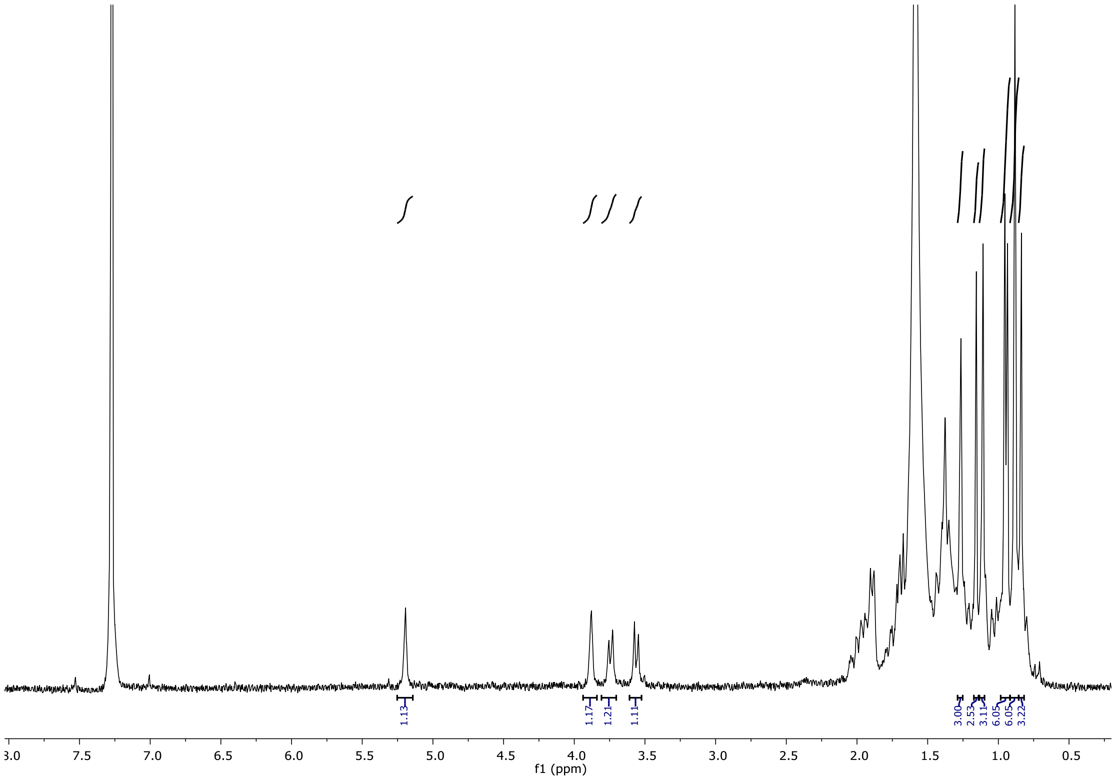

Figure S16. HSQC ($CDCl_3$, 400 MHz) spectrum of new compound **6**

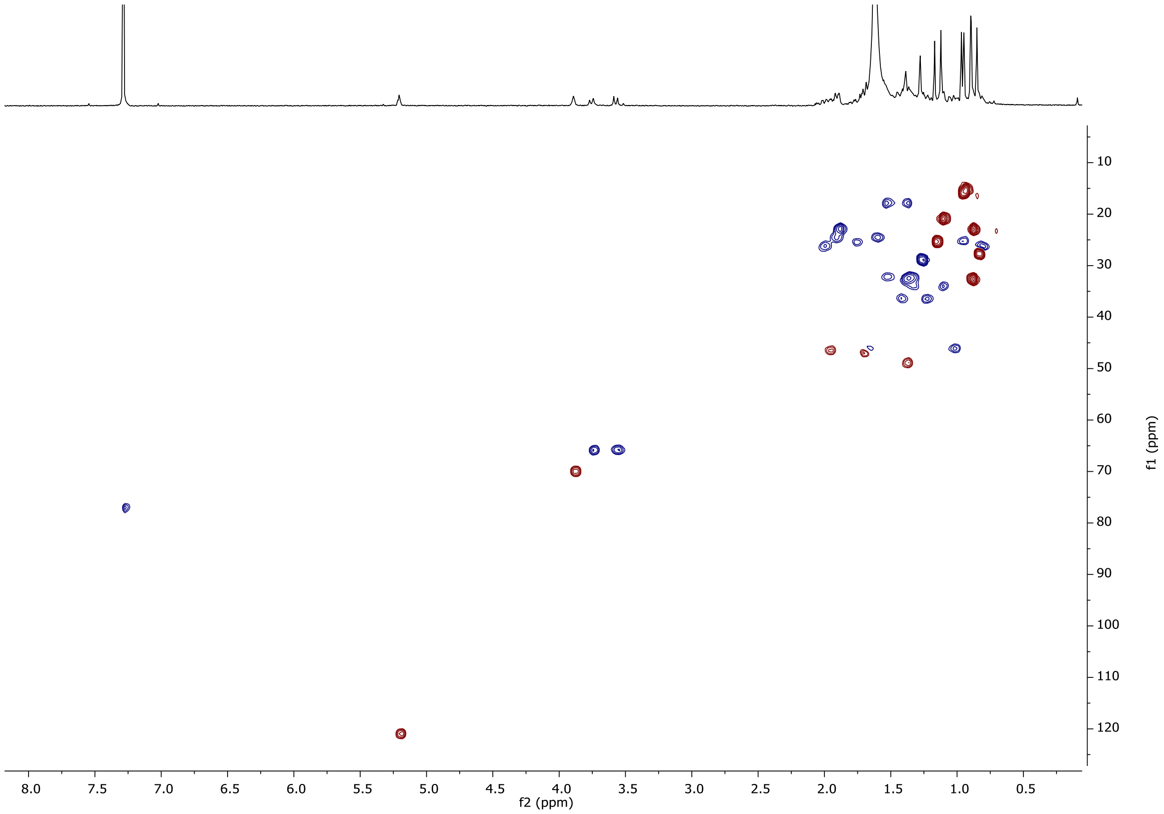

Figure S17. ROESY ($CDCl_3$, 400 MHz) spectrum of new compound **6**

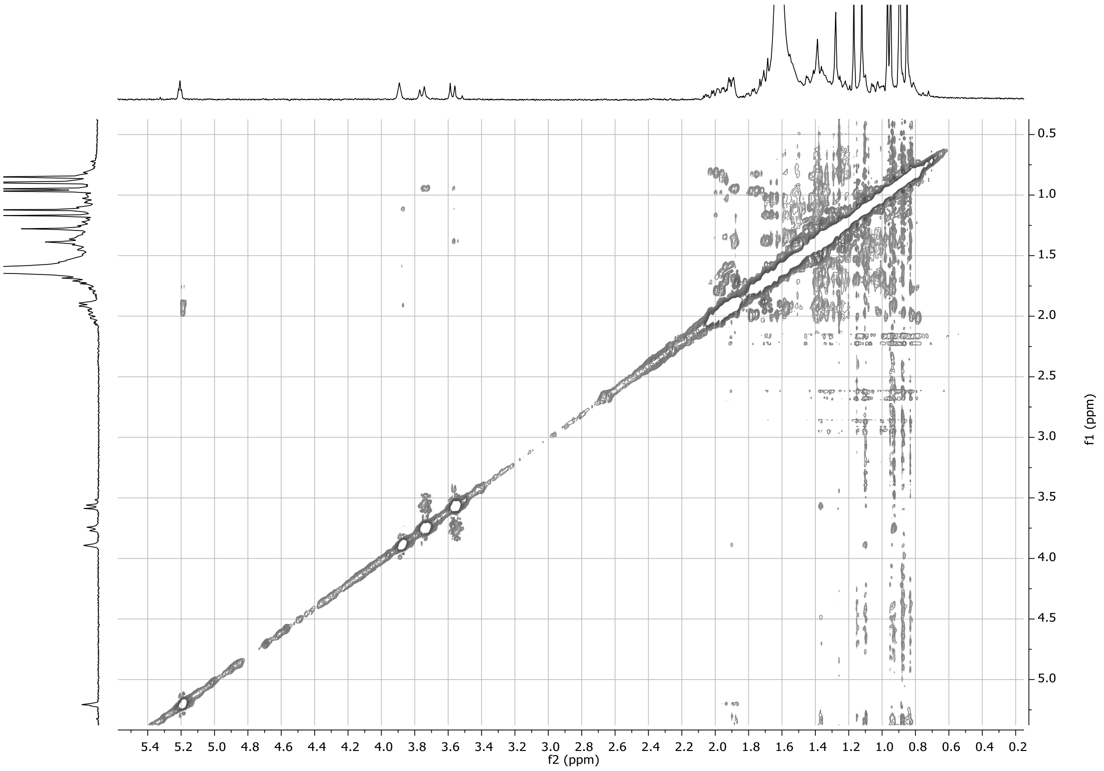

Figure S18. ^1H NMR ($CDCl_3$, 400 MHz) spectrum of new compound **20**

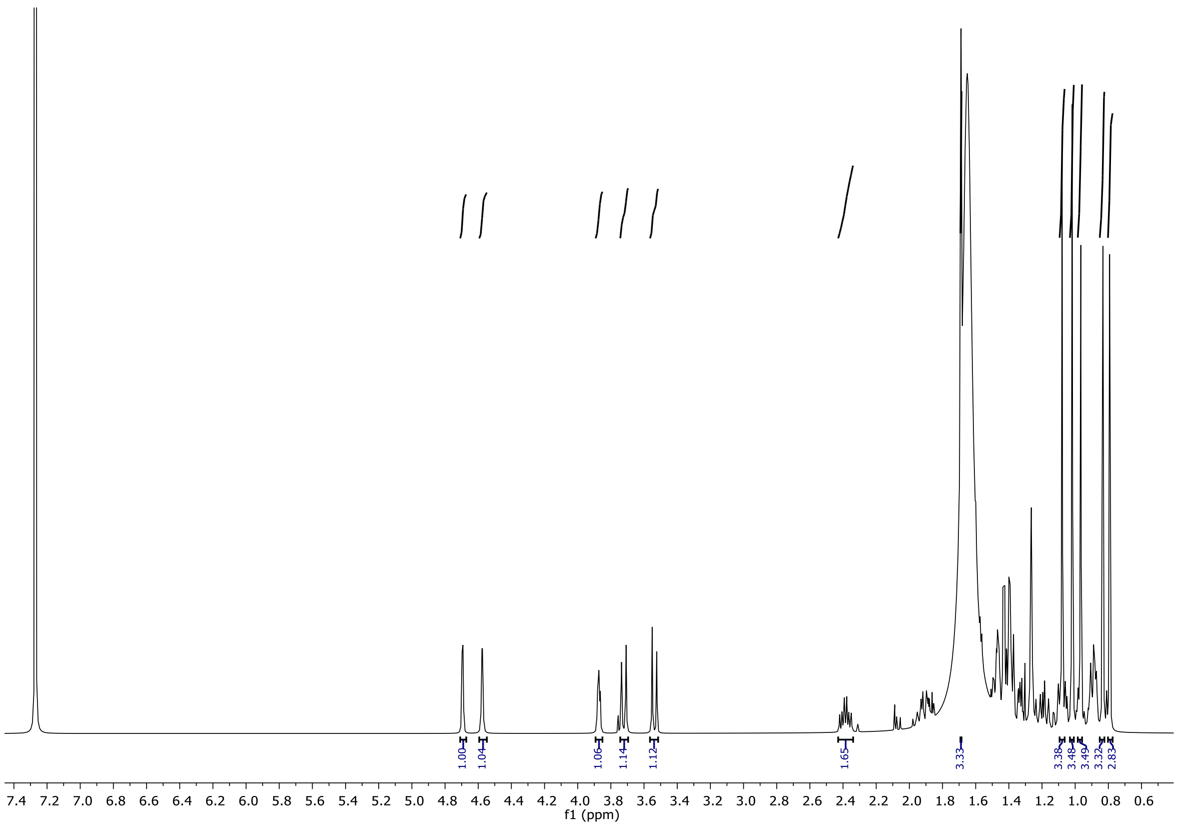

Figure S19. HSQC ($CDCl_3$, 400 MHz) spectrum of new compound **20**

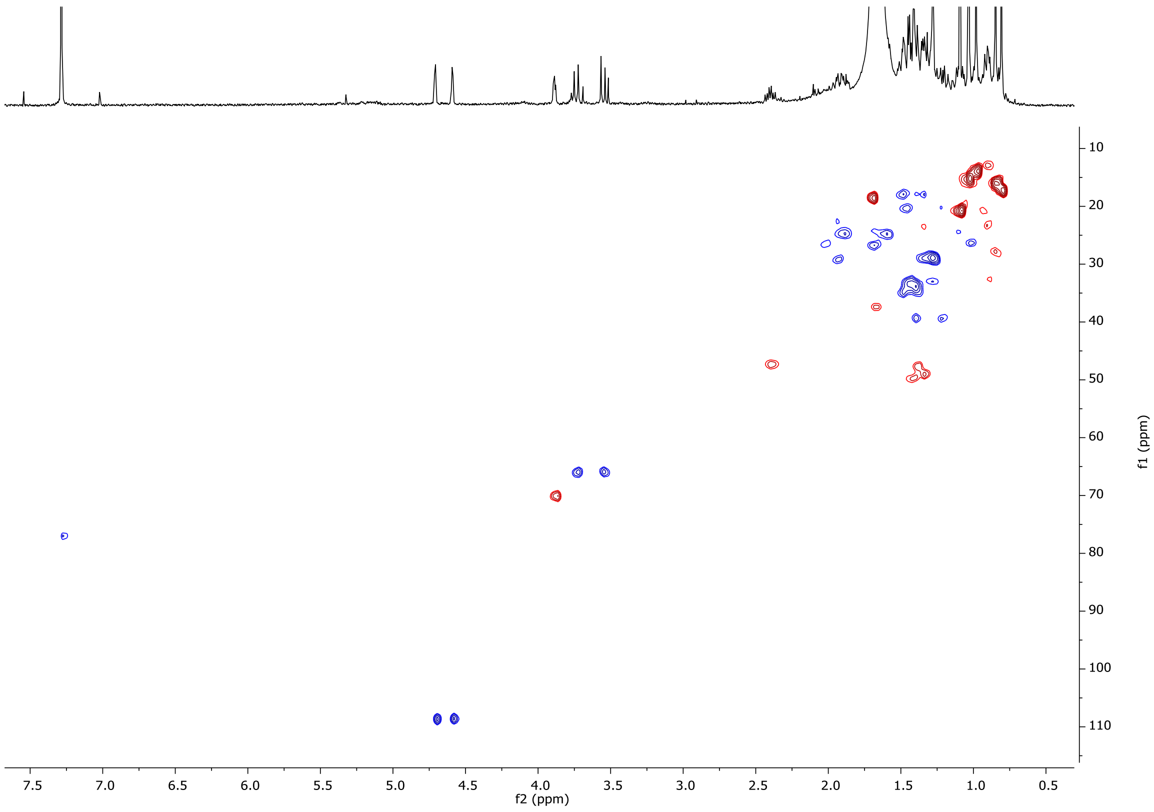

Figure S20. HMBC ($CDCl_3$, 400 MHz) spectrum of new compound **20**

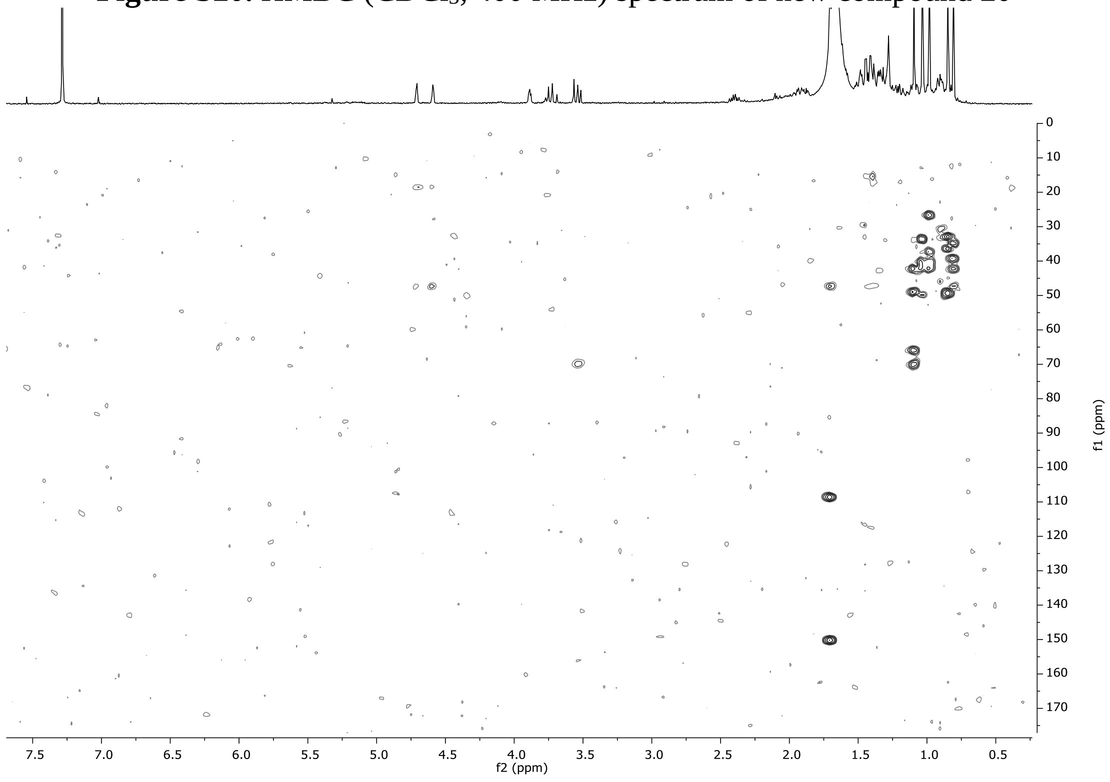

Figure S21. ROESY ($CDCl_3$, 400 MHz) spectrum of new compound **20**

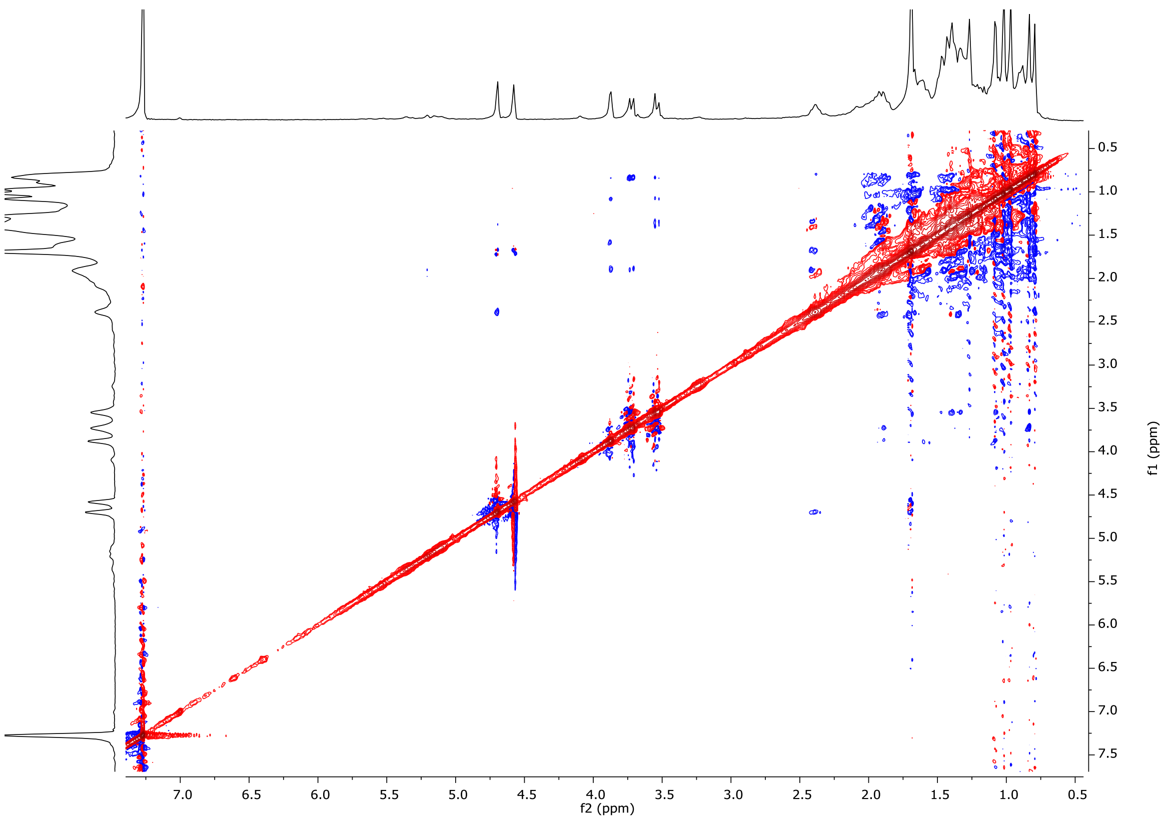

Figure S22. ^{1}H NMR (CD_3OD, 400 MHz) spectrum of compound **21**

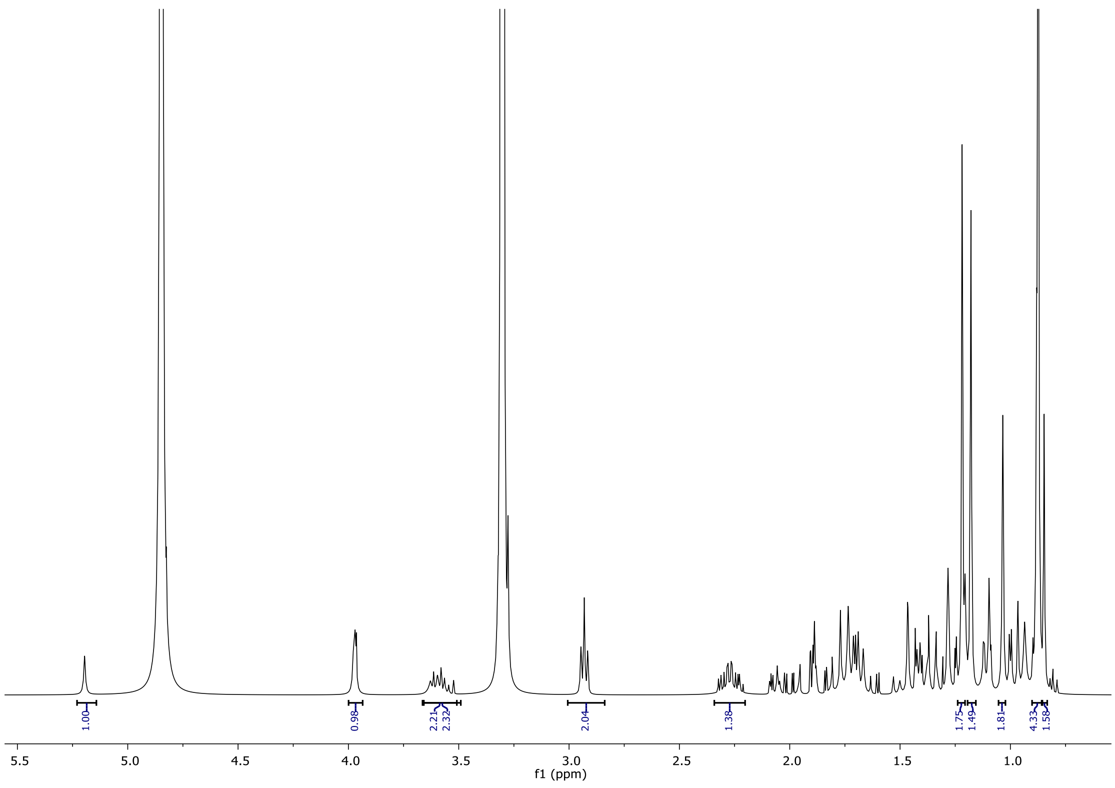

Figure S23. ^{13}C NMR (CD_3OD, 100 MHz) spectrum of compound **21**

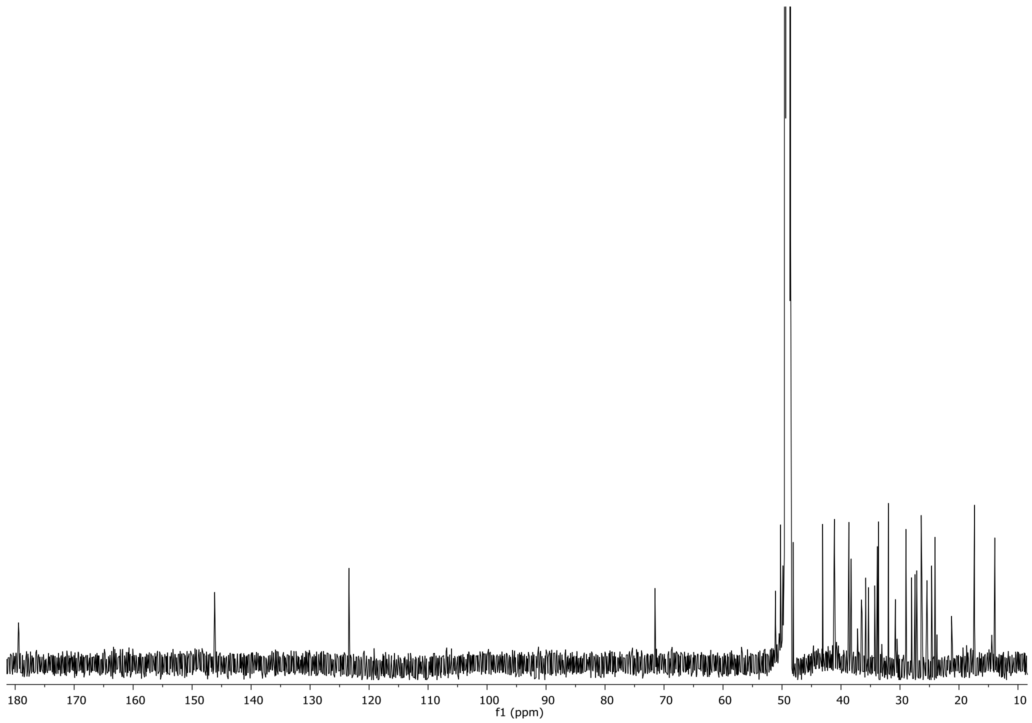

Figure S24. ^{1}H NMR (CD_3OD, 400 MHz) spectrum of compound **22**

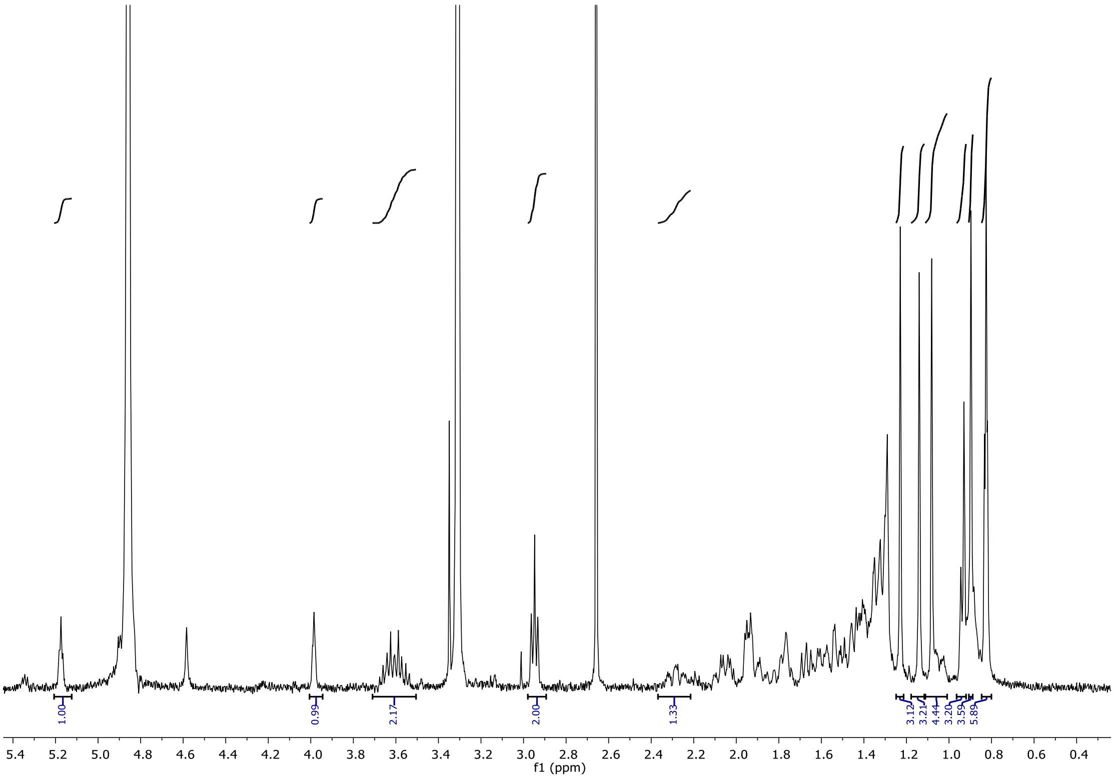

Figure S25. HSQC (CD_3OD, 400 MHz) spectrum of compound **22**

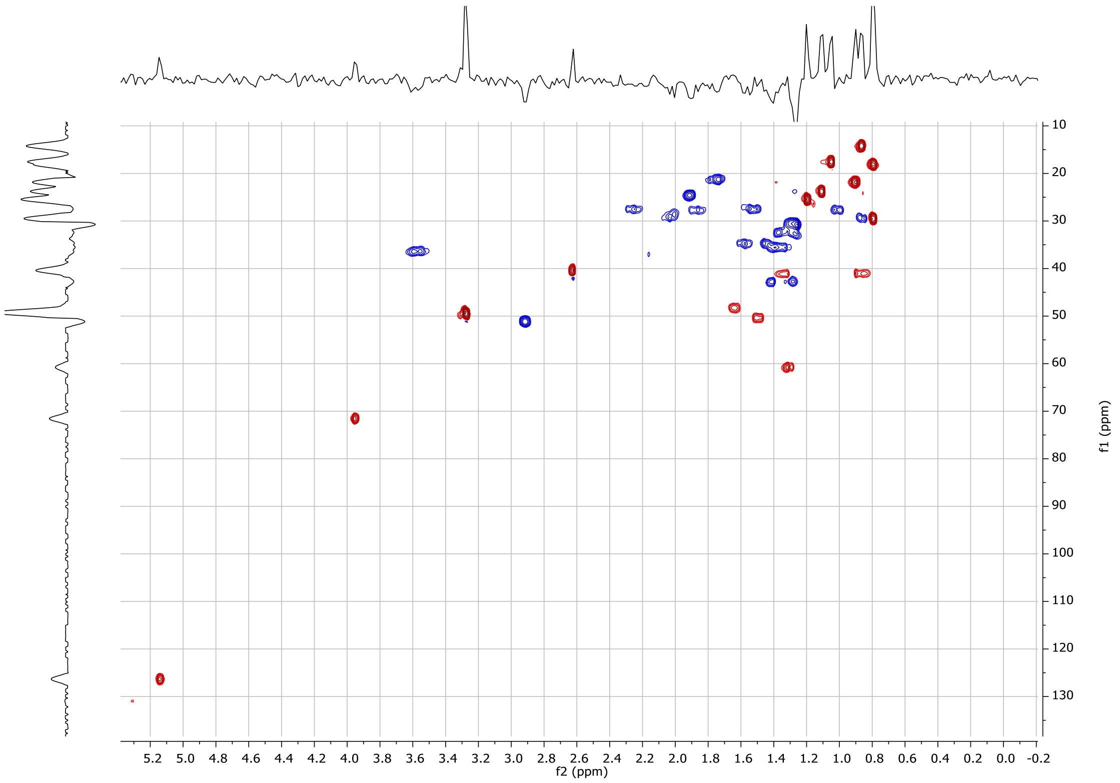

Figure S26. HMBC (CD_3OD, 400 MHz) spectrum of compound **22**

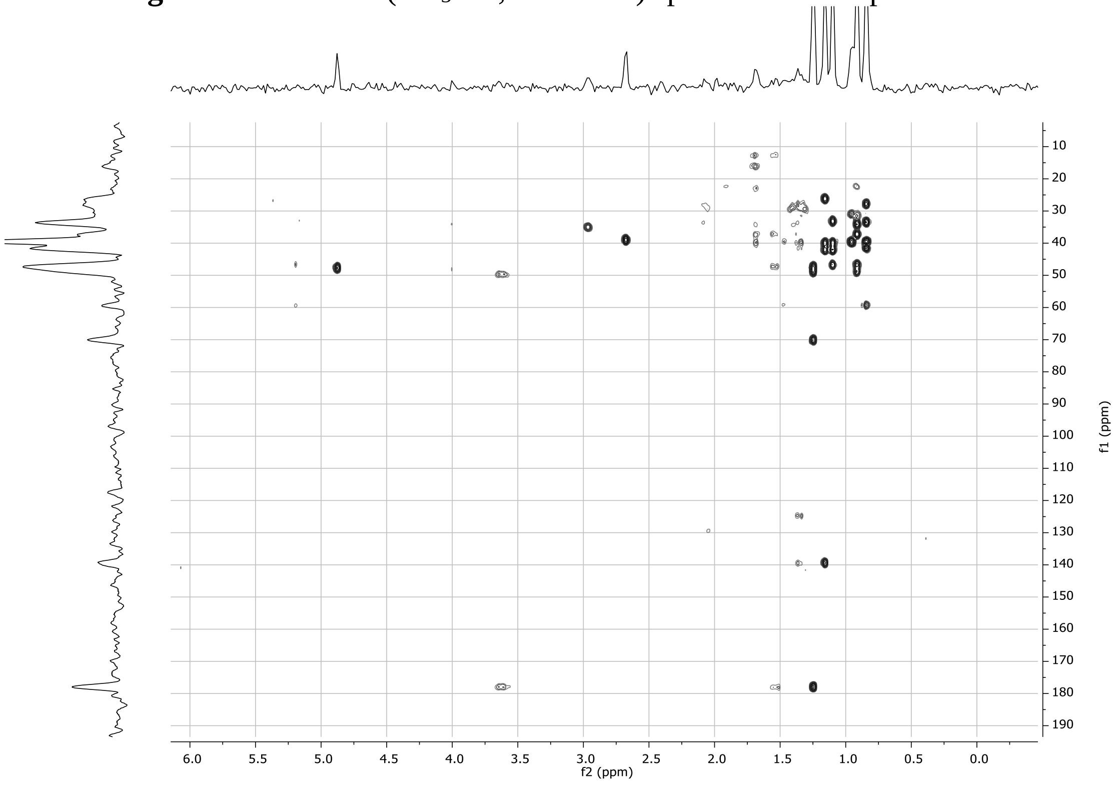

Figure S27. COSY (CD_3OD, 400 MHz) spectrum of compound **22**

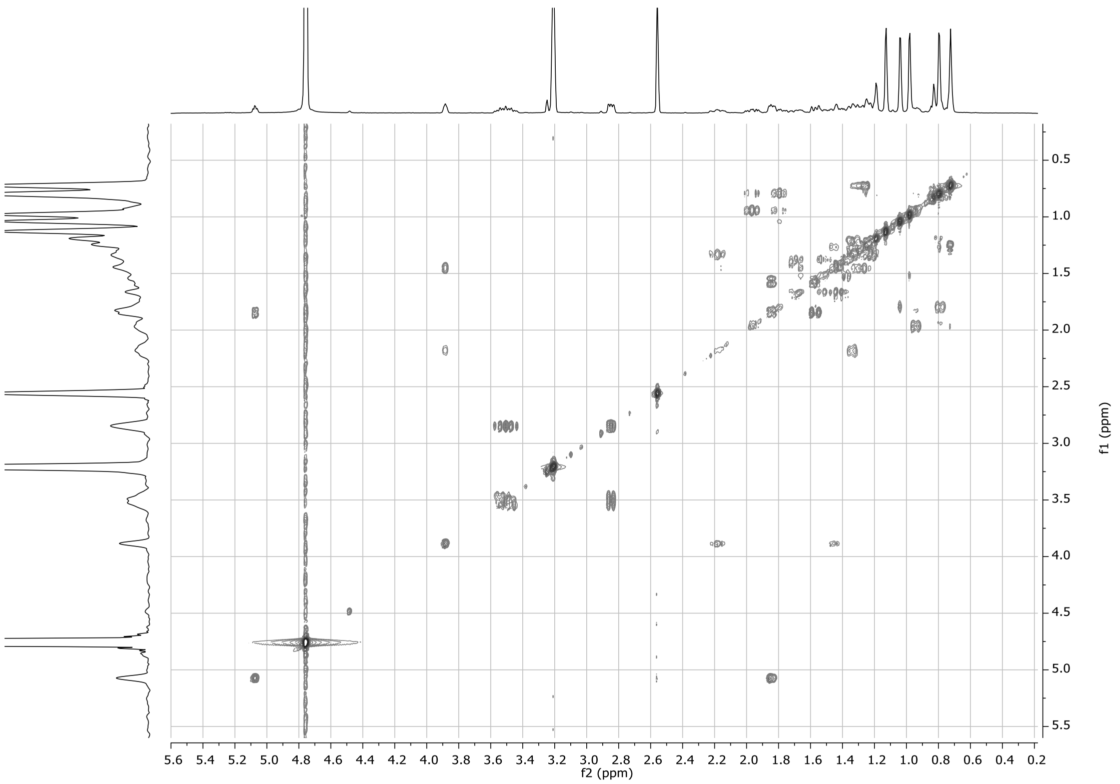

Figure S28. ^{1}H NMR (CD_3OD, 400 MHz) spectrum of compound **23**

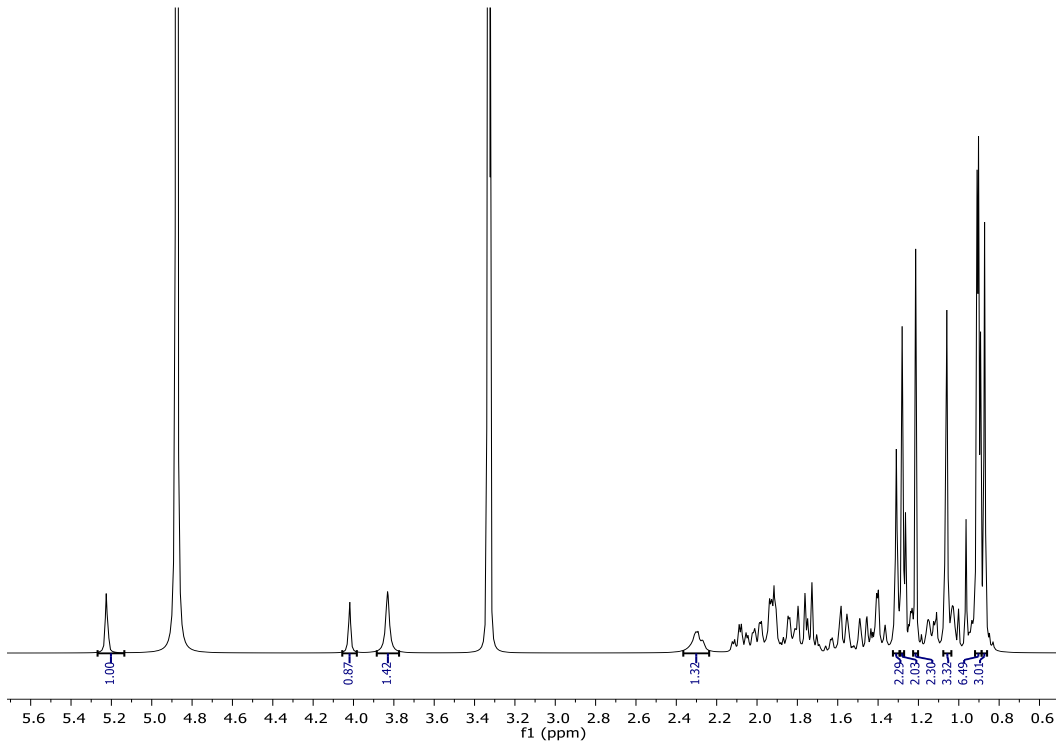

Figure S29. HSQC (CD_3OD, 400 MHz) spectrum of compound **23**

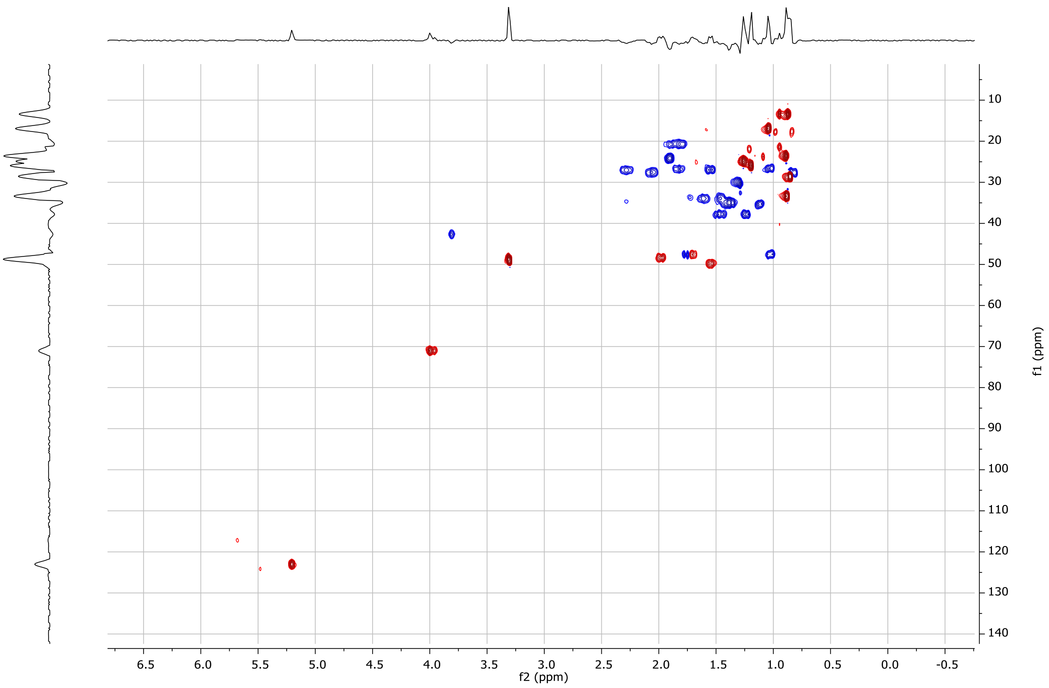

Figure S30. HMBC (CD_3OD, 400 MHz) spectrum of compound **23**

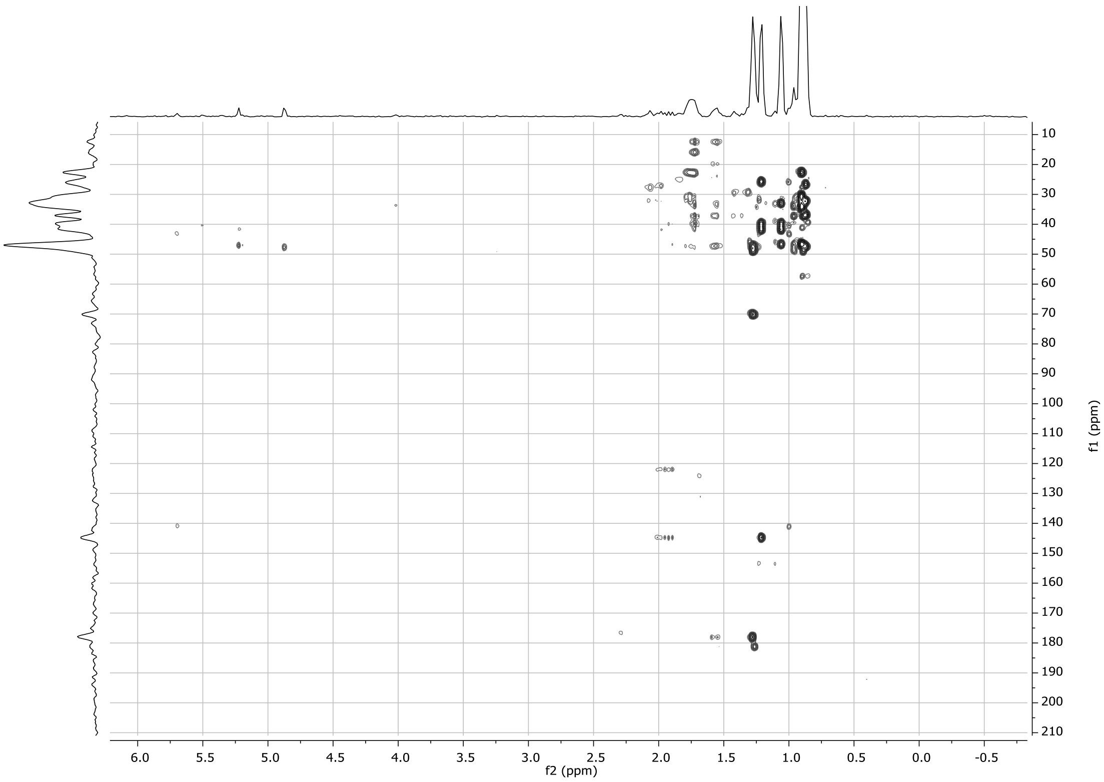

Figure S31. COSY (CD_3OD, 400 MHz) spectrum of compound **23**

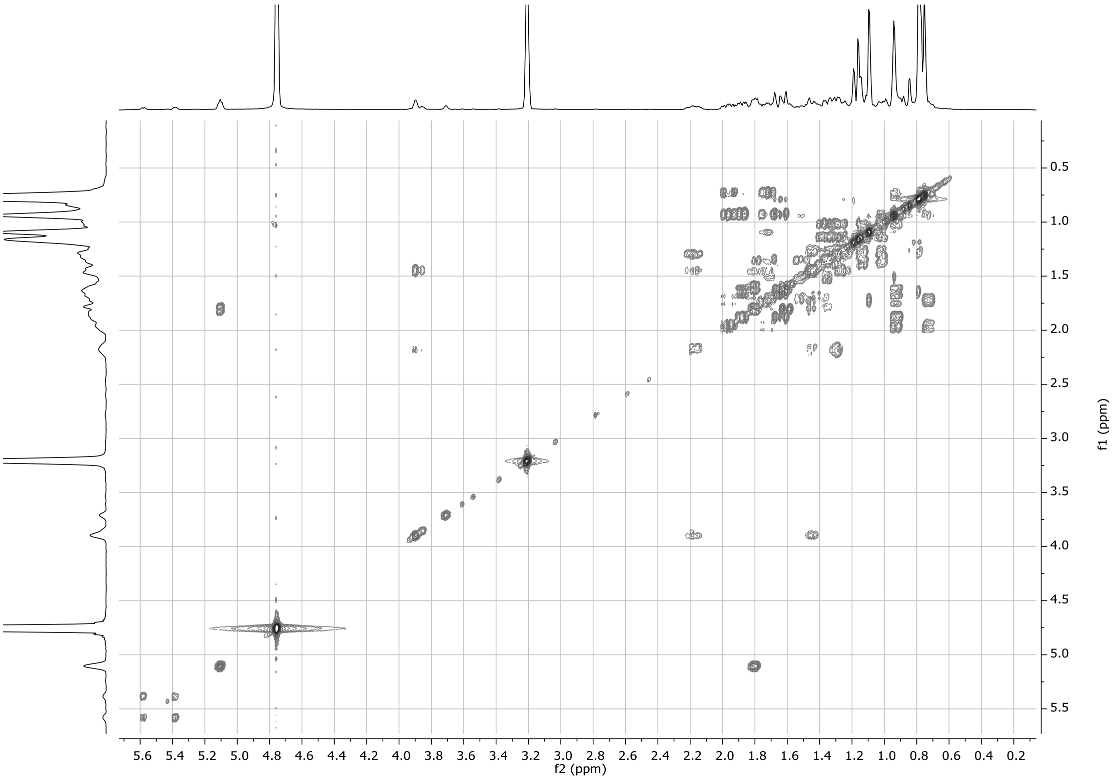

Figure S32. ^{1}H NMR (CD_3OD, 400 MHz) spectrum of compound **24**

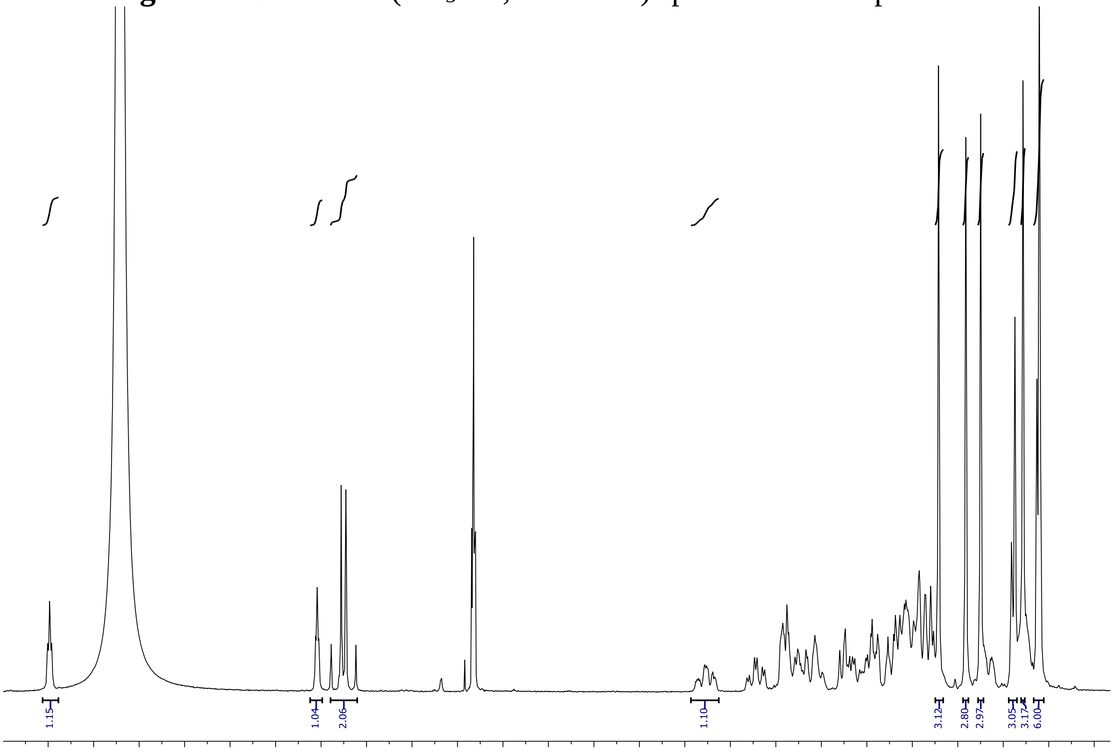

Figure S33. HSQC (CD_3OD, 400 MHz) spectrum of compound **24**

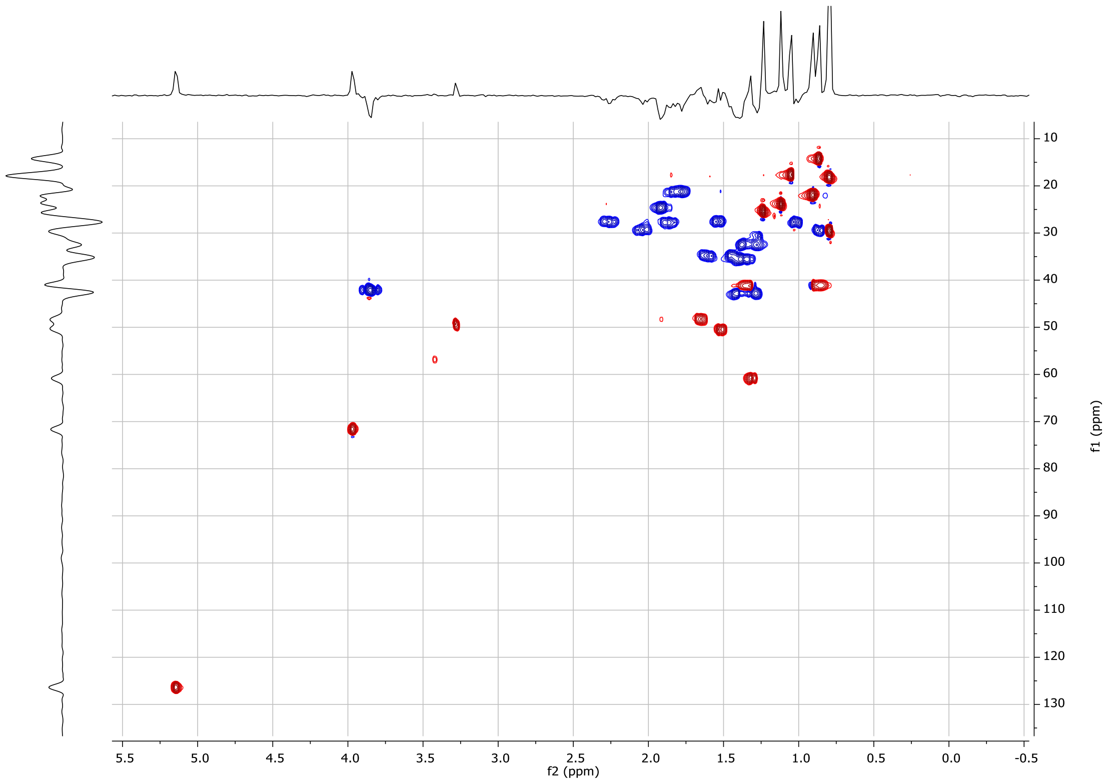

Figure S34. HMBC (CD_3OD, 400 MHz) spectrum of compound **24**

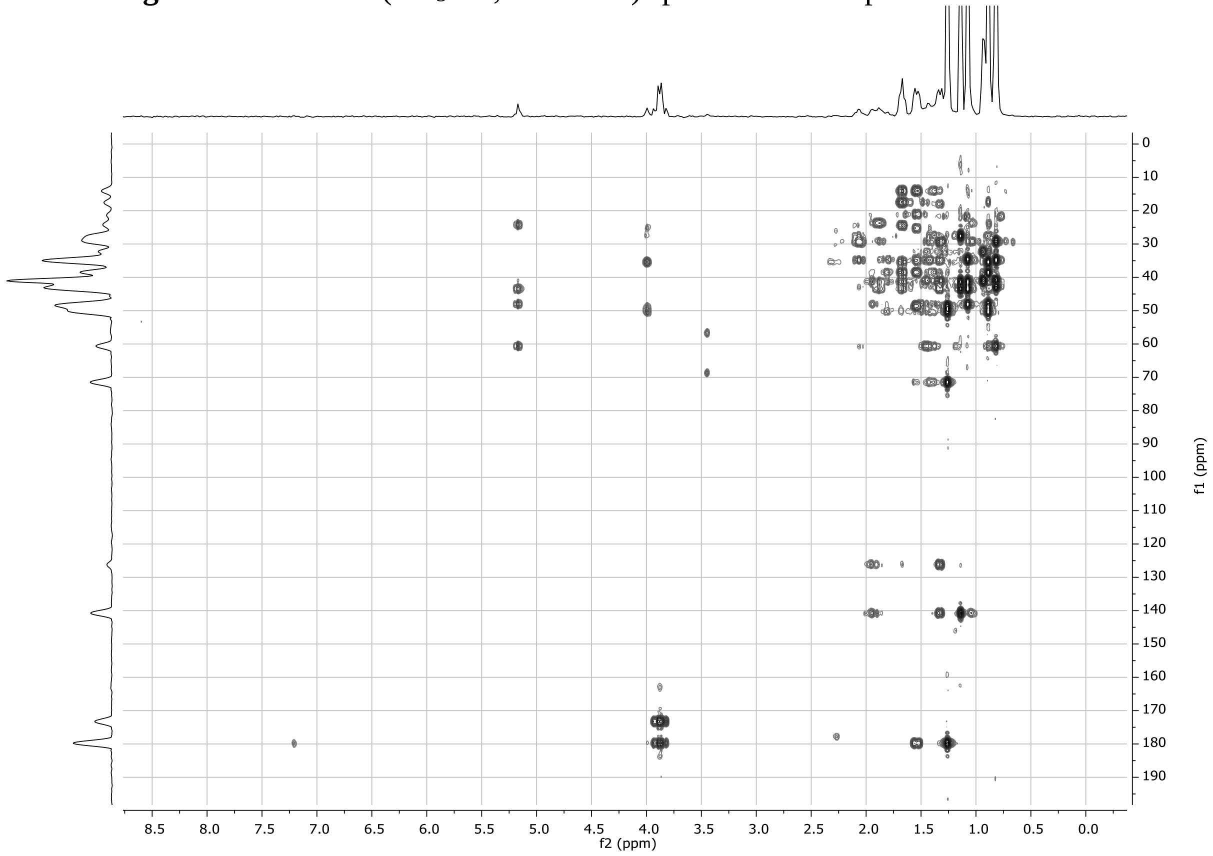

Figure S35. COSY (CD_3OD, 400 MHz) spectrum of compound **24**

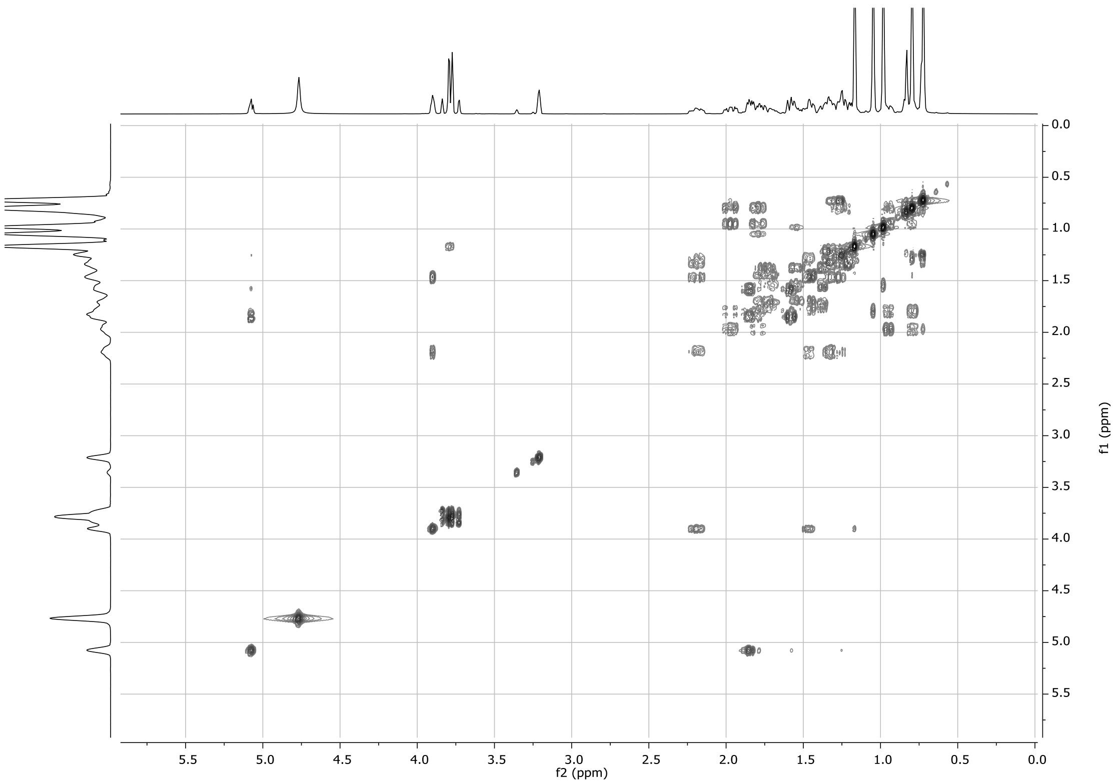

Supplement: Supplementary file 1 [file ao5c03492_si_001.pdf]
